# Cardiovascular “Patterns” of H_2_S and SSNO^−^-Mix Evaluated from 35 Rat Hemodynamic Parameters

**DOI:** 10.3390/biom11020293

**Published:** 2021-02-16

**Authors:** Lenka Tomasova, Marian Grman, Anton Misak, Lucia Kurakova, Elena Ondriasova, Karol Ondrias

**Affiliations:** 1 Biomedical Research Center, Institute of Clinical and Translational Research, Slovak Academy of Sciences, 811 04 Bratislava, Slovakia; lenka.tomasova@savba.sk (L.T.); marian.grman@savba.sk (M.G.); anton.misak@savba.sk (A.M.); 2 Department of Pharmacology and Toxicology, Faculty of Pharmacy, Comenius University, 814 99 Bratislava, Slovakia; kurakova4@uniba.sk (L.K.); ondriasova@fpharm.uniba.sk (E.O.)

**Keywords:** sulfide, nitric oxide, nitrosopersulfide, arterial pulse waveform, blood pressure, hemodynamic parameters, signaling pathways

## Abstract

This work is based on the hypothesis that it is possible to characterize the cardiovascular system just from the detailed shape of the arterial pulse waveform (APW). Since H_2_S, NO donor S-nitrosoglutathione (GSNO) and their H_2_S/GSNO products (SSNO^−^-mix) have numerous biological actions, we aimed to compare their effects on APW and to find characteristic “patterns” of their actions. The right jugular vein of anesthetized rats was cannulated for i.v. administration of the compounds. The left carotid artery was cannulated to detect APW. From APW, 35 hemodynamic parameters (HPs) were evaluated. H_2_S transiently influenced all 35 HPs and from their cross-relationships to systolic blood pressure “patterns” and direct/indirect signaling pathways of the H_2_S effect were proposed. The observed “patterns” were mostly different from the published ones for GSNO. Effect of SSNO^−^-mix (≤32 nmol kg^−1^) on blood pressure in the presence or absence of a nitric oxide synthase inhibitor (L-NAME) was minor in comparison to GSNO, suggesting that the formation of SSNO^−^-mix in blood diminished the hemodynamic effect of NO. The observed time-dependent changes of 35 HPs, their cross-relationships and non-hysteresis/hysteresis profiles may serve as “patterns” for the conditions of a transient decrease/increase of blood pressure caused by H_2_S.

## 1. Introduction

The information obtained from arterial pulse waveform (APW) analysis can provide insight into the function of the cardiovascular system in physiological and patho-physiological conditions. Several APW parameters were shown to be useful for the characterization of the cardiovascular system [1,2,3]. Recently, we introduced the evaluation of 35 parallel time-dependent rat hemodynamic parameters (HPs) from recorded APW at a high resolution [4,5,6]. From the cross-relationships of the 35 HPs, one can obtain 595 “patterns” characterizing cardiovascular system at the given conditions. In the cases when changes of HPs are transient, non-hysteresis/hysteresis “patterns” can be evaluated from the cross-relationships that may indicate direct or indirect connections of signaling pathways between two given HPs [4,5].

The present work is a continuation of our work based on the hypothesis that it is possible to characterize the cardiovascular system in many patho-physiological conditions from the detailed shape of APW. The HPs and their cross-relationships may provide “patterns” for particular cardiovascular conditions, as we obtained the conditions of decrease/increase of NO bioavailability [4,5,6].

Since endogenously produced gasotransmitters, hydrogen sulfide (H_2_S) and nitric oxide (NO), and the products of H_2_S/NO interactions (referred as SSNO^−^-mix) influence various physiological processes, particularly regulate blood pressure (BP) [7,8,9,10,11,12,13,14,15,16,17], it was of interest to study the effects of H_2_S and SSNO^−^-mix on 35 HPs and subsequently compare the selected cross-relationships of the HPs and their non-hysteresis/hysteresis “patterns” to the published data for NO-donor S-nitrosoglutathione (GSNO) [4].

We found that the intravenous (i.v.) administration of H_2_S transiently influenced all 35 HPs in anesthetized rats and from the cross-relationships of the HPs to the systolic BP, “patterns” and direct/indirect signaling pathways of the H_2_S effects on hemodynamics were proposed. The observed “patterns” after H_2_S administration were mostly different from the “patterns” observed after GSNO administration [4]. The effect of SSNO^−^-mix (≤32 nmol kg^−1^) on BP in the presence or absence of an inhibitor of nitric oxide synthase L-NAME was minor in comparison to GSNO, suggesting that the formation or presence of SSNO^−^-mix products in blood may diminish the hemodynamic effects of NO.

## 2. Materials and Methods

### 2.1. Chemicals

Na_2_S was purchased from DoJindo (SB01, Munich, Germany), and other chemicals were purchased from Sigma-Aldrich (Steinheim, Germany). Na_2_S dissociates in the solution and reacts with H^+^ to yield H_2_S, HS^−^ and traces of S^2−^. We use the term “H_2_S” to encompass the total mixture of H_2_S, HS^−^ and S^2−^. Stock solution of 100 mM Na_2_S was prepared in argon-bubbled ultrapure deionized H_2_O and stored at −80 °C. It was thawed and diluted with 0.9% NaCl to the final concentration just before the i.v. administration. GSNO was freshly prepared in 0.9% NaCl before i.v. administration from a stock 10 mmol L^−1^ GSNO prepared in ultrapure deionized water and stored at −80 °C. The products of the H_2_S/GSNO interaction (SSNO^−^-mix) were prepared by mixing of 20 µL of 10 mmol L^−1^ GSNO (in H_2_O) and 20 µL of 100 mmol L^−1^ Na_2_S (in H_2_O) in 160 µL buffer (200 mmol L^−1^ Tris.HCl, pH 7.4) at 22 ± 1.5 °C. Product formation was monitored by UV-VIS spectrophotometry (absorbance increase at λ_max_ 412 nm corresponds to the SSNO^−^ formation), and the reaction was completed within 3 min, followed by the dilution of the mixture with 0.9% NaCl to the final concentration just before the i.v. administration. Since SSNO^−^ was proposed to be the major longer-lived product of the sulfide and S-nitrosothiols reaction [14,18,19], we hereinafter refer to the products of the reaction mixture as the “SSNO^−^-mix”. The concentration of the SSNO^−^-mix is defined as the initial concentration of GSNO in the mixture when the reaction time equals zero.

### 2.2. Ethical Approval

All procedures were approved by the State Veterinary and Food Administration of the Slovak Republic (C.k. Ro 3123/17-221) according to the guidelines from Directive 2010/63/EU of the European Parliament. The procuration of animals, the husbandry and the experiments conformed to the “European Convention for the Protection of Vertebrate Animals used for Experimental and other Scientific Purposes” (Council of Europe No 123, Strasbourg 1985). Experiments were carried out according to the guidelines laid down by the animal welfare committee of the Biomedical Research Center, Slovak Academy of Sciences, Bratislava and conformed to the principles and regulations, as described in the Editorial by Grundy [20]. The animals were under anesthesia throughout the duration of the experiment and were euthanized with an overdose of Zoletil/Xylazine via the jugular vein at the end of the surgical procedure.

### 2.3. Animals, APW Measurement and Data Evaluation

**Figure 1:**
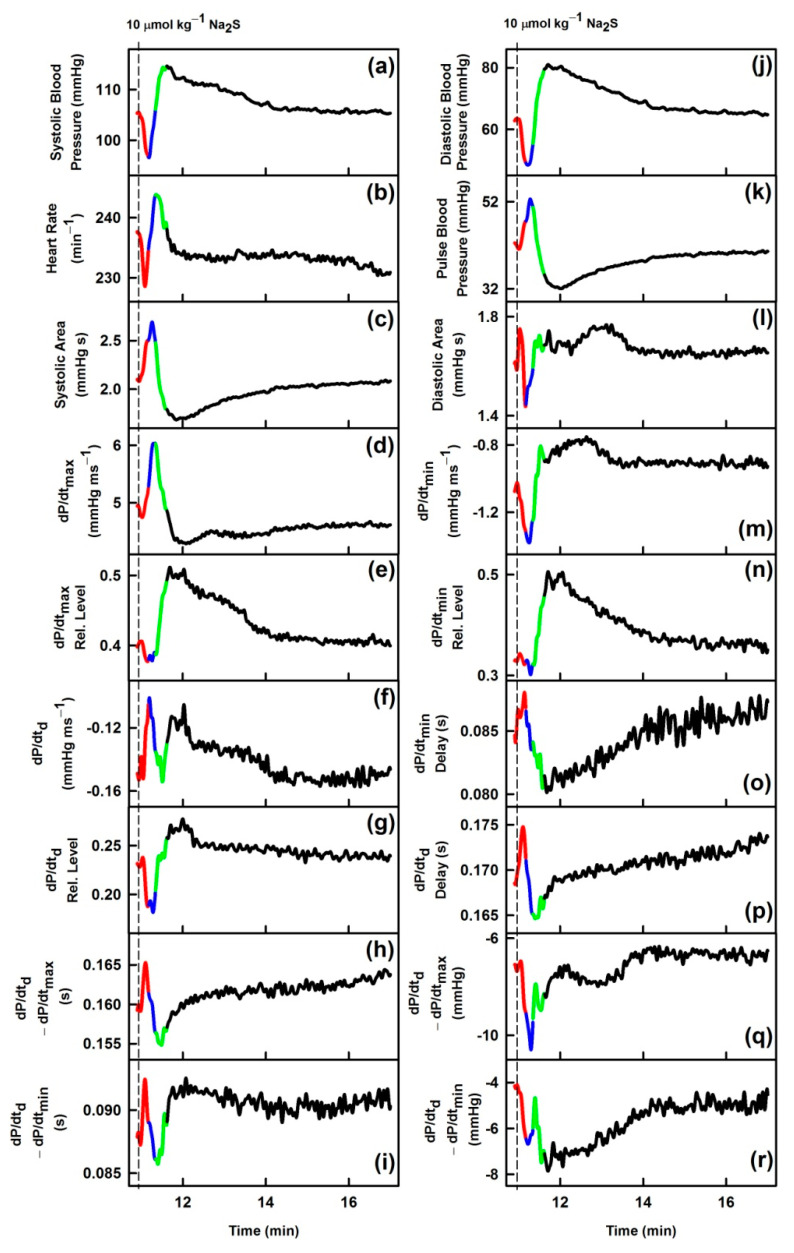

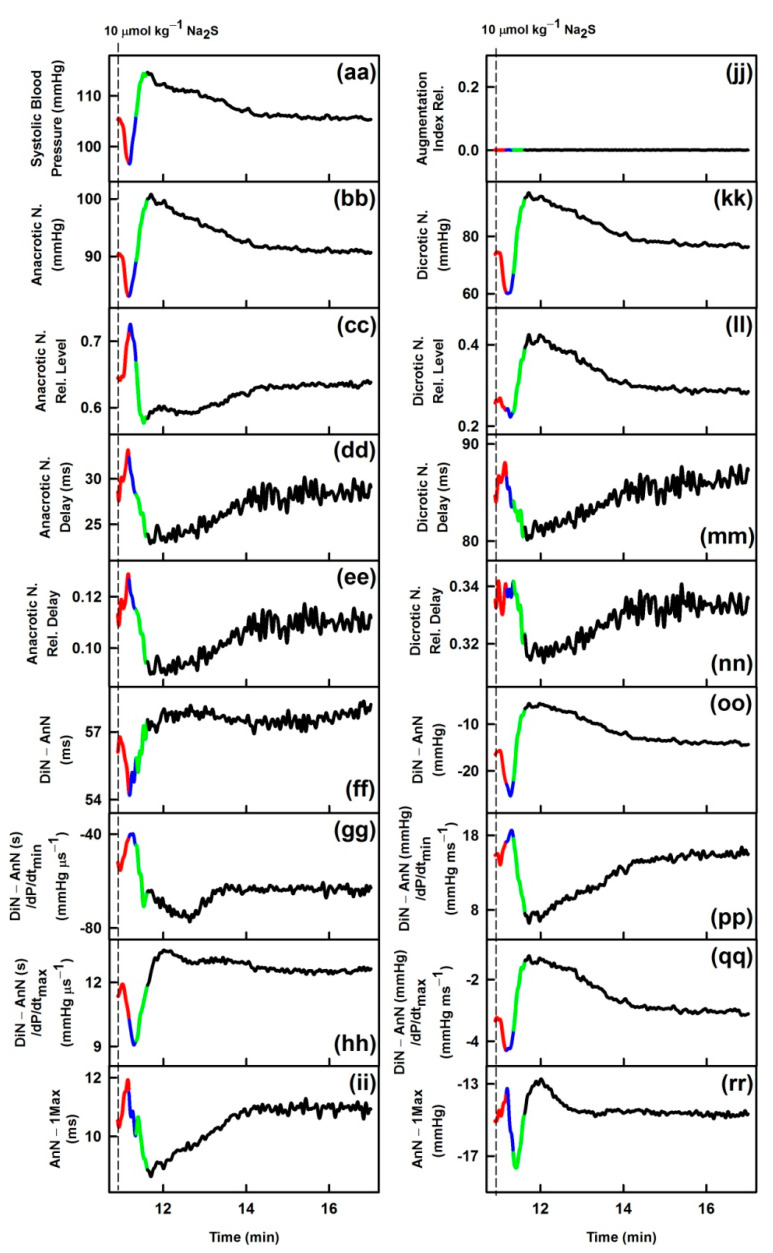
Time-dependent changes of hemodynamic parameters (HPs) of anesthetized rat after i.v. bolus administration of 10 µmol kg^−1^ Na_2_S (marked by dash lines). The red line starts 3 s before Na_2_S administration. Colors: time period corresponding to the decrease of systolic BP (red), increase of systolic BP to the control value (blue), further increase of systolic BP to the maximum (green) and decrease of systolic BP to the control value (black). For the explanation of (**a**), (**b**) … (**rr**) plots see the Description of the Table 1 and Figure S1. Units in plots (**gg**), (**hh**), (**pp**) and (**qq**) are informative only.

The method was essentially described in our previous studies [4,5,6]. We follow the principle of not implementing any more animals as is necessary in order to minimize animal use according to the principles of the National Centre for the Replacement, Refinement and Reduction of Animals in Research (NC3Rs, London, United Kingdom). Male Wistar rats (n = 17; 330 ± 40 g) were purchased from the Department of Toxicology and Laboratory Animal Breeding at Dobra Voda, Slovak Academy of Sciences, Slovakia. The rats were housed under a 12 h-light–12 h-dark cycle, at a constant humidity (45–65%) and temperature (20–22 °C), with free access to standard laboratory rat chow and drinking water. The veterinary nursing care was provided by the Central Animal Housing Facility of Pavilion of Medical Sciences (registration number SK UCH 01017). In the experiments with 10 µmol kg^−1^ H_2_S, eight rats were anesthetized with Zoletil 100 (tiletamine + zolazepam, 80 mg kg^−1^, i.p.) and Xylazine (5 mg kg^−1^, i.p.). In the experiments, where we compared the effects of H_2_S, GSNO and SSNO^−^-mix on the same rat, long time (~100 min) anesthesia was necessary. Therefore, nine rats were anesthetized with Zoletil 100 (tiletamine + zolazepam, 60 mg kg^−1^, i.p.) and Xylazine (5 mg kg^−1^, i.p.), and just before surgical operation, the same anesthetic dose were administrated again. For notice, H_2_S, GSNO or SSNO^−^-mix were administered into the right jugular vein (500 µL kg^−1^) over a 15 s period, about 40–50 min after the first anesthetic application. L-NAME (25 mg kg^−1^, prepared in 0.9% saline solution) was used to study the hemodynamic effects of H_2_S, GSNO or SSNO^−^-mix in the condition of low NO bioavailability and high BP. The left common carotid artery (*arteria carotis communis*) was cannulated to insert the fiber-optic microcatheter pressure transducer FISO LS 2F connected to the FISO Series Signal Conditioner embedded in the EVO Chassis (Harvard Apparatus, Holliston, MA, USA). The recorded analog APW signal was filtered by a lowpass filter at 1 kHz, digitalized at 10 kHz and stored on a computer. Ten points (a–j) of APW, marked in Figure S1, were analyzed, from which 35 HP parameters were calculated as in our published studies [4,5,6]. Definition and abbreviation of the 35 HP parameters are described in detail in the Supplementary Materials (Figure S1). Since point a, resp. j fluctuated with time interval ~5–10 ms and ~1 mmHg BP (Figure S1B), when it was necessary, the data were filtered to average the fluctuation. For better visual comparison, plots (a) and (aa) presenting systolic BP in Figures are the same. The plot of augmentation index relative (e.g., Figure 1jj) was not possible to determine in cases when the highest point at APW (Figure S1A) was “c” and not “f” (for details see Reference [4]).

## 3. Results

### 3.1. Effects of Na_2_S on Time-Dependent Changes of 35 HPs

The time-dependent changes of 35 HPs after i.v. bolus administration of 10 µmol kg^−1^ Na_2_S are shown in Figure 1 and for other seven rats in Figures S2–S8. Na_2_S affected all 35 HPs for the period of ~5–10 min and then the values of HPs returned to the control level. To compare the changes of the 35 HPs, the time dependency of all HPs was divided into four phases according to the systolic BP curve (Figure 1a): decrease of systolic BP (red), increase to the control value (blue), further increase to maximum BP (green) and final decrease to the control value (black). From the comparison of the phases, it is evident that most of the HPs did not follow the time-dependent changes of systolic BP after the administration of Na_2_S. Some of the HPs followed similar (but not identical) or reverse time-dependent changes as systolic BP (a). However, other HPs, e.g., systolic area (c), dP/dt_max_ (d), dP/dt_max_-RL (e), dP/dt_d_ (f), dP/dt_d_-RL (g), diastolic area (l), dP/dt_min_-RL (n), dP/dt_d_ − dP/dt_max_ (q), dP/dt_d_ − dP/dt_min_ (r), DiN-RD (nn), (DiN − AnN)/dP/dt_min_ (pp), and (DiN − AnN)/dP/dt_max_ (qq) showed a completely different course in time, suggesting that these APW changes are activated at different time points and probably by different signaling pathways than the changes in systolic BP.

### 3.2. Comparison of Transient Effects of Na_2_S with Published Data of GSNO on 35 HPs

In our previous studies, we evaluated time-dependent changes of 35 HPs in the condition of increased NO bioavailability by GSNO and compared these effects with the changes observed in the condition of decreased NO biovailability by L-NAME [4,5]. Since GSNO transiently decreased systolic BP, similarly to H_2_S, we compared trends of their effects on 35 HPs during the decrease of systolic BP (Table 1). The comparison revealed that the time-dependent trends of 25 HPs were similar for both compounds. The diastolic area (l) was affected in the opposite direction, GSNO decreased and Na_2_S increased this HP (red). The remaining 9 HPs showed different correlations, e.g., increase/decrease *vs* no effect for heart rate (b), dP/dt_max_ (d), dP/dt_min_-RL (n), dP/dt_d_ − dP/dt_min_ (r), AnN-RD (ee), (DiN − AnN)/dP/dt_max_ (hh), DiN-RD (nn) (blue); biphasic effect vs. no effect for dP/dt_d_ (f) (green) and biphasic effect *vs* increase for dP/dt_d_ − dP/dt_min_ (i) (brown). Reproducibility of the effects of Na_2_S on HPs was significantly lower than the reproducibility of the effects for NO-synthase inhibitor L-NAME and NO-donor GSNO [4,6].

### 3.3. Non-Hysteresis/Hysteresis Relationships between HPs to Systolic BP after Na_2_S Administration

**Figure 2:**
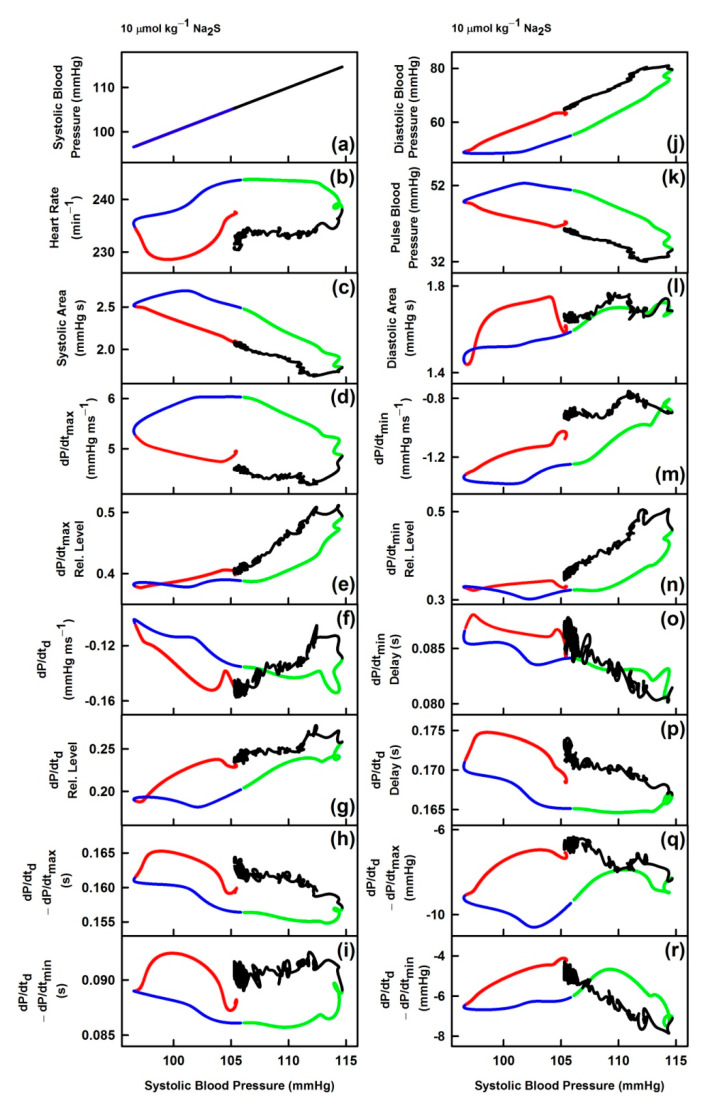

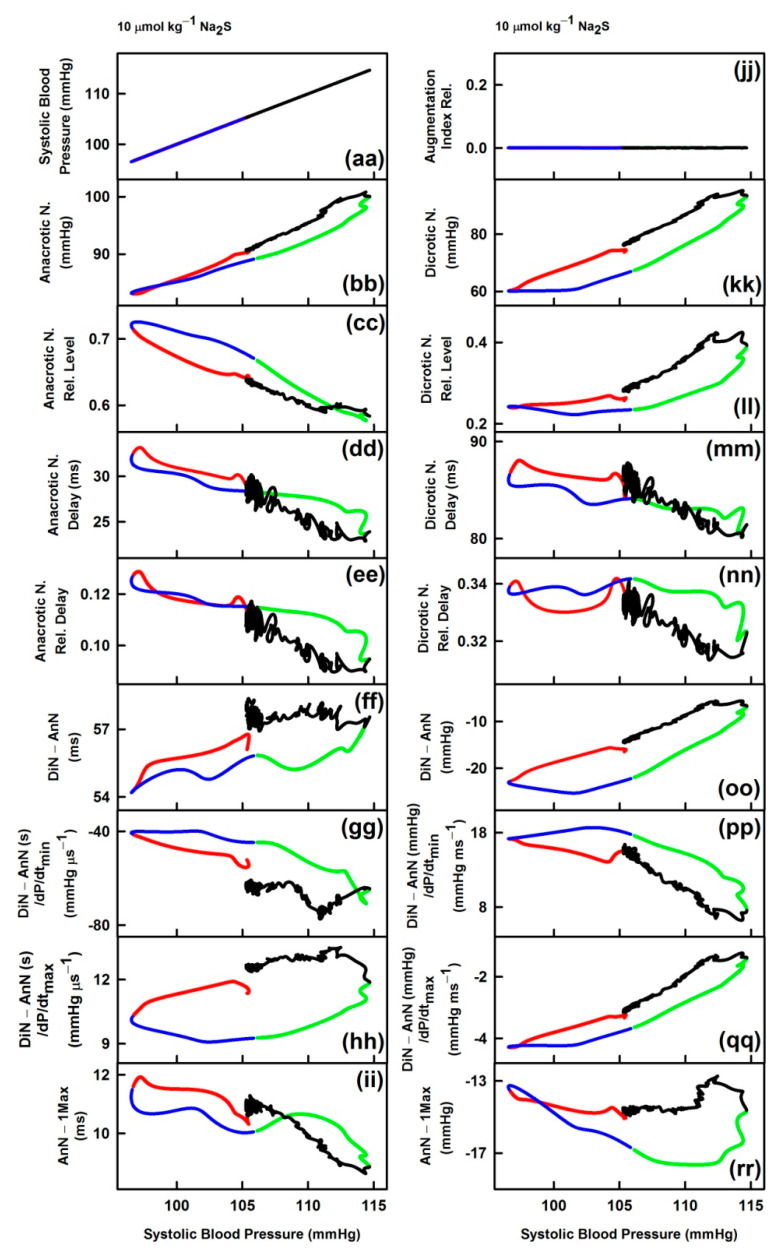
Relationships of HPs to the systolic BP after the administration of 10 µmol kg^−1^ Na_2_S. The colors and time-dependent data correspond to Figure 1. Time period corresponds to the decrease of systolic BP (red), increase of systolic BP to the control value (blue), further increase of systolic BP to maximum (green), and decrease of systolic BP to the control value (black). The hysteresis was arbitrarily defined as HP − systolic BP (in mmHg) loop > 5 mmHg of systolic BP. For the explanation of (**a**), (**b**) … (**rr**) plots see the Description of the Table 1 and Figure S1. Units in plots (**gg**), (**hh**), (**pp**) and (**qq**) are informative only.

In order to describe “patterns” of effects of Na_2_S on the cardiovascular system in more detail, time-dependent changes between systolic BP and other HPs, described as their cross-relationships, were evaluated. For the full description of the cross-relationships of the 34 HPs to systolic BP (mmHg) each plot in Figure 2 should be three-dimensional, which we found visually confusing. Therefore, we omitted the time dimension and presented two-dimensional cross-relationships only.

From the maximum sum of 595 derived relationships between each HP, the cross-relationships between 34 HPs and the systolic BP are shown only (Figure 2 and Figures S9–S15). The “patterns” were different for each relationship and their reproducibility for the eight rats was low. These cross-relationships showed hysteresis or non-hysteresis “patterns”. We evaluated the hysteresis/non-hysteresis pattern during the time period corresponding to the decrease (Figure 2; red lines) and increase of systolic BP (Figure 2; blue lines) after Na_2_S administration. The non-hysteresis cross-relationships mean that the changes of HP during the decrease (Figure 2; red lines) and increase of systolic BP (Figure 2; blue lines) followed approximately the same line in opposite directions (e.g., Figure 2; plots (dd) or (ee)). However, the hysteresis cross-relationships mean that the changes of HP during the decrease and increase of systolic BP did not follow the same line in opposite directions and form a loop > 5 mmHg of systolic BP (e.g., Figure 2; plots (p) or (q)). Similarly, one can reconsider the non-hysteresis/hysteresis cross-relationships of the response of the cardiovascular system to the administration of Na_2_S during the increase of systolic BP to maximum (Figure 2; green lines) and decrease of systolic BP to the control value (Figure 2; black lines).

**Figure 3:**
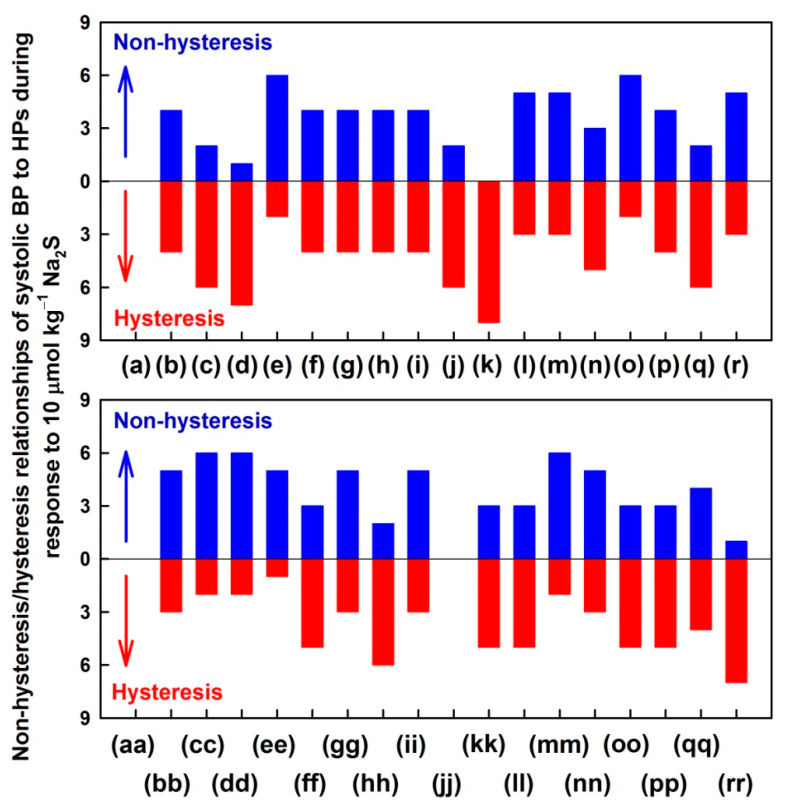
Number of rats, that represent the non-hysteresis or hysteresis “patterns” of the relationships of HPs to the systolic BP after the administrations of 10 µmol kg^−1^ Na_2_S during decrease (red line in Figure 1 and Figure 2) and increase (blue line in Figure 1 and Figure 2) of BP. Data were taken from Figure 2 and Figures S9–S15. The total number of rats in which non-hysteresis (blue) or hysteresis (red) “patterns” were evaluated is n = 8. The hysteresis was arbitrary defined as HP − systolic BP loop >5 mmHg of systolic BP. For the explanation of (**a**), (**b**) … (**rr**) plots see the Description of the Table 1 and Figure S1.

Figure 3 shows the number of rats, which represent the non-hysteresis or hysteresis cross-relationship of 34 HPs to the decrease/increase of systolic BP (data were taken from Figure 2 and Figures S9–S15, red and blue lines). There was a low reproducibility of the non-hysteresis/hysteresis cross-relationships in eight rats. The non-hysteresis cross-relationships (at least in 6 of 8 rats) were observed in plots: (e), (o), (cc), (dd) and (mm). The hysteresis cross-relationships (at least in 6 of 8 rats) were observed in plots: (c), (d), (j), (k), (q), (hh) and (rr).

Since H_2_S transiently decreased systolic BP, similarly to NO-donor GSNO, we compared the H_2_S data from Figure 3 to the published GSNO data (Figure 3c in Reference [4]). From the comparison of the H_2_S and GSNO non-hysteresis/hysteresis cross-relationship “patterns” of the 34 HPs to systolic BP, it is evident that most of the “patterns” are not the same—actually, some of them are opposite. For example, in the dP/dt_max_ to the systolic BP (plot (d)), hysteresis was observed in the case of H_2_S, but non-hysteresis in the case of GSNO response. Similarly, opposite non-hysteresis/hysteresis relationships were observed for diastolic BP (j), pulse BP (k) or dP/dt_d_ − dP/dt_max_ (q). However, similar non-hysteresis/hysteresis relationships for H_2_S and GSNO were observed for dP/dt_max_-RL (e), (DiN − AnN)/dP/dt_max_ (hh) or AnN − 1Max (rr).

Figure S16 represents the non-hysteresis/hysteresis “patterns” of the relationships of HPs to systolic BP, which were evaluated during the increase and decrease of BP (Figure 2 and Figures S9–S15, green and black lines, respectively) after the i.v. administration of 10 µmol kg^−1^ Na_2_S. The non-hysteresis cross-relationships (at least in 6 of 8 rats) were observed in plots: dP/dt_max_-RL (e), diastolic area (l), dP/dt_min_ (m), dP/dt_min_ delay (o), AnN-RL (cc), AnN delay (dd), AnN-RD (ee), (DiN − AnN)/dP/dt_min_ (gg), DiN delay (mm) and DiN-RD (nn). The hysteresis cross-relationships (at least in 6 of 8 rats) were observed in: systolic area (c), dP/dt_max_ (d), diastolic BP (j), pulse BP (k), dP/dt_d_ − dP/dt_max_ (q) and AnN − 1Max (rr).

### 3.4. Comparison of Non-Hysteresis/Hysteresis Relationships of Na_2_S with Published Relationships of GSNO

**Figure 4:**
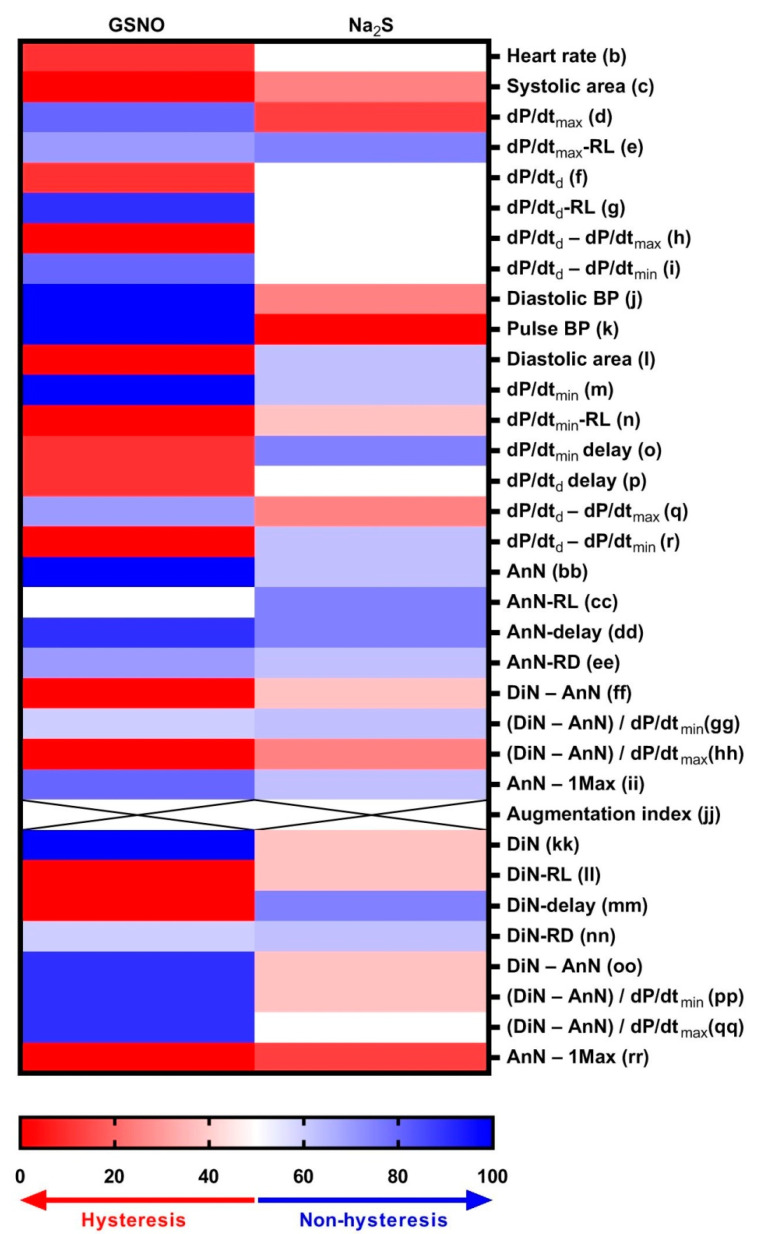
Comparison of the non-hysteresis (blue) or hysteresis (red) “patterns” between HPs to systolic BP after 10 µmol kg^−1^ H_2_S (n = 8) and 32 nmol kg^−1^ GSNO (n = 10, data from Figure 3 in Reference [4]) administration. Data are expressed as the percent of rats showing the non-hysteresis relationship.

In our previous study, we evaluated the non-hysteresis/hysteresis relationships between HPs to systolic BP after GSNO administration [4]. Here, we compared the trends of the non-hysteresis/hysteresis “patterns” of Na_2_S and GSNO (Figure 4). The comparison revealed that the “patterns” of 14 HPs were similar for both compounds. The following HPs showed opposite “patterns”: dP/dt_max_ (d), diastolic BP (j), pulse BP (k), diastolic area (l), dP/dt_min_ delay (o), dP/dt_d_ − dP/dt_max_ (q), dP/dt_d_ − dP/dt_min_ (r), DinN (kk), DiN delay (mm), DiN − AnN (oo), and (DiN − AnN)/dP/dt_min_ (pp)’. The other eight HPs showed different correlations, e.g., non-hysteresis/hysteresis vs. no “patterns” for heart rate (b), dP/dt_d_ (f), dP/dt_d_-RL (g), dP/dt_d_ − dP/dt_max_ (h), dP/dt_d_ − dP/dt_min_ (i), dP/dt_d_ delay (p), AnN-RL (cc), and (DiN − AnN)/dP/dt_max_ (qq). The non-hysteresis/hysteresis was not possible to evaluate in the case of augmentation index for both compounds (jj).

### 3.5. Effect of H_2_S on Distinct Fluctuation of Diastolic BP

**Figure 5:**
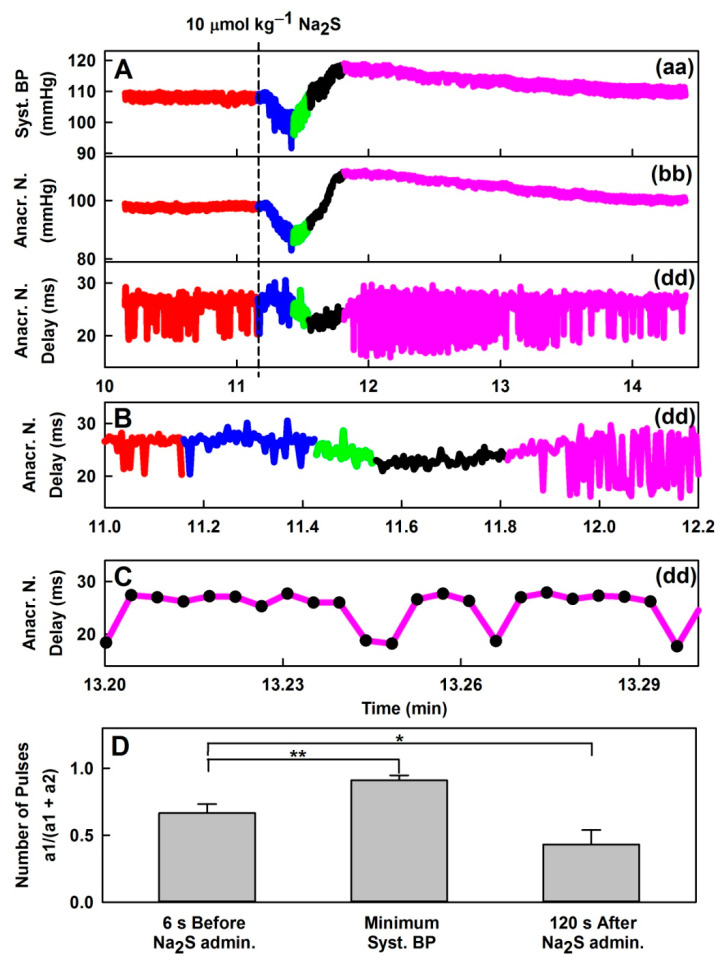
Representative non-filtered time-dependent effect of 10 µmol kg^−1^ Na_2_S on three HPs. (**A**) systolic BP (Syst. BP, mmHg) (aa), AnN (Anacr. N., mmHg) (bb) and AnN delay (Anacr. N. delay, ms) (dd); (the 10–15 min period is shown). (**B**) The fluctuation of AnN delay (dd); (11–12 min) reflects the time interval fluctuation between a1 and a2 points (Figure S1B). The higher value of AnN delay in ms indicates that diastolic BP at point a1 was lower than at point a2. (**C**) The fluctuation between a1 and a2 points in pulses (black circles) recorded at high resolution at 120 s after the 10 µmol kg^−1^ Na_2_S administration. Data were taken from Figure S8. (**D**) Comparison of the ratio of a1/(a1 + a2) pulses in 6 s long interval: Starting 6 s before the 10 µmol kg^−1^ Na_2_S administration; from 3 s before to 3 s after the minimum of systolic BP, and 120 s after the 10 µmol kg^−1^ Na_2_S administration. Data represent means ± SEM. Statistically significant differences between group means were determined by one-way repeated-measures ANOVA (*p* < 0.0001) with Dunnett’s post-hoc test (*p < 0.05, **p < 0.01, n = 8). For the explanation of (aa), (bb) and (dd) plots see the Description of the Table 1 and Figure S1.

The bolus administration of H_2_S also influenced a minor fluctuation of diastolic BP (Figure 5 and Figures S17–S23) that is clearly seen from the measurement of the time interval between AnN (point d in Figure S1A) and diastolic BP (point a1 or a2 in Figure S1B). This time interval (d–a1) or (d–a2) can be depicted as the time-dependent plot of AnN delay (Figure 5A, plot (dd)) and Figure 5B,C at a higher time resolution. The higher value of AnN delay in ms (Figure 5A(dd),B,C) indicates that diastolic BP (in mmHg) at point a1 was lower than at point a2. The time dependence of AnN delay more or less reflected the time-dependent changes of systolic BP (Figure 5A, plot (aa)) and the position of AnN in mmHg (Figure 5A, plot (bb)). The comparison of the fluctuation between a1 and a2 values at single pulses, expressed as the ratio of a1/(a1 + a2) pulses, increased ~5–12 s after the H_2_S administration, when systolic BP reached the minimum and decreased after 120 s (Figure 5D).

### 3.6. Comparison of Na_2_S, GSNO and SSNO^−^-Mix Effects on Rat BP

**Figure 6:**
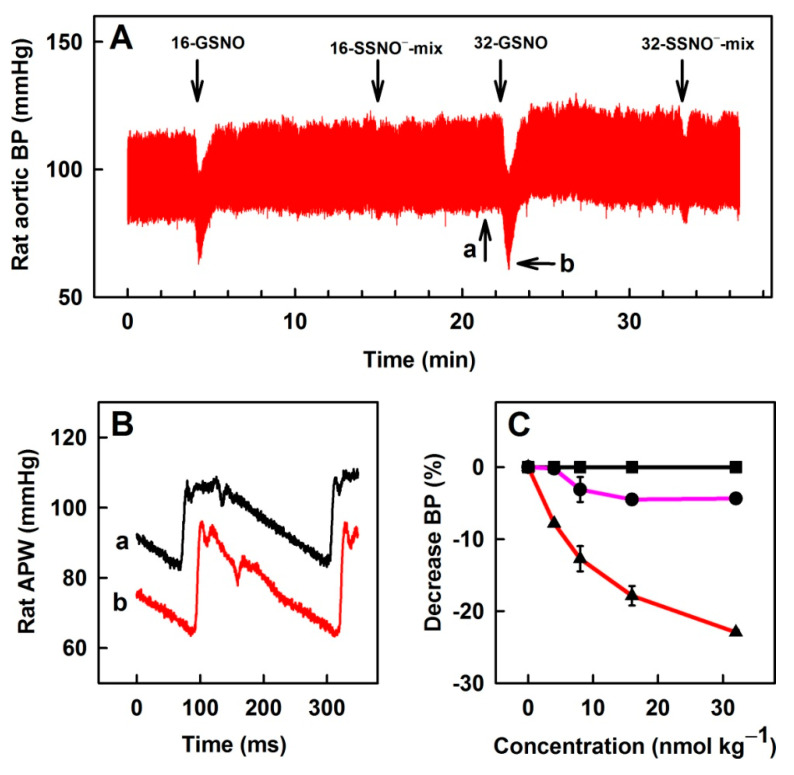
Effects of i.v. bolus administration of 16 and 32 nmol kg^−1^ GSNO (16- and 32-GSNO), 16 and 32 nmol kg^−1^ SSNO^−^-mix on BP in anesthetized rat (**A**) and the representative rat arterial pulse waveforms (**B**) before (a) and after (b) the administration of 32 nmol kg^−1^ GSNO. Comparison of the concentration-dependent effects of GSNO (red), SSNO^−^-mix (pink) and Na_2_S (black) on BP (**C**); n = 5 rats; number of administrations for each concentration ≥2. Data represent means ± SEM.

It is known that GSNO and H_2_S influence BP and interact together to form a mixture of several bioactive products. Nitrosopersulfide (SSNO^−^) is one of the major products of this interaction. Therefore, we termed the mixture as the SSNO^−^-mix. Here, we compared the effects of Na_2_S, GSNO and SSNO^−^-mix on rat BP. Bolus administration of 40, 80, 160 and 320 nmol kg^−1^ Na_2_S did not affect BP, whereas the administration of 4, 8, 16 and 32 nmol kg^−1^ of NO-donor GSNO transiently decreased BP in a concentration-dependent manner (Figure 6). SSNO^−^-mix applied at a 10:1 molar ratio (by dilution of stock solution prepared by the reaction of 10 mmol L^−1^ Na_2_S with 1 mmol L^−1^ GSNO to final bolus administrations of Na_2_S:GSNO at a ratio of 40:4; 80:8; 160:16; and 320:32 in nmol kg^−1^) had a minor effect on BP.

**Figure 7:**
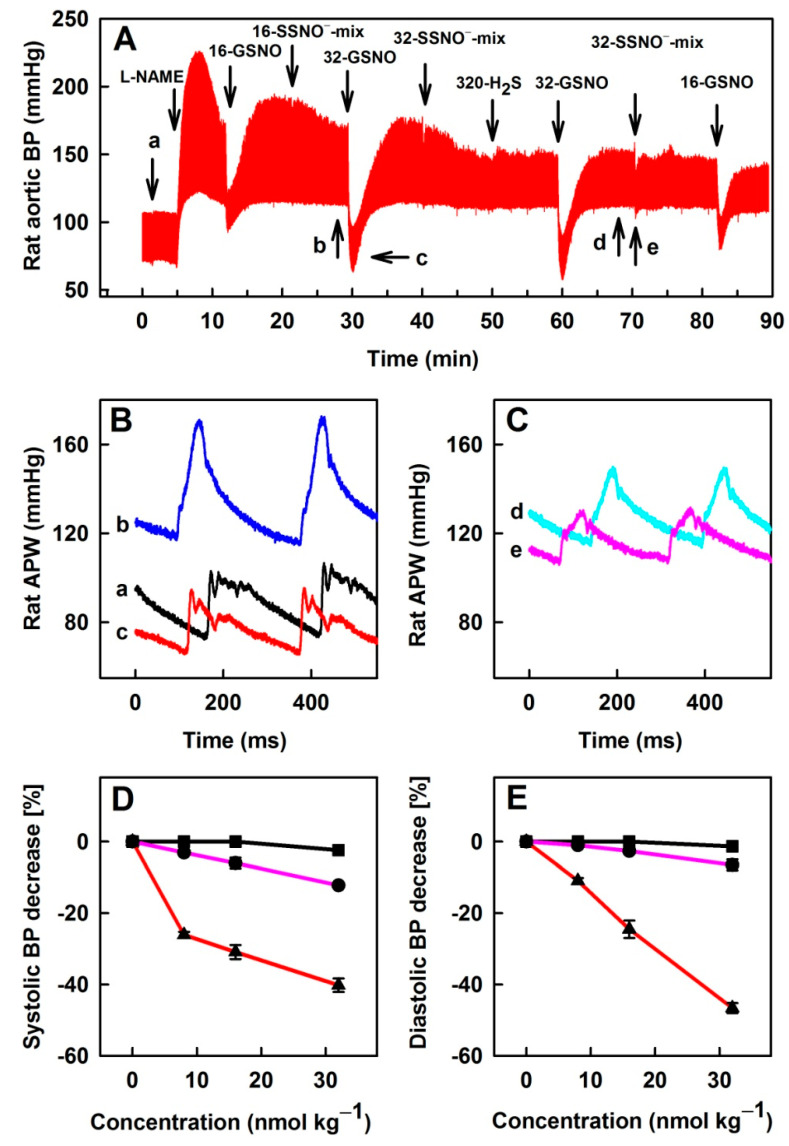
Effects of i.v. bolus administration of 16 and 32 nmol kg^−1^ GSNO (16- and 32-GSNO), 16 and 32 nmol kg^−1^ SSNO^−^-mix and 320 nmol kg^−1^ Na_2_S (320-H_2_S) on BP in anesthetized rat in the presence of 25 mg kg^−1^ L-NAME (**A**) and the representative rat arterial pulse waveforms (**B**,**C**) before the administration of L-NAME (a), before (b) and after (c) the administration of 32 nmol kg^−1^ GSNO in the presence of L-NAME; and before (d) and after (e) the administration of 32 nmol kg^−1^ SSNO^−^-mix in the presence of L-NAME. Comparison of the concentration-dependent effect of GSNO (red), SSNO^−^-mix (pink) and Na_2_S (black) on systolic (**D**) and diastolic BP (**E**); n = 4 rats; number of administrations for each concentration ≥2. Data represent means ± SEM.

The comparison of Na_2_S, GSNO and SSNO^−^-mix effects on rat BP was also studied in the condition of a decreased NO bioavailability. It has been shown that L-NAME, an inhibitor of nitric oxide synthase (NOS), decreased NO bioavailability and significantly influenced HPs [6]. Particularly, L-NAME (25 mg kg^−1^) significantly increased BP and influenced shape of APW (Figure 7). Bolus administration of Na_2_S at concentrations 40–320 nmol kg^−1^ did not significantly affect BP. However, GSNO at the concentrations 4–32 nmol kg^−1^ transiently and pronouncedly decreased BP in a concentration-dependent manner in the presence of L-NAME (Figure 7). However, SSNO^−^-mix (Na_2_S:GSNO; 80:8; 160:16; and 320:32 in nmol kg^−1^) had only a minor effect on BP.

## 4. Discussion

This work is a continuation of studies based on the hypothesis that it is possible to significantly characterize the cardiovascular system in physiological and patho-physiological conditions just from the shape of APW [4,5,6]. To characterize the cardiovascular system from APW, the connections between “unique patterns” of APW parameters and specific physiological and patho-physiological conditions must be known. Effects of drugs on APW, for example specific inhibitors/activators of ion channels or regulation enzymes, can help to find these “patterns” of APW parameters specific for particular patho-physiological conditions of the cardiovascular system. Our previous studies indicate that the recording system used in our work detects subtle changes of APW with sufficient reproducibility under equal experimental conditions [4]. Using this approach, we characterized time-dependent changes of APW caused by i.v. administration of H_2_S, and compared these changes with the effects of NO-donor GSNO and with the effects of H_2_S/GSNO interaction mixture (SSNO^−^-mix). It is noticed that the results were obtained in rats under anesthesia.

Reproducibility of the effects of H_2_S on HPs, their cross-relationships and non-hysteresis/hysteresis “patterns” were significantly lower than the reproducibility of the effects for the NO-synthase inhibitor L-NAME and NO-donor GSNO [4,5,6]. It might result from numerous interactions of H_2_S with different components of the cardiovascular system, which were reported previously [21]. Additionally, the interaction of H_2_S with biological disulfides (RSSR) results in the formation of equilibrium of various sulfur species, e.g., H_2_S, RSH, RSSR, RSSH and RSSSR, which may be responsible for its diverse activity [22]. These may contribute to the explanation of dissimilar effects of H_2_S on single HPs, as discussed below.

From the time-dependent H_2_S effects, it is evident that most of the 34 HPs did not follow the time-dependent changes of systolic BP and that nearly each HP showed a different time-dependent response to H_2_S (Figure 1 and Figures S2–S8). These results indicate that H_2_S influences each of the 35 HPs differently, confirming specific “patterns” of responses included in the shape of APW. H_2_S transiently decreased systolic BP similarly to GSNO, but 10 out of 35 HPs showed diverse trends for H_2_S and GSNO, indicating different “patterns” of their effects on hemodynamics, based on different signaling pathways.

In order to look for more detailed “patterns” of effects of H_2_S on the cardiovascular system, the time-dependent changes between HPs, described as their cross-relationships, were evaluated. Each of the cross-relationship between 34 HPs and the systolic BP (Figure 2 and Figures S9–S15) showed a specific “pattern”, indicating that various signaling pathways influence the shape of APW after the administration of H_2_S. Some of the cross-relationships showed non-hysteresis or hysteresis loops (Figure 3). Similarly, as in our previous studies [4,5], it is suggested that the non-hysteresis of the cross-relationship between two particular HPs indicates a direct connection between the signaling pathways regulating these two HPs, whereas hysteresis indicates that the signaling pathways are connected indirectly. The direct or indirect connections of signaling pathways regulating the connection between two HPs are also considered as a “pattern” for H_2_S action. However, more studies are needed to conclude which of the time-dependent and cross-relationships “patterns” are unique for the H_2_S action.

The comparison of the non-hysteresis or hysteresis cross-relationships of HPs between H_2_S (Figure 3) and GSNO (Figure 3C in [4]) showed similar, but also opposite “patterns” for particular HPs (Figure 4). Therefore, we may suggest that some of the HPs are regulated by the activation of the same pathways for both compounds, however others were modulated by different mechanisms.

NO and H_2_S coupled signaling pathways in the cardiovascular system are complex and not fully understood [9,21,23,24,25,26,27,28]. The involvement of NO in the regulation of BP is associated with the modulation of activity of various ion channels. For instance, NO signaling leads to an increased activity of large conductance Ca^2+^-dependent K^+^ channels (BK_Ca_), resulting in K^+^ efflux, causing hyperpolarization of cell membrane and consequent deactivation of L-Type Ca^2+^ channels. In addition, NO may decrease intracellular Ca^2+^ levels by direct inhibition of L-Type Ca^2+^ channels, thus reducing Ca^2+^ influx, leading to smooth muscle relaxation [21,23,24,25,26]. Similarly, the effects of H_2_S on BP are mediated through the modulation of the activity of membrane channels. Several reports show that the H_2_S induced vasorelaxation is caused by the activation of potassium channels, particularly ATP-sensitive potassium channels (K_ATP_) or small to medium conductance K_Ca_ channels [21,23,29]. Furthermore, vascular effects of NO and H_2_S are mediated through cyclic guanosine monophosphate (cGMP) signaling. NO increases the cGMP levels by activating guanylyl cyclase and H_2_S inhibits phosphodiesterase activity that catalyzes the degradation of cGMP [30]. In turn, cGMP stimulates the activity of protein kinase G (PKG), leading to the dephosphorylation of myosin light chains (MLC) and smooth muscle relaxation. Alternatively, H_2_S activates PKG by oxidation and disulfide formation, independently of cGMP signaling [31].

The transient decrease of BP induced by H_2_S and NO may at least partially result from hyperpolarization, closing of voltage-dependent calcium channels and reduction of intracellular Ca^2+^ or reduction of MLC phosphorylation. We may suggest that the similar responses of the cross-relationships of HP_S_ to systolic BP for H_2_S and NO (Table 1) and resembling non-hysteresis/hysteresis “patterns” (Figure 4) reflect the “ion channel signaling pathways” or the “PKG signaling pathway” by both compounds. The different responses of the cross-relationships of HP_S_ to H_2_S and NO and dissimilar non-hysteresis/hysteresis “patterns” may reflect other pathways that are specific for single compounds. For instance, Nier et al. suggested that the changes in the relative levels of DiN are specific for NO signaling and negatively correlate with NO bioavailability [32]. Our study showed that H_2_S decreased the relative level of DiN, indicating that the NO-dependent signaling pathway was activated. Moreover, the relationships between DiN-RL (ll) and systolic BP showed mostly hysteresis “patterns” after GSNO and Na_2_S administration, implicating that the changes in DiN-RL (ll) are activated at different time points and probably by different signaling pathways than the changes in systolic BP (Figure 4). While having a similar effect on BP, GSNO and H_2_S affected HPs, which reflect heart functions differently. GSNO had a negative inotropic (dP/dt_max_-RL) and lusitropic (dP/dt_min_-RL) effect, however H_2_S had a minor effect on heart contraction and relaxation. At the same time, H_2_S decreased heart rate, but GSNO did not affect the heart chronotropy. This is in line with a negative inotropic effect of H_2_S mediated through the opening of K_ATP_ channels and no effect on heart contractile function reported by others [33,34]. For the depressive actions of NO on contraction and relaxation, desensitization of cardiac myofilaments through the cGMP/PKG pathway was described [35,36]. Interestingly, the relationships between dP/dt_max_-RL (e) and BP showed mostly non-hysteresis “patterns”, however the dP/dt_min_-RL (n) to BP showed mostly hysteresis “patterns” for both compounds (Figure 4). Therefore, we suppose that the effects on heart contraction may be mediated by the same signaling pathways as the effects of the compounds on BP. However, the changes in heart relaxation are postponed in comparison to BP changes and probably mediated by different signaling pathways.

Several reports propose that the products of H_2_S-NO interaction, particularly SSNO^−^, play an important role in the regulation of BP [9,17,18,37]. Therefore, we have studied the effect of SSNO^−^-mix on BP. SSNO^−^-mix in the presence or absence of L-NAME had minor effects on BP in comparison to GSNO (Figure 6 and Figure 7). Effects of bolus i.v. administration of SSNO^−^-mix on BP have been previously studied at concentrations ≥30 nmol kg^−1^ and decreased BP in a concentration-dependent manner [12,14]. The observed minor effects of i.v. administration of SSNO^−^-mix at low concentrations (Figure 6 and Figure 7) with comparison to GSNO on HPs is in contrast to its published potency to relax isolated aortic rings ex-vivo, in which the effects of SSNO^−^-mix was several times more potent and faster than GSNO [37]. It has been suggested that NO and HNO, among other species formed in the SSNO^−^-mix, were involved in the vessels relaxation [14,37]. However, in blood a fast inactivation of NO and HNO occurs after the interaction with hemoglobin [38,39]. While the membrane of red blood cells is permeable to NO, the consumption of NO by red blood cells is reduced by an intrinsic barrier and an extracellular unstirred layer of solvent [40,41]. Our data suggest that the products of the SSNO^−^-mix are decomposed or metabolized faster in the blood than GSNO. From this, it is suggested that, at low concentrations (4–32 nmol kg^−1^), the SSNO^−^-mix and/or the products of SSNO^−^-mix decomposition are almost all quenched by blood components before reaching the surface of vascular endothelial cells, leading to minor effects on BP. We may speculate that the mechanisms limiting the consumption of NO by red blood cells are less effective in the case of the products formed in the SSNO^−^-mix due to different physical and chemical properties of these species. While in the case of isolated aortic rings, SSNO^−^-mix and its decomposition products directly interact with the membrane and/or intracellular targets [37]. These results suggest that the formation of SSNO^−^-mix in blood, but not in the cells, diminishes the effects of NO on BP.

As discussed in our previous studies [4,6], the physiological origin of anacrotic notch (point d in Figure S1A) and the minor time/pressure fluctuation of diastolic BP (Figure S1A,B, point a–a1 or a2) are not yet fully understood. Effect of H_2_S on the distinct fluctuation of a1 or a2 (Figure 5) was similar (but not identical) to the effect reported for GSNO [4]. The observations that the points fluctuated between two distinct positions and that the fluctuation was influenced by H_2_S and GSNO during decreasing/increasing of BP indicate that it might be a physiological, yet unknown, important cardiovascular phenomenon.

## 5. Conclusions

Our work characterizes novel details of the cardiovascular responses and presents numerous original data characterizing APW by 35 HPs and by their cross-relationships after i.v. administration of 10 µmol kg^−1^ H_2_S. Since the effect of H_2_S on BP was transient, we showed non-hysteresis/hysteresis time-dependent changes of 35 HPs, and from their cross-relationships to systolic BP, direct/indirect signaling pathways of the H_2_S effects were proposed. We assume that the similar responses of HP_S_ to H_2_S and NO, their resembling cross-relationships and non-hysteresis/hysteresis “patterns” reflect the activation of “channels or PKG signaling pathways” by both compounds. In addition, the H_2_S/GSNO product (SSNO^−^-mix) at a concentration ≤32 nmol kg^−1^ showed a minor effect on hemodynamics in comparison to GSNO, suggesting that the formation of SSNO^−^-mix in blood diminished the effect of NO on BP. The observed time-dependent changes of 35 HPs, their cross and non-hysteresis/hysteresis relationships may serve as “patterns” for the conditions of a transient decrease/increase of BP caused by H_2_S.

In summary, the analysis of the hemodynamic effects of H_2_S is a starting point for the characterization of the cardiovascular system by 35 HPs under physiological conditions. However, further validation of the results in animal models of diseases will be useful and represents a challenge for future studies.

## Figures and Tables

**Table 1:** Comparison of the time-dependent transient effects of 10 µmol kg^−1^ H_2_S (at least 6 out of 8 rats) on 35 HPs (red lines in Figure 1 and Figures S2–S8) and 32 nmol kg^−1^ GSNO (data from Figure 6, Table 1 in Reference [4]).

Description		GSNO	Na_2_S
(a)	Systolic blood pressure; in mmHg	↓	↓
(b)	Heart rate; in min^−1^	~	↓
(c)	Systolic area; in mmHg s	↑	↑
(d)	dP/dt_max_; in mmHg ms^−1^	↑	~
(e)	dP/dt_max_ relative level	↓	↓
(f)	dP/dt_d_; in mmHg ms^−1^	↓↑	~
(g)	dP/dt_d_ relative level	↓	↓
(h)	dP/dt_d_ − dP/dt_max_; in s	↑	↑
(i)	dP/dt_d_ − dP/dt_min_; in s	↑↓	↑
(j)	Diastolic blood pressure; in mmHg	↓	↓
(k)	Pulse blood pressure; in mmHg	↑	↑
(l)	Diastolic area; in mmHg s	↓	↑
(m)	dP/dt_min_; in mmHg ms^−1^	↓	↓
(n)	dP/dt_min_ relative level	↓	~
(o)	dP/dt_min_ delay; in s	↑	↑
(p)	dP/dt_d_ delay; in s	↑	↑
(q)	dP/dt_d_ − dP/dt_max_; in mmHg	↓	↓
(r)	dP/dt_d_ − dP/dt_min_; in mmHg	↑	~
(aa)	Systolic blood pressure; in mmHg	↓	↓
(bb)	Anacrotic notch; in mmHg	↓	↓
(cc)	Anacrotic notch relative level	↑	↑
(dd)	Anacrotic notch delay; in ms	↑	↑
(ee)	Anacrotic notch relative Delay	↑	~
(ff)	Dicrotic notch (DiN) − Anacrotic notch (AnN); in s	↑	↑
(gg)	(DiN − AnN)/dP/dt_min_; in s/mmHg µs^−1^	↑	↑
(hh)	(DiN − AnN)/dP/dt_max_; in s/mmHg µs^−1^	↓	~
(ii)	AnN − 1Max; in ms	↑	↑
(jj)	Augmentation index relative	~	~
(kk)	Dicrotic notch; in mmHg	↓	↓
(ll)	Dicrotic notch relative level	↓	↓
(mm)	Dicrotic notch delay; in ms	↑	↑
(nn)	Dicrotic notch relative delay	↑	~
(oo)	DiN − AnN; in mmHg	↓	↓
(pp)	(DiN − AnN)/dP/dt_min_; in mmHg/mmHg ms^−1^	↑	↑
(qq)	(DiN − AnN)/dP/dt_max_; in mmHg/mmHg ms^−1^	↓	↓
(rr)	AnN − 1Max; in mmHg	~	~

## Data Availability

All findings and conclusions are based on the presented figures in the main text or in the supplementary information. Original source files can be sent from the corresponding author, Karol Ondrias, upon request.

## Supplementary Materials

The following are available online at https://www.mdpi.com/2218-273X/11/2/293/s1, Figures S1–S23. Figure S1: The left common carotid artery pulse waveform (APW) in the anesthetized rat, Figures S2–S8: Time-dependent changes of HPs of anesthetized rat after i.v. bolus administration of 10 µmol kg^–1^ Na_2_S measured in 7 independent experiments, Figures S9–S15: Relationships of HPs to the systolic BP after administration of 10 µmol kg^–1^ Na_2_S obtained in 7 independent experiments, Figure S16: Number of rats representing the non-hysteresis/hysteresis patterns of the relationships of HPs to systolic BP after administrations of 10 µmol kg^–1^, Figures S17–S23: Time-dependent effect of 10 µmol kg^–1^ Na_2_S on systolic BP (mmHg), AnN (mmHg) and AnN delay (ms) measured in 7 independent experiments.
