# Supplementary material for: Cardiovascular “Patterns” of H2S and SSNO−-Mix Evaluated from 35 Rat Hemodynamic Parameters
Source: Biomolecules. 2021 Feb 16;11(2):293. doi: 10.3390/biom11020293 (PMC7920056; doi:10.3390/biom11020293)
Supplement: Supplementary file 1 [file biomolecules-11-00293-s001.pdf]

Supplementary Material

to

Cardiovascular ʺpatterns ʺ of H_2S and $SSNO^-$-mix evaluated from 35 rat hemodynamic parameters

Lenka Tomasova[1]*, Marian Grman[1]*, Anton Misak[1], Lucia Kurakova[2], Elena Ondriasova[2], Karol Ondrias[1]**

[1] Institute of Clinical and Translational Research, Biomedical Research Center, Slovak Academy of Sciences, Dubravska cesta 9, 845 05 Bratislava, Slovakia

[2] Department of Pharmacology and Toxicology, Faculty of Pharmacy, Comenius University, Odbojarov 10, 832 32 Bratislava, Slovakia

* L.T. and M.G. contributed equally to this work

** Correspondence: Karol Ondrias; e-mail: karol.ondrias@savba.sk; https://orcid.org/0000-0001-8329-3563

Description and abbreviation of 35 hemodynamic parameters (for details see Kurakova et al. [1] and Misak et al. [2]).

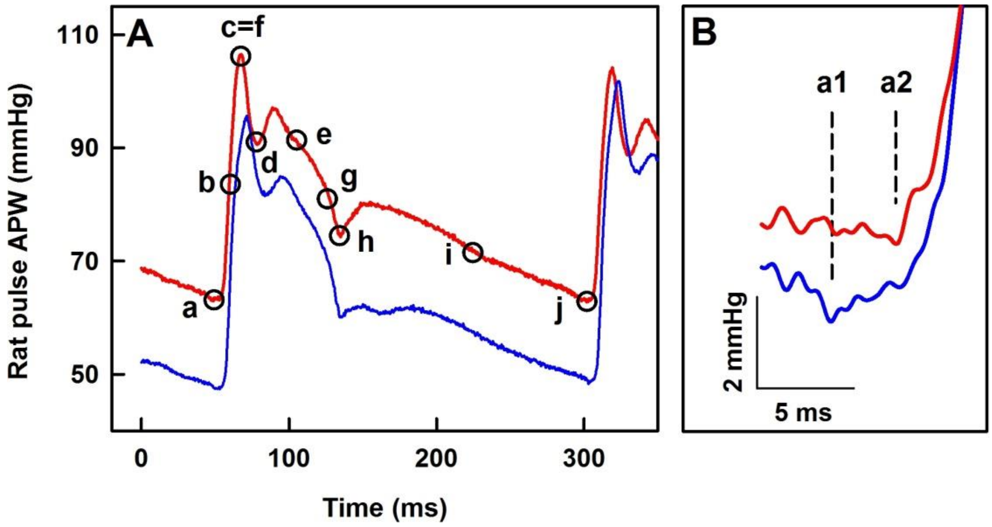

Figure S1. The left common carotid artery pulse waveform (APW) in the anesthetized rat. **A:** Control APW (red) with marked ten points **a** - **j** (black circles) before Na_2S administration. APW recorded 15 s after Na_2S (10 μmol kg^{-1}) i.v. administration (blue). **B:** Fluctuation of minimum diastolic BP, point **a** (**a1** or **a2**).

Ten points **a - j** (in bold letters) are from Figure S1A and they mark the values of BP and time that are used to define (calculate) specific hemodynamic parameters (HP).

(a) Systolic blood pressure in mmHg; point **c** or **f**.
(b) Heart rate in min^{-1}; 60 / (**j** – **a**); (**j** – **a**) represents time interval between **a** and **j**, **a** and **j** are two reference points to diastolic BP value.
(c) Systolic area in mmHg s; integral BP of **a** to **h**; **h** refers to BP at the dicrotic notch (dicrotic BP).
(d) dP/dt_{max} in mmHg ms^{-1}; maximum derivative at the point **b**; P is BP in mmHg.
(e) dP/dt_{max} relative level; relative level (shortly RL) of point **b**; (**b** – **a**) / (**c** (or **f**) – **a**) in mmHg/mmHg (dimensionless).
(f) dP/dt_{d} in mmHg ms^{-1}; negative maximum derivative at the point **i**; the point **i** is the BP at the middle of the time interval between **h** and **j**.
(g) dP/dt_{d} relative level, relative level of point **i**; (**i** – **a**) / (**c** (or **f**) – **a**) in mmHg/mmHg (dimensionless).
(h) dP/dt_{d} – dP/dt_{max} in s; time interval between **b** and **i**, dP/dt_{d} – dP/dt_{max} = (**i** – **b**).
(i) dP/dt_{d} – dP/dt_{min} in s; time interval between **g** and **i**, dP/dt_{d} – dP/dt_{min} = (**i** – **g**); dP/dt_{min} is negative maximum derivative at the point **g**.
(j) Diastolic blood pressure in mmHg; the point **a** or **j.**
(k) Pulse BP in mmHg; (**c** – **a**) or (**f** – **a**).
(l) Diastolic area in mmHg s; integral BP of **h** to **j**.
(m) dP/dt_{min} in mmHg ms^{-1}; dP/dt_{min} is maximum negative derivative at the point **g**.
(n) dP/dt_{min} relative level, relative level of point **g**; (**g** – **a**) / (**c** (or **f**) – **a**) in mmHg/mmHg (dimensionless).
(o) dP/dt_{min} delay in s; delay in s of point **g**; (**g** – **a**) time interval between **a** and **g**.
(p) dP/dt_{d} delay in s; delay in s of point **i**; (**i** – **a**) time interval between **a** and **i**.
(q) dP/dt_{d} – dP/dt_{max} in mmHg; (**i** – **b**) BP difference between **b** and **i**.
(r) dP/dt_{d} – dP/dt_{min} in mmHg; (**i** – **g**) BP difference between **g** and **i**.

(aa) Systolic blood pressure in mmHg; point **c** or **f**. Plot (aa) is the same as (a).
(bb) Anacrotic notch in mmHg; BP at the point **d**.
(cc) Anacrotic notch relative level; relative level of point **d**; (**d** – **a**) / (**c** (or **f**) – **a**) in mmHg/mmHg (dimensionless).
(dd) Anacrotic notch delay in ms; delay in ms of point **d**; (**d** – **a**) time interval between **a** and **d**.
(ee) Anacrotic notch relative delay; relative delay (shortly RD) of point **d**; (**d** – **a**) / (**j** – **a**) in ms/ms (dimensionless)
(ff) [Dicrotic notch (DiN) in s] – [Anacrotic notch (AnN) in s] in s; (**h** – **d**) time interval between **d** and **h**.
(gg) [(DiN – AnN) in s] / [dP/dt_{min} in mmHg μs^{-1}] in s/mmHg μs^{-1}; (**h** – **d**) / **g**.
(hh) [(DiN – AnN) in s] / [dP/dt_{max} in mmHg μs^{-1}] in s/mmHg μs^{-1}; (**h** – **d**) / **b**.
(ii) [AnN in ms] – [1Max (point **c** or the 1th. maximum) in ms] in ms; (**d** – **c**) time interval between **c** and **d**.
(jj) Augmentation index relative; (**f** – **c**) / (**f** – **a**) in mmHg/mmHg (dimensionless).
(kk) Dicrotic notch in mmHg; BP at the point **h**.
(ll) Dicrotic notch relative level; relative level of point **h**; (**h** – **a**) / (**c** (or **f**) – **a**) in mmHg/mmHg (dimensionless).
(mm) Dicrotic notch delay in ms, delay in ms of point **h**; (**h** – **a**).time interval between **a** and **h**.
(nn) Dicrotic notch relative delay; relative delay of point **h**; (**h** – **a**) / (**j** – **a**); in ms/ms (dimensionless)
(oo) [DiN in mmHg] – [AnN in mmHg] in mmHg; (**h** – **d**) BP difference between **d** and **h**;
(pp) [(DiN – AnN) in mmHg] / [dP/dt_{min} in mmHg ms^{-1}] in mmHg/mmHg ms^{-1}; (**h** – **d**) / **g**;
(qq) [(DiN – AnN) in mmHg] / [dP/dt_{max} in mmHg ms^{-1}] in mmHg/mmHg ms^{-1}; (**h** – **d**) / **b**.
(rr) [AnN in mmHg] – [1Max (point **c** or the 1th. maximum) in mmHg] in mmHg; (**d** – **c**) BP difference between **c** and **d**.

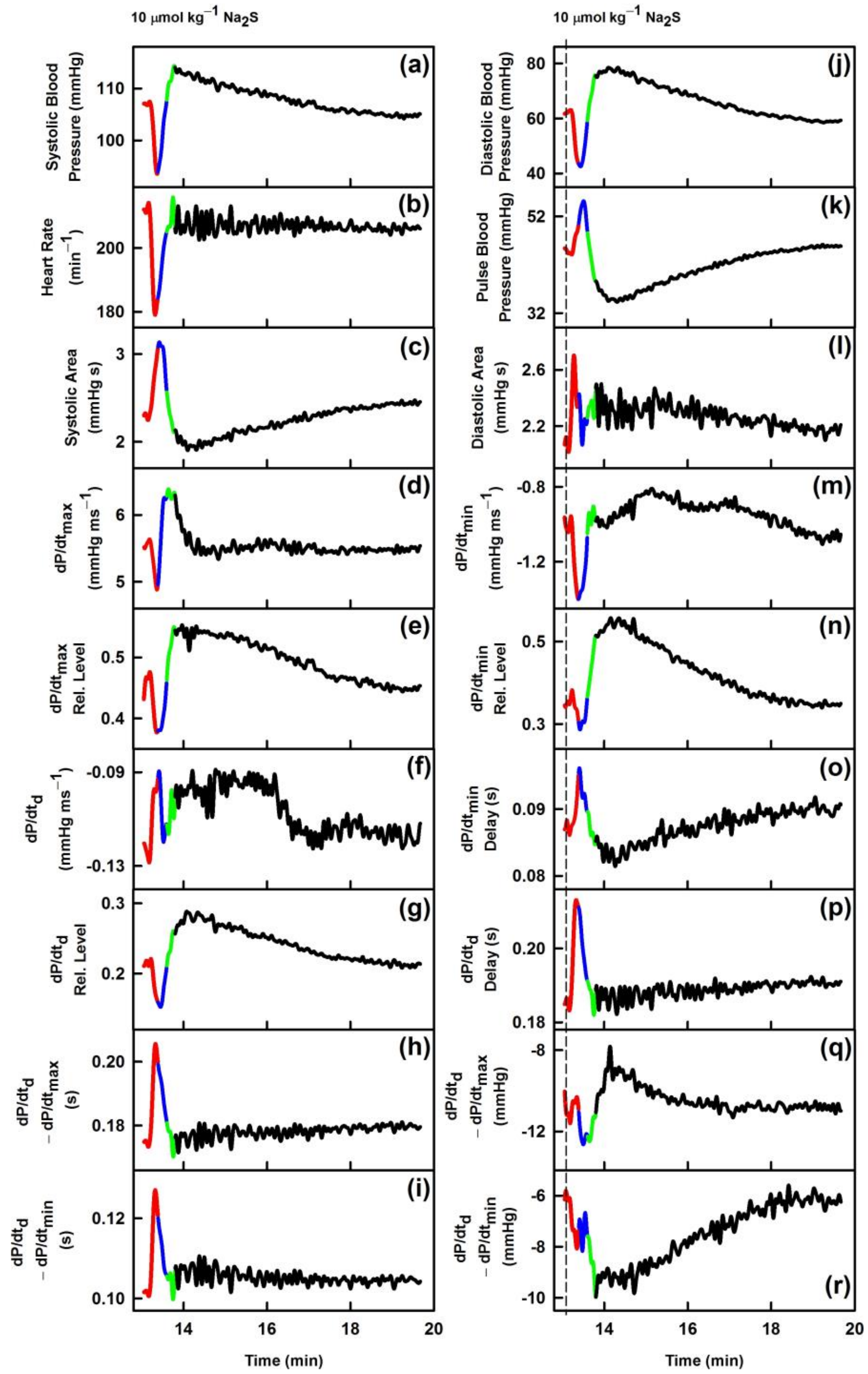

10 μmol kg−1 Na2S
10 μmol kg−1 Na2S
Systolic Blood Pressure (mmHg)
Heart Rate (min−1)
Systolic Area (mmHg s)
dP/dtmax (mmHg ms−1)
dP/dtmax Rel. Level
dP/dtd (mmHg ms−1)
dP/dtd Rel. Level
dP/dtd − dP/dtmax (s)
dP/dtd − dP/dtmin (s)
Diastolic Blood Pressure (mmHg)
Pulse Blood Pressure (mmHg)
Diastolic Area (mmHg s)
dP/dtmin (mmHg ms−1)
dP/dtmin Rel. Level
dP/dtmin Delay (s)
dP/dtd Delay (s)
dP/dtd − dP/dtmax (mmHg)
dP/dtd − dP/dtmin (mmHg)
(a)
(b)
(c)
(d)
(e)
(f)
(g)
(h)
(i)
(j)
(k)
(l)
(m)
(n)
(o)
(p)
(q)
(r)
Time (min)
Time (min)

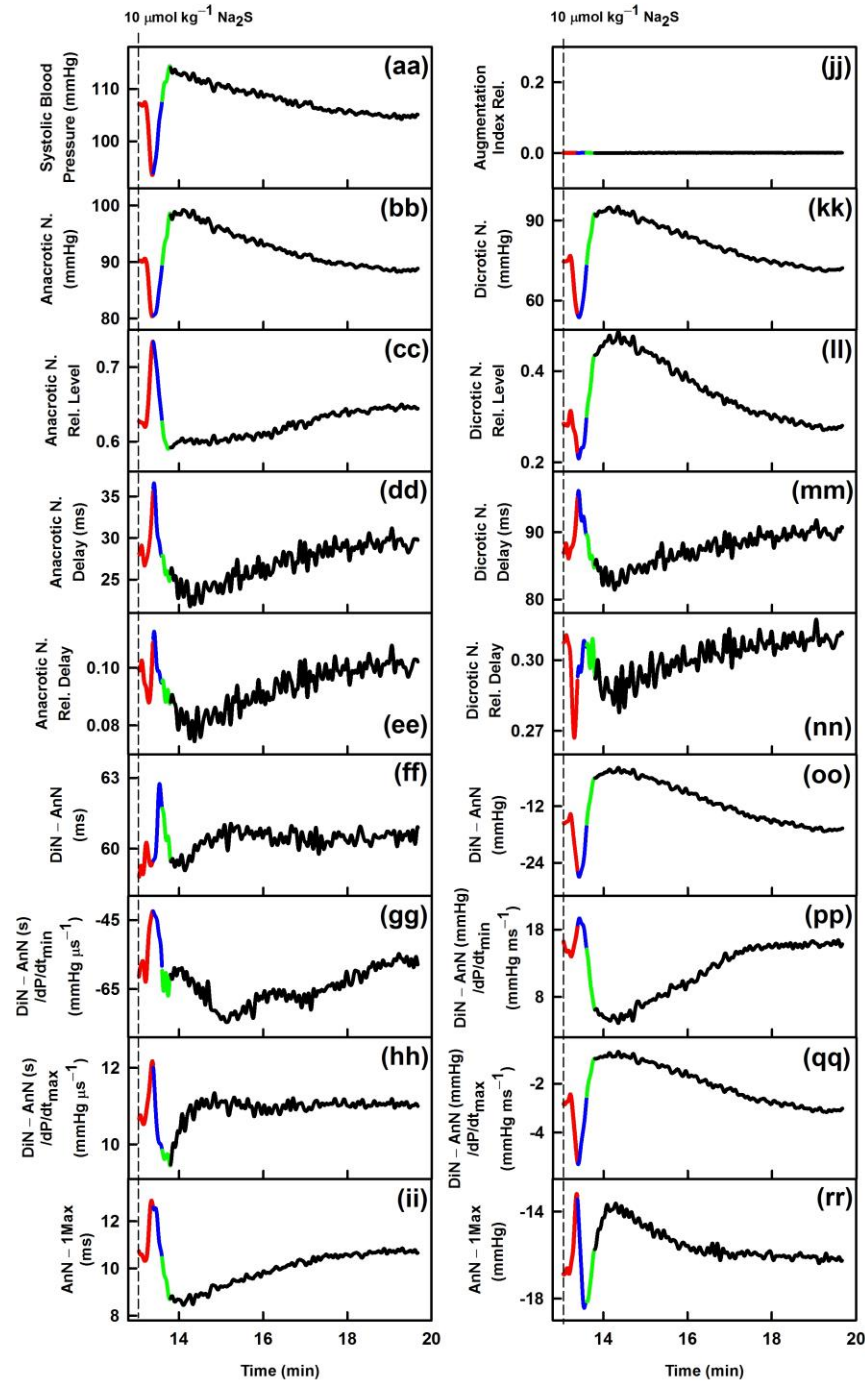

Figure S2. Time-dependent changes of HPs of anesthetized rat after I.V. bolus administration of 10 μmol kg−1 Na_2S (marked by dash lines). Colors: time period corresponds to the decrease of systolic BP (red), increase of systolic BP to the control value (blue), further increase of systolic BP to maximum (green) and decrease of systolic BP to the control value (black). The red line starts 3 s before Na_2S administration. For the definition of HPs, see the text below Figure S1.

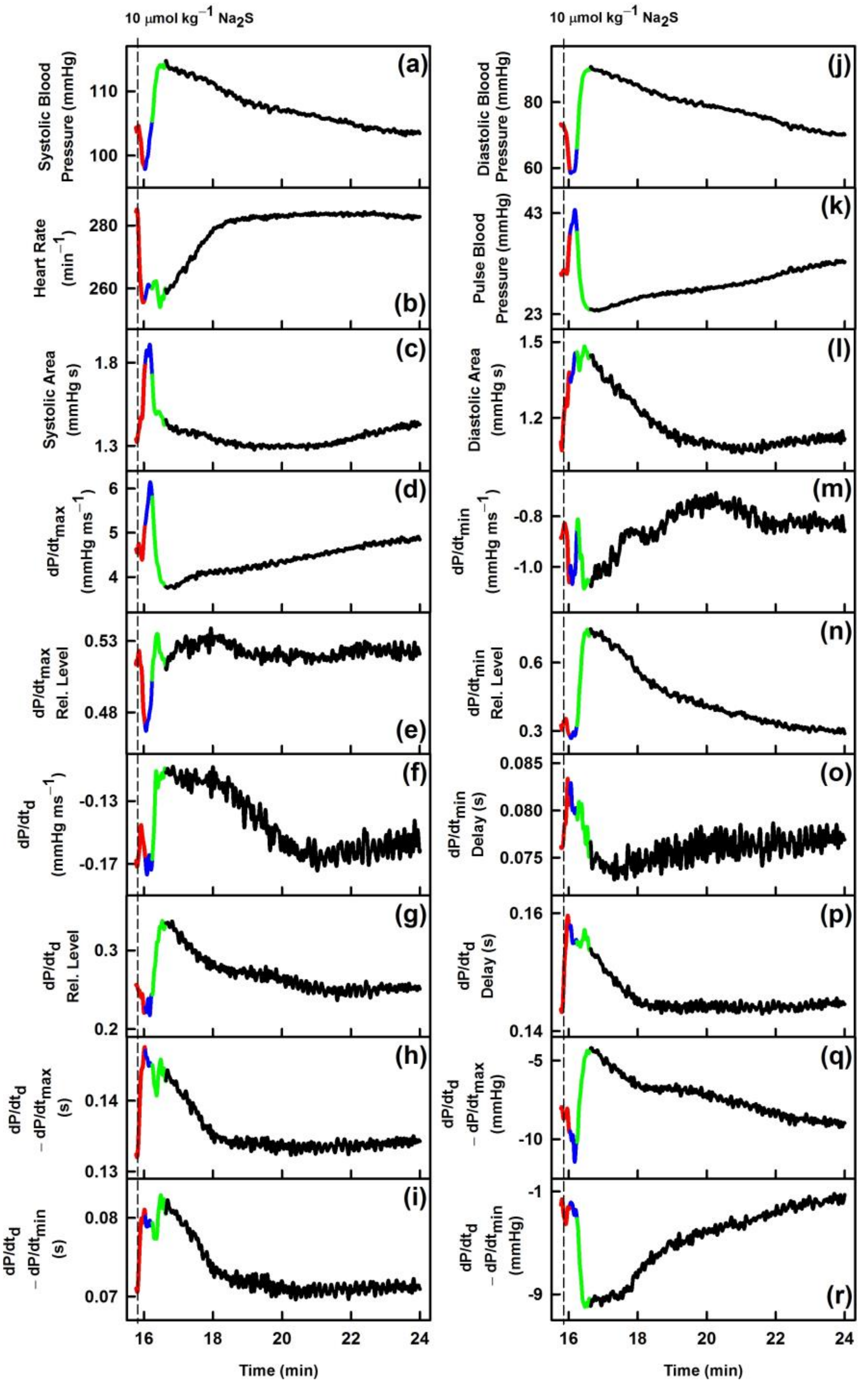
10 μmol kg−1 Na2S
10 μmol kg−1 Na2S
Systolic Blood Pressure (mmHg)
(a)
Heart Rate (min−1)
(b)
Systolic Area (mmHg s)
(c)
dP/dtmax (mmHg ms−1)
(d)
dP/dtmax Rel. Level
(e)
dP/dtd (mmHg ms−1)
(f)
dP/dtd Rel. Level
(g)
dP/dtd − dP/dtmax (s)
(h)
dP/dtd − dP/dtmin (s)
(i)
Diastolic Blood Pressure (mmHg)
(j)
Pulse Blood Pressure (mmHg)
(k)
Diastolic Area (mmHg s)
(l)
dP/dtmin (mmHg ms−1)
(m)
dP/dtmin Rel. Level
(n)
dP/dtmin Delay (s)
(o)
dP/dtd Delay (s)
(p)
dP/dtd − dP/dtmax (mmHg)
(q)
dP/dtd − dP/dtmin (mmHg)
(r)
Time (min)
Time (min)

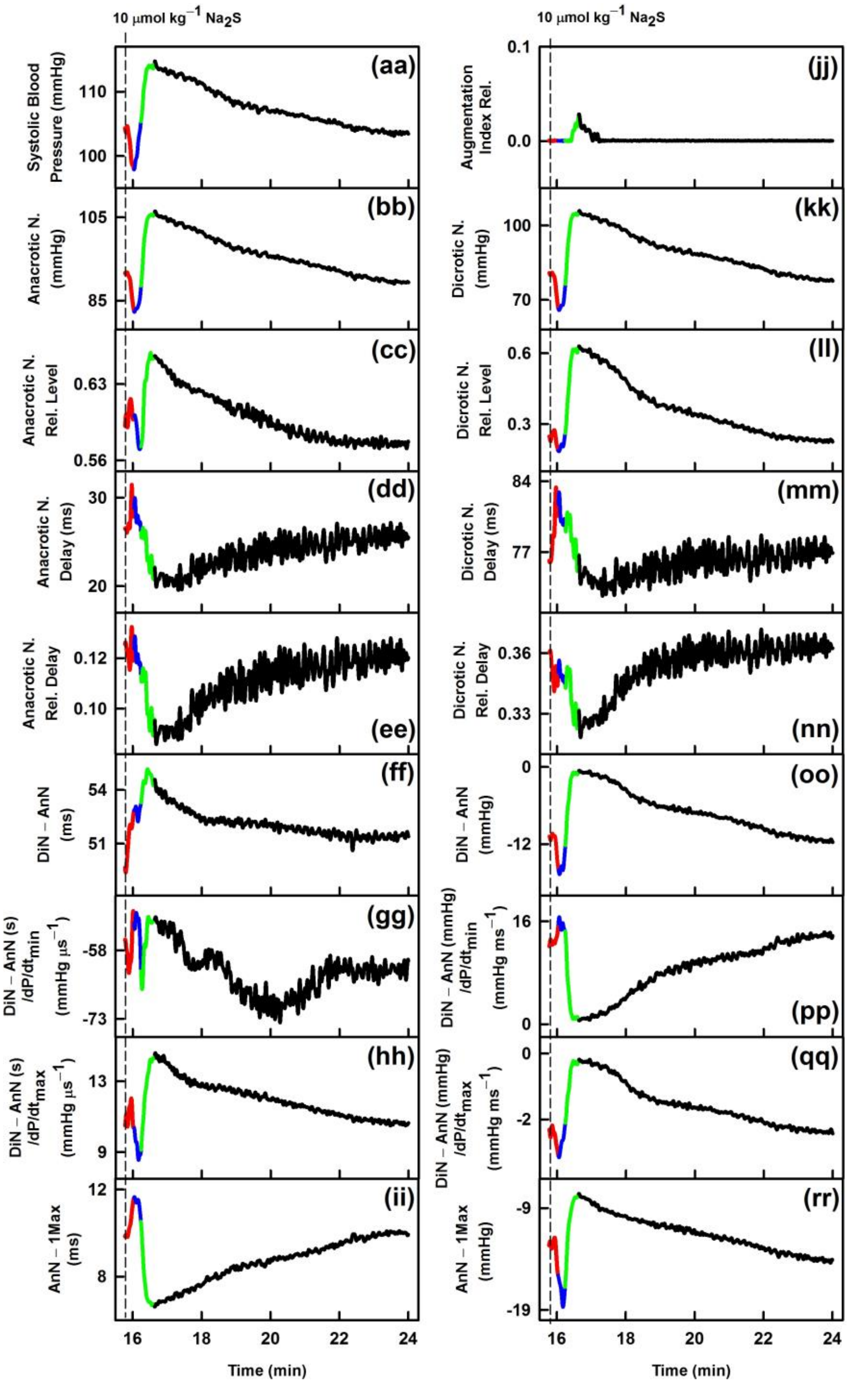

Figure S3. Time-dependent changes of HPs of anesthetized rat after I.V. bolus administration of 10 μmol kg^{-1} Na_2S (marked by dash lines). Colors: time period corresponds to the decrease of systolic BP (red), increase of systolic BP to the control value (blue), further increase of systolic BP to maximum (green) and decrease of systolic BP to the control value (black). The red line starts 3 s before Na_2S administration.

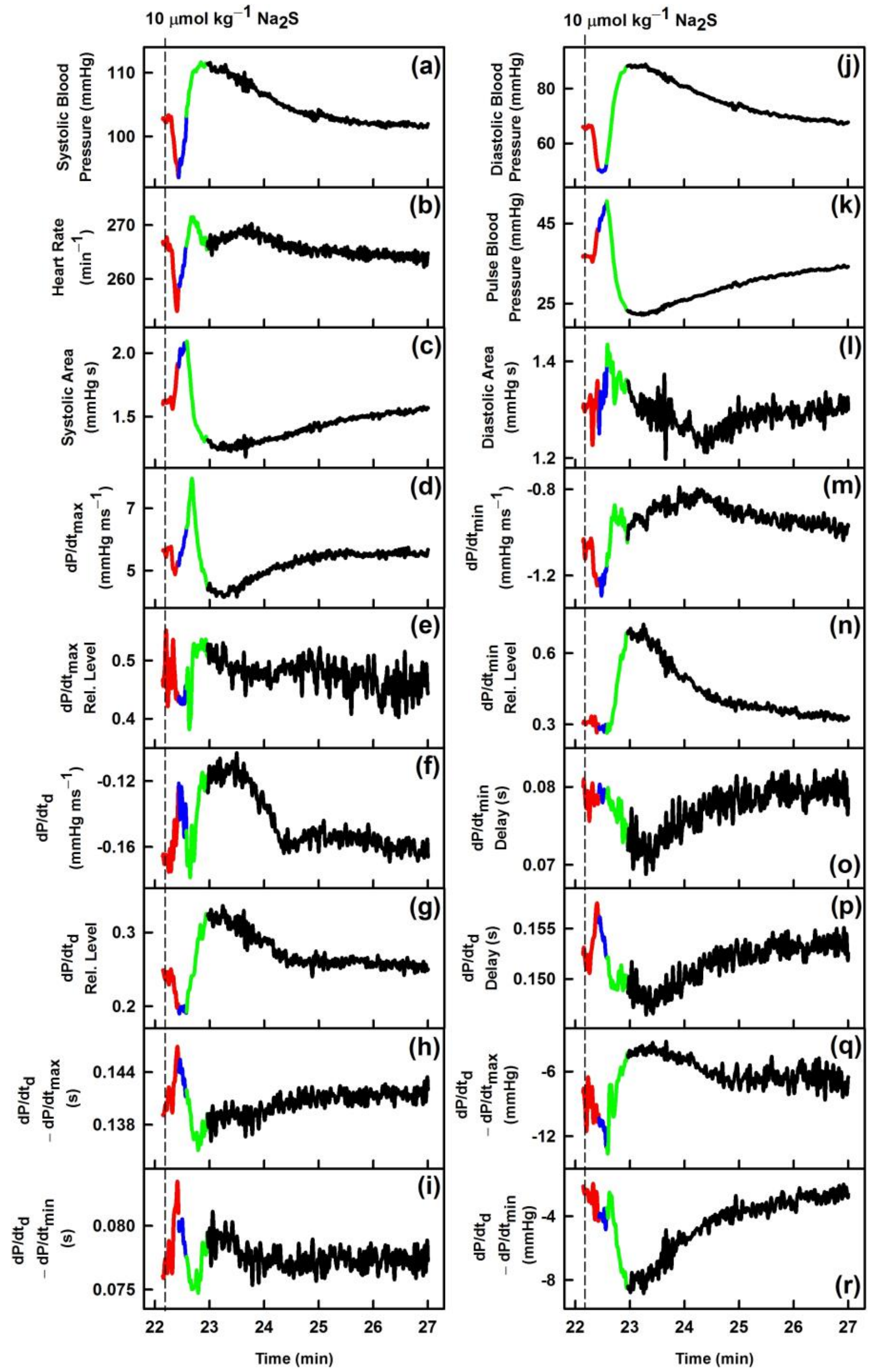

10 μmol kg⁻¹ Na2S
10 μmol kg⁻¹ Na2S
Systolic Blood Pressure (mmHg)
(a)
Heart Rate (min⁻¹)
(b)
Systolic Area (mmHg s)
(c)
dP/dtmax (mmHg ms⁻¹)
(d)
dP/dtmax Rel. Level
(e)
dP/dtd (mmHg ms⁻¹)
(f)
dP/dtd Rel. Level
(g)
dP/dtd − dP/dtmax (s)
(h)
dP/dtd − dP/dtmin (s)
(i)
Diastolic Blood Pressure (mmHg)
(j)
Pulse Blood Pressure (mmHg)
(k)
Diastolic Area (mmHg s)
(l)
dP/dtmin (mmHg ms⁻¹)
(m)
dP/dtmin Rel. Level
(n)
dP/dtmin Delay (s)
(o)
dP/dtd Delay (s)
(p)
dP/dtd − dP/dtmax (mmHg)
(q)
dP/dtd − dP/dtmin (mmHg)
(r)
Time (min)
Time (min)

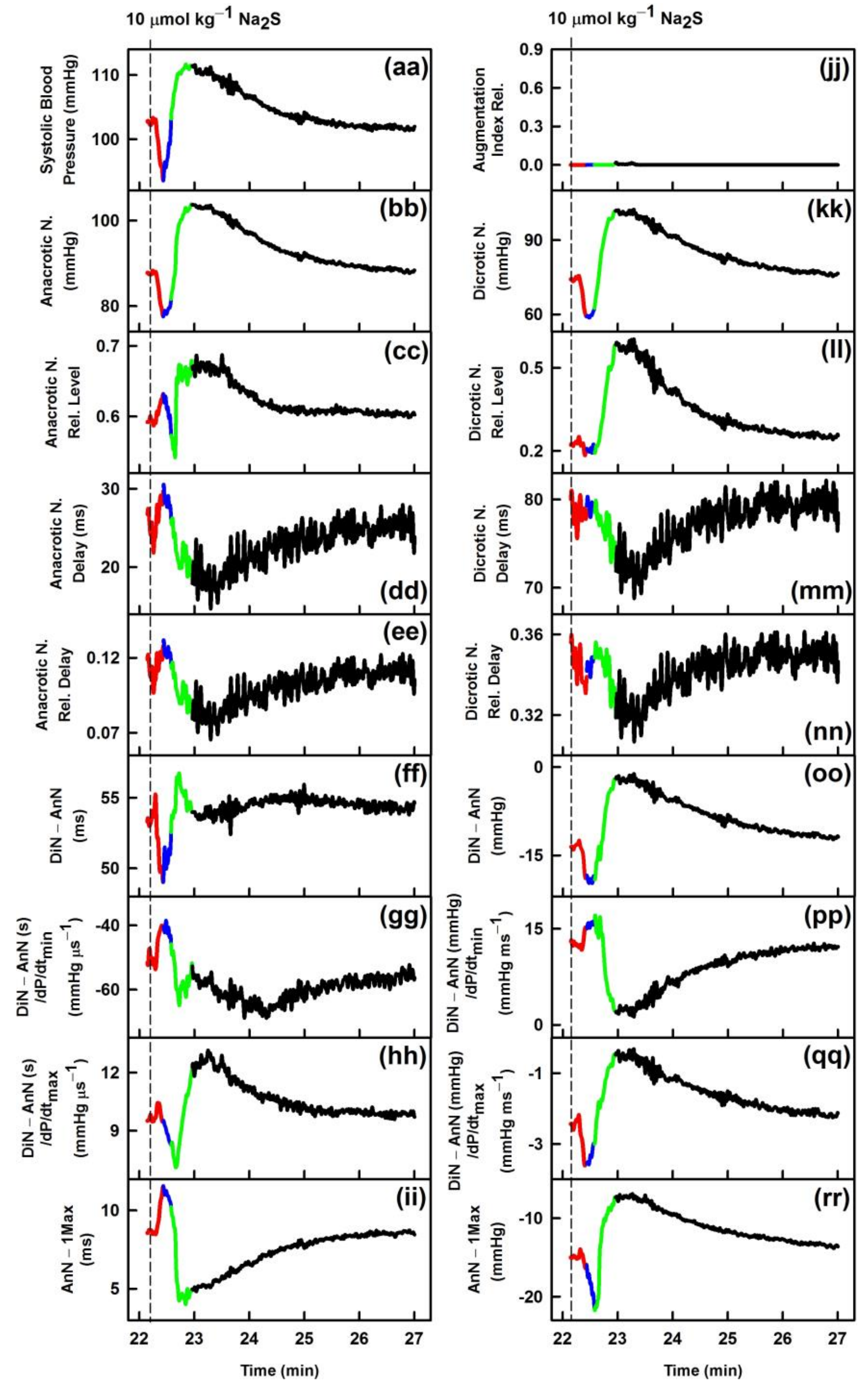

Figure S4. Time-dependent changes of HPs of anesthetized rat after I.V. bolus administration of 10 μmol kg⁻¹ Na_2S (marked by dash lines). Colors: time period corresponds to the decrease of systolic BP (red), increase of systolic BP to the control value (blue), further increase of systolic BP to maximum (green) and decrease of systolic BP to the control value (black). The red line starts 3 s before Na_2S administration.

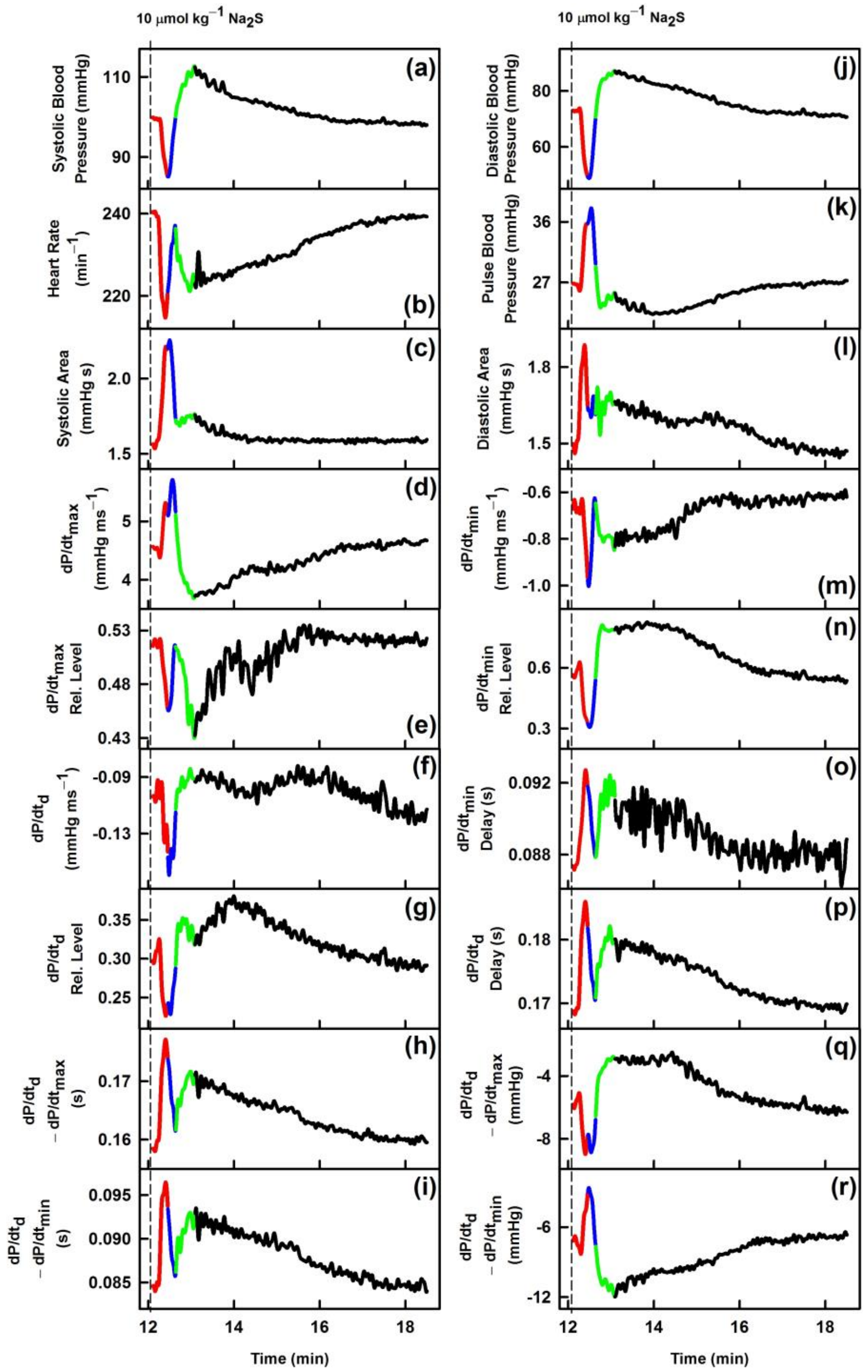

10 μmol kg−1 Na2S
Systolic Blood Pressure (mmHg)
Heart Rate (min−1)
Systolic Area (mmHg s)
dP/dtmax (mmHg ms−1)
dP/dtmax Rel. Level
dP/dtd (mmHg ms−1)
dP/dtd Rel. Level
dP/dtd − dP/dtmax (s)
dP/dtd − dP/dtmin (s)
Diastolic Blood Pressure (mmHg)
Pulse Blood Pressure (mmHg)
Diastolic Area (mmHg s)
dP/dtmin (mmHg ms−1)
dP/dtmin Rel. Level
dP/dtmin Delay (s)
dP/dtd Delay (s)
dP/dtd − dP/dtmax (mmHg)
dP/dtd − dP/dtmin (mmHg)
(a) (b) (c) (d) (e) (f) (g) (h) (i) (j) (k) (l) (m) (n) (o) (p) (q) (r)
Time (min)

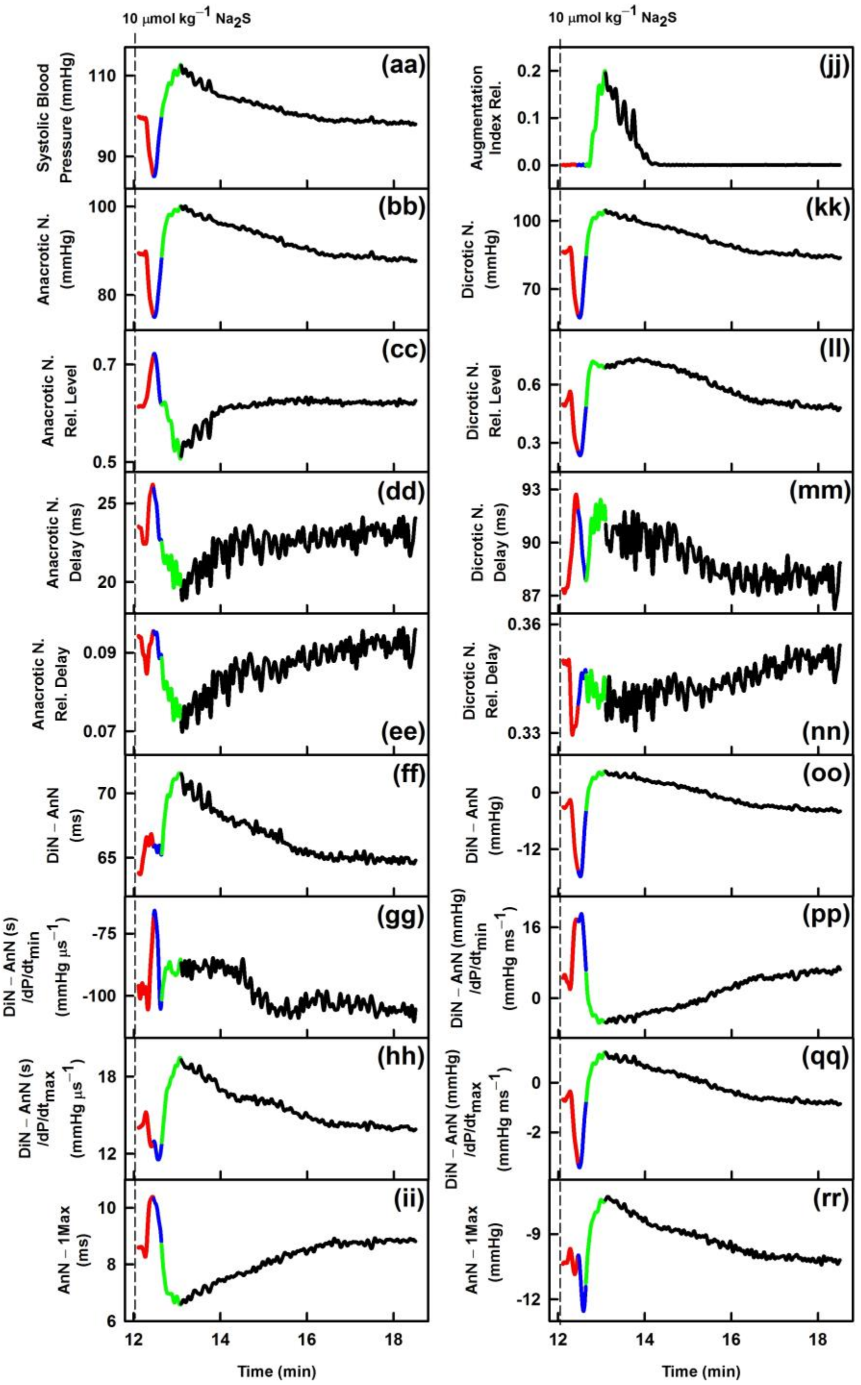

Figure S5. Time-dependent changes of HPs of anesthetized rat after I.V. bolus administration of 10 μmol kg⁻¹ Na_2S (marked by dash lines). Colors: time period corresponds to the decrease of systolic BP (red), increase of systolic BP to the control value (blue), further increase of systolic BP to maximum (green) and decrease of systolic BP to the control value (black). The red line starts 3 s before Na_2S administration.

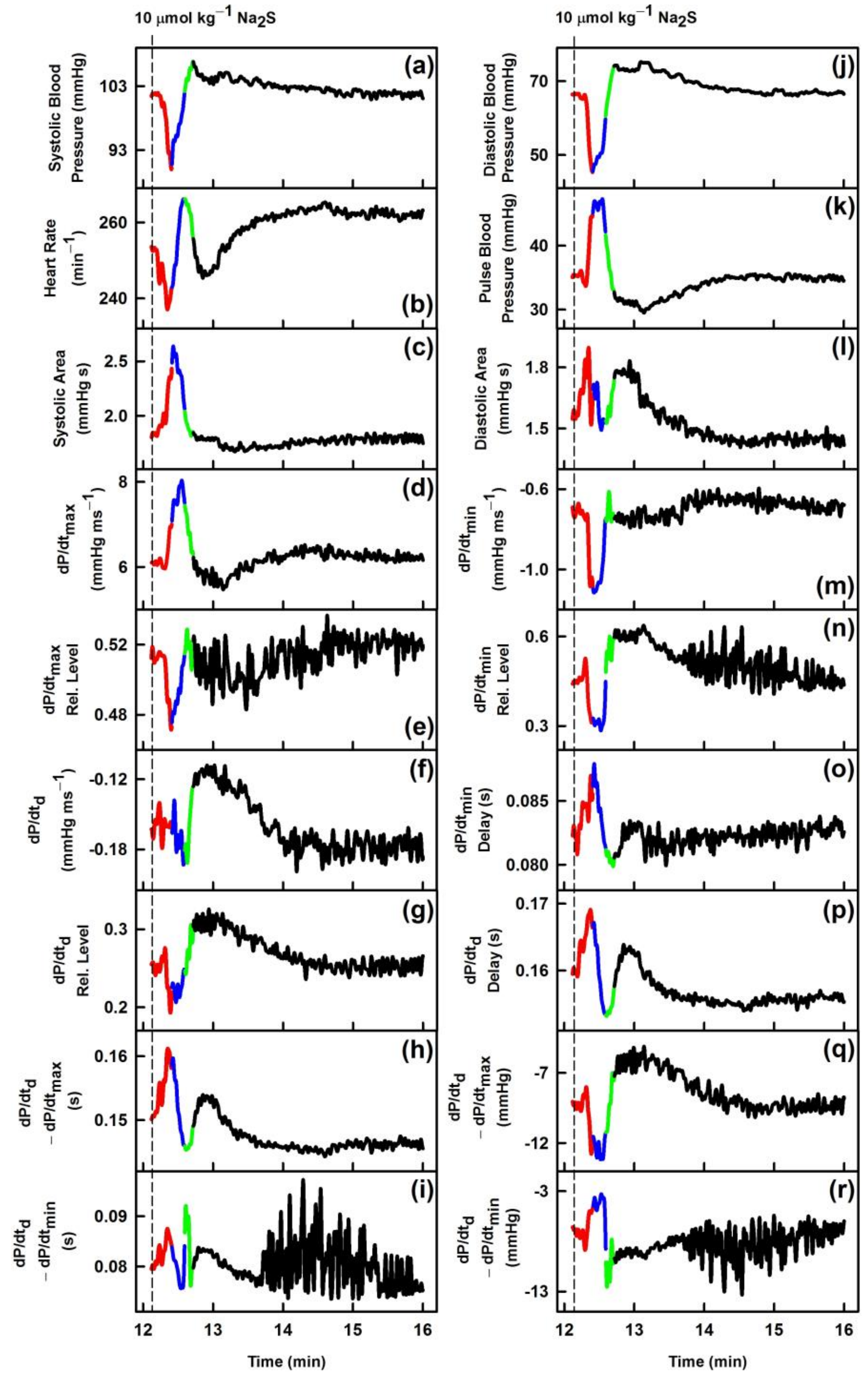

10 μmol kg⁻¹ Na2S
Systolic Blood Pressure (mmHg)
Heart Rate (min⁻¹)
Systolic Area (mmHg s)
dP/dtmax (mmHg ms⁻¹)
dP/dtmax Rel. Level
dP/dtd (mmHg ms⁻¹)
dP/dtd Rel. Level
dP/dtd – dP/dtmax (s)
dP/dtd – dP/dtmin (s)
Diastolic Blood Pressure (mmHg)
Pulse Blood Pressure (mmHg)
Diastolic Area (mmHg s)
dP/dtmin (mmHg ms⁻¹)
dP/dtmin Rel. Level
dP/dtmin Delay (s)
dP/dtd Delay (s)
dP/dtd – dP/dtmax (mmHg)
dP/dtd – dP/dtmin (mmHg)
Time (min)
(a)
(b)
(c)
(d)
(e)
(f)
(g)
(h)
(i)
(j)
(k)
(l)
(m)
(n)
(o)
(p)
(q)
(r)

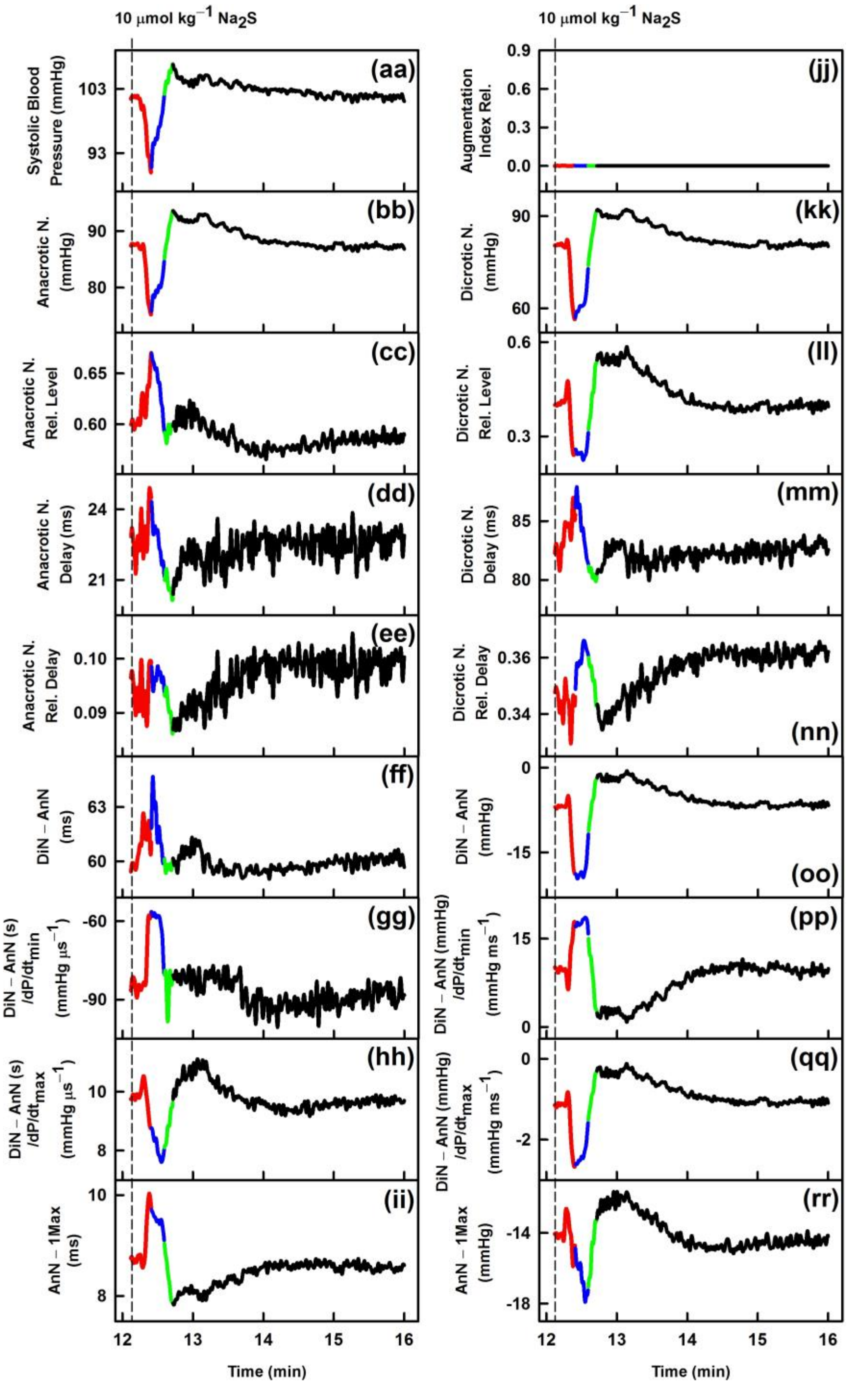

Figure S6. Time-dependent changes of HPs of anesthetized rat after I.V. bolus administration of 10 μmol kg−1 Na_2S (marked by dash lines). Colors: time period corresponds to the decrease of systolic BP (red), increase of systolic BP to the control value (blue), further increase of systolic BP to maximum (green) and decrease of systolic BP to the control value (black). The red line starts 3 s before Na_2S administration.

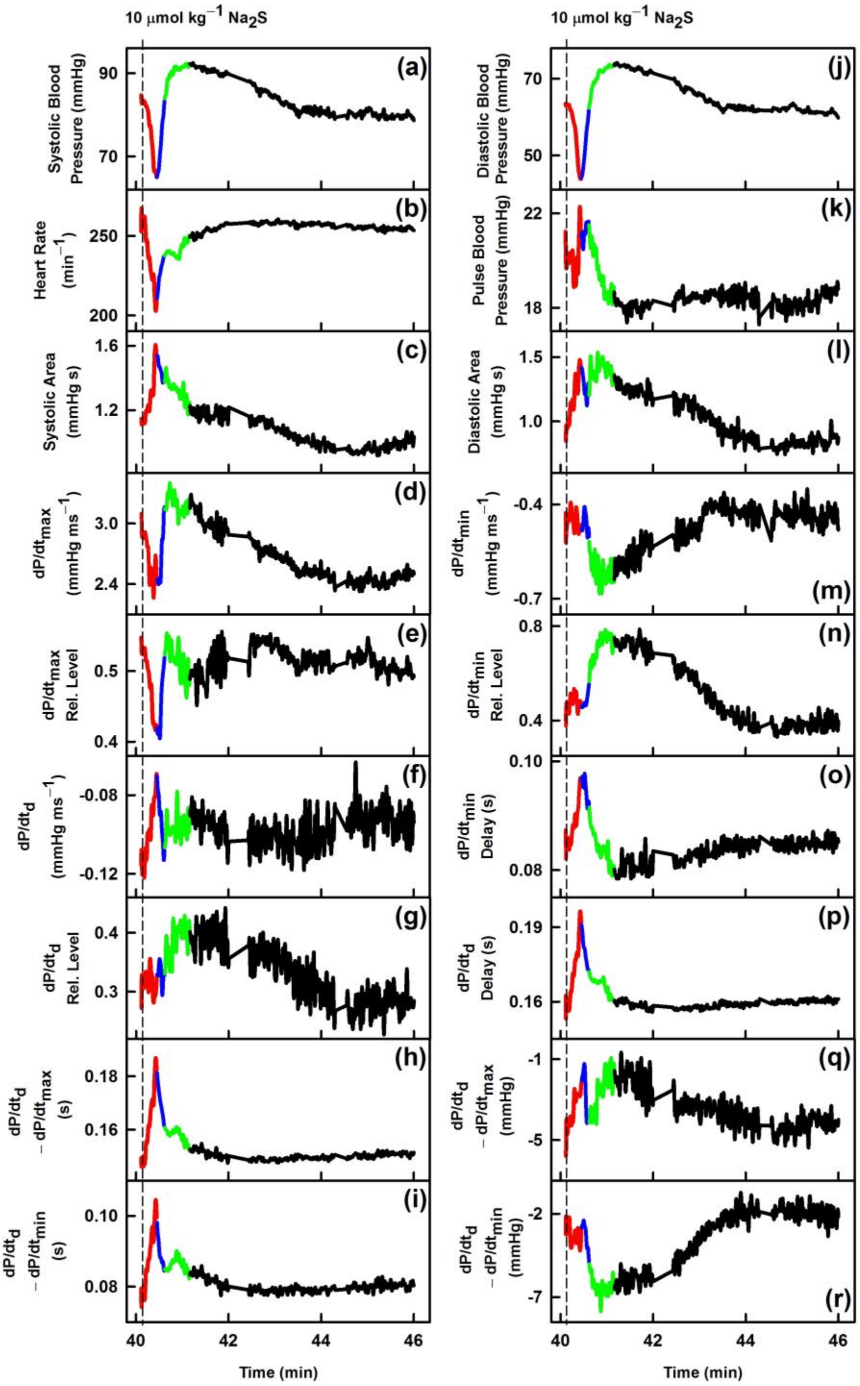

10 μmol kg−1 Na2S
Systolic Blood Pressure (mmHg)
Heart Rate (min−1)
Systolic Area (mmHg s)
dP/dtmax (mmHg ms−1)
dP/dtmax Rel. Level
dP/dtd (mmHg ms−1)
dP/dtd Rel. Level
dP/dtd − dP/dtmax (s)
dP/dtd − dP/dtmin (s)
Diastolic Blood Pressure (mmHg)
Pulse Blood Pressure (mmHg)
Diastolic Area (mmHg s)
dP/dtmin (mmHg ms−1)
dP/dtmin Rel. Level
dP/dtmin Delay (s)
dP/dtd Delay (s)
dP/dtd − dP/dtmax (mmHg)
dP/dtd − dP/dtmin (mmHg)
(a) (b) (c) (d) (e) (f) (g) (h) (i) (j) (k) (l) (m) (n) (o) (p) (q) (r)
Time (min)

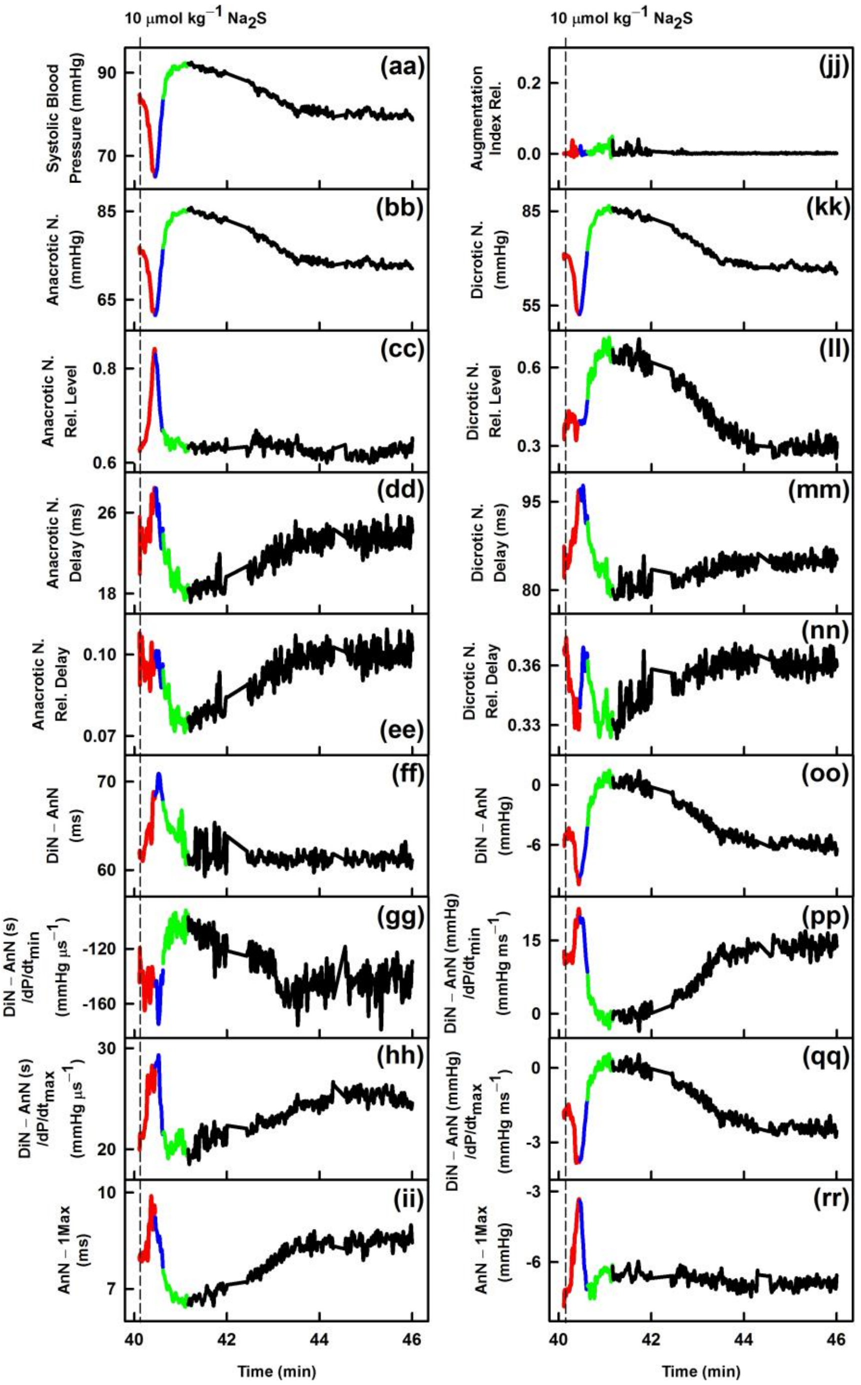

Figure S7. Time-dependent changes of HPs of anesthetized rat after I.V. bolus administration of 10 μmol kg^{-1} Na_2S (marked by dash lines). Colors: time period corresponds to the decrease of systolic BP (red), increase of systolic BP to the control value (blue), further increase of systolic BP to maximum (green) and decrease of systolic BP to the control value (black). The red line starts 3 s before Na_2S administration.

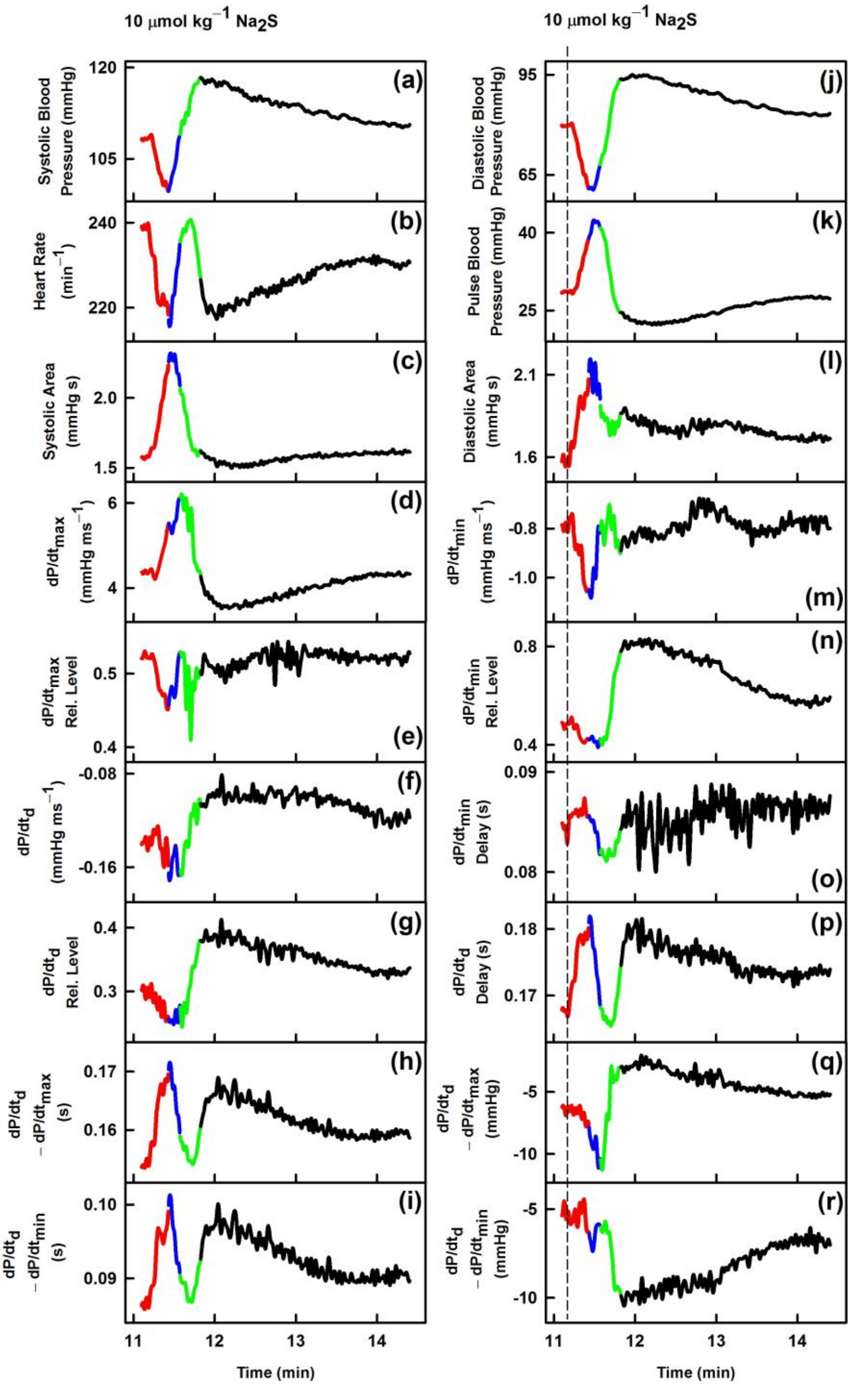

10 μmol kg−1 Na2S
10 μmol kg−1 Na2S
Systolic Blood Pressure (mmHg)
Heart Rate (min−1)
Systolic Area (mmHg s)
dP/dtmax (mmHg ms−1)
dP/dtmax Rel. Level
dP/dtd (mmHg ms−1)
dP/dtd Rel. Level
dP/dtd − dP/dtmax (s)
dP/dtd − dP/dtmin (s)
Diastolic Blood Pressure (mmHg)
Pulse Blood Pressure (mmHg)
Diastolic Area (mmHg s)
dP/dtmin (mmHg ms−1)
dP/dtmin Rel. Level
dP/dtmin Delay (s)
dP/dtd Delay (s)
dP/dtd − dP/dtmax (mmHg)
dP/dtd − dP/dtmin (mmHg)
(a) (b) (c) (d) (e) (f) (g) (h) (i) (j) (k) (l) (m) (n) (o) (p) (q) (r)
Time (min)
Time (min)

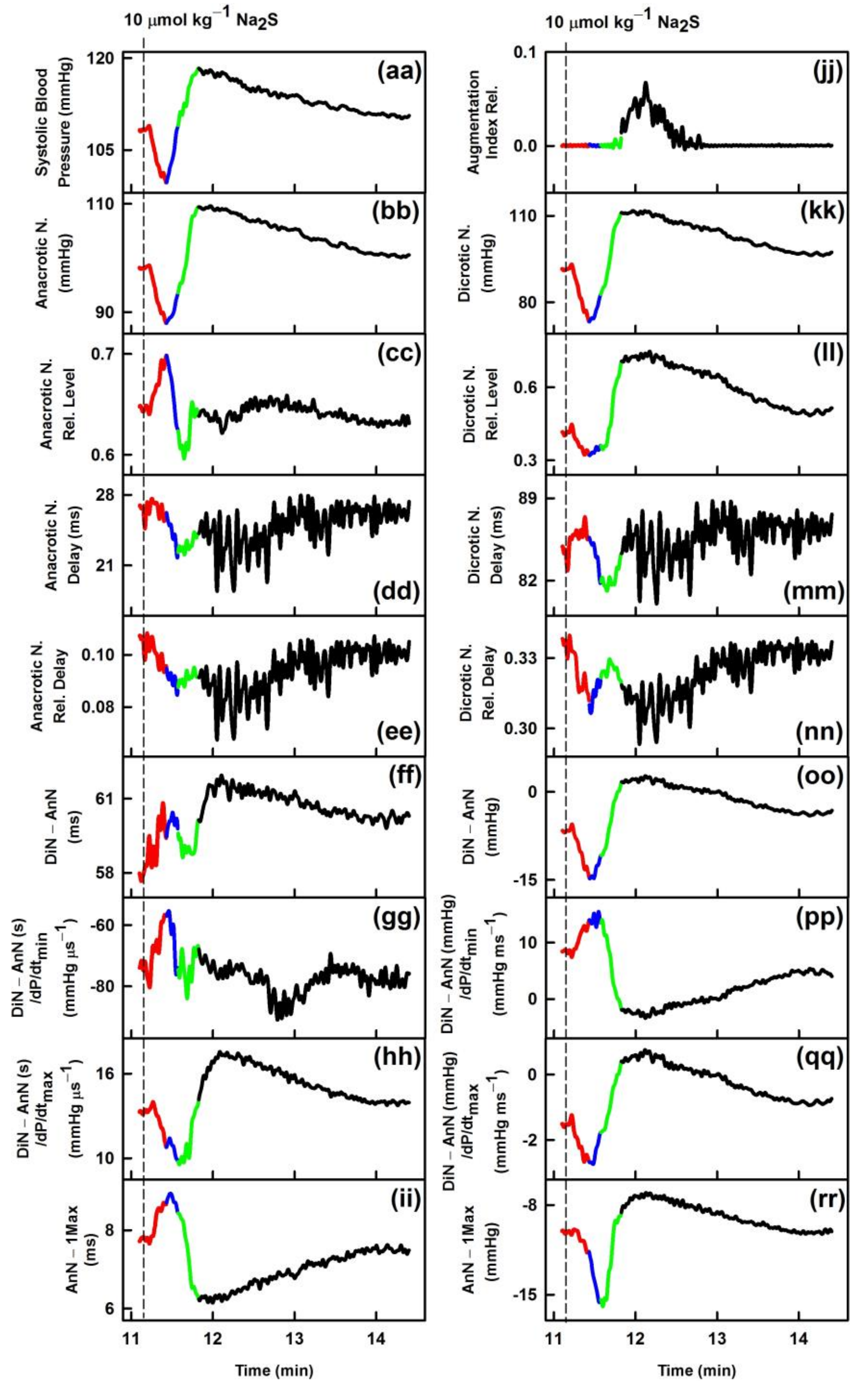

Figure S8. Time-dependent changes of HPs of anesthetized rat after I.V. bolus administration of 10 μmol kg⁻¹ Na_2S (marked by dash lines). Colors: time period corresponds to the decrease of systolic BP (red), increase of systolic BP to the control value (blue), further increase of systolic BP to maximum (green) and decrease of systolic BP to the control value (black). The red line starts 3 s before Na_2S administration.

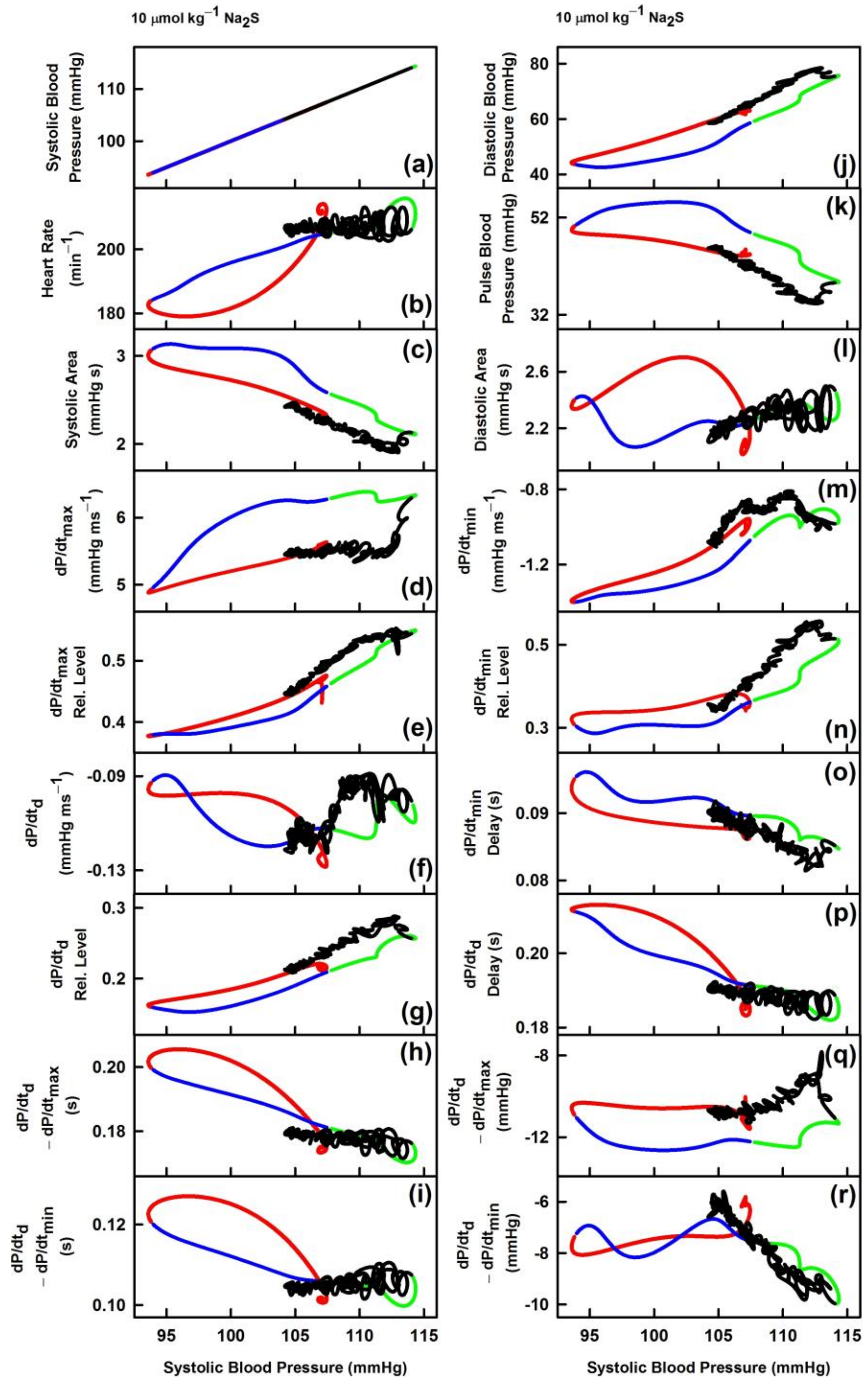

10 μmol kg−1 Na2S
10 μmol kg−1 Na2S
Systolic Blood Pressure (mmHg)
Heart Rate (min−1)
Systolic Area (mmHg s)
dP/dtmax (mmHg ms−1)
dP/dtmax Rel. Level
dP/dtd (mmHg ms−1)
dP/dtd Rel. Level
dP/dtd − dP/dtmax (s)
dP/dtd − dP/dtmin (s)
Diastolic Blood Pressure (mmHg)
Pulse Blood Pressure (mmHg)
Diastolic Area (mmHg s)
dP/dtmin (mmHg ms−1)
dP/dtmin Rel. Level
dP/dtmin Delay (s)
dP/dtd Delay (s)
dP/dtd − dP/dtmax (mmHg)
dP/dtd − dP/dtmin (mmHg)
(a)
(b)
(c)
(d)
(e)
(f)
(g)
(h)
(i)
(j)
(k)
(l)
(m)
(n)
(o)
(p)
(q)
(r)
95
100
105
110
115
Systolic Blood Pressure (mmHg)
Systolic Blood Pressure (mmHg)

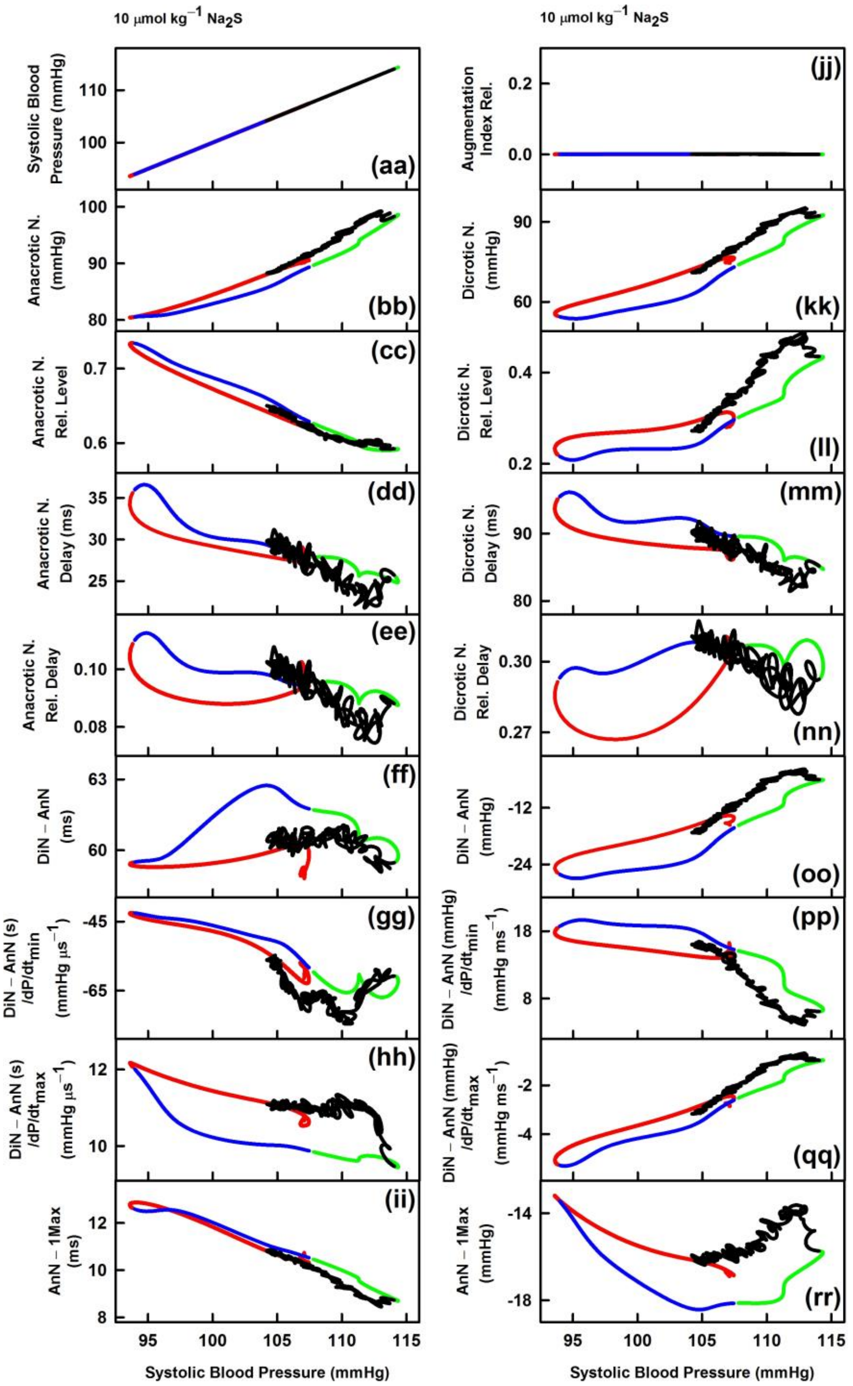

Figure S9. Relationships of HPs to the systolic BP after administration of 10 μmol kg⁻¹ Na_2S. The colors and (time-dependent) data correspond to Figure S2. The hysteresis was arbitrary defined as HP − Systolic BP (in mmHg) loop > 5 mmHg of Systolic BP.

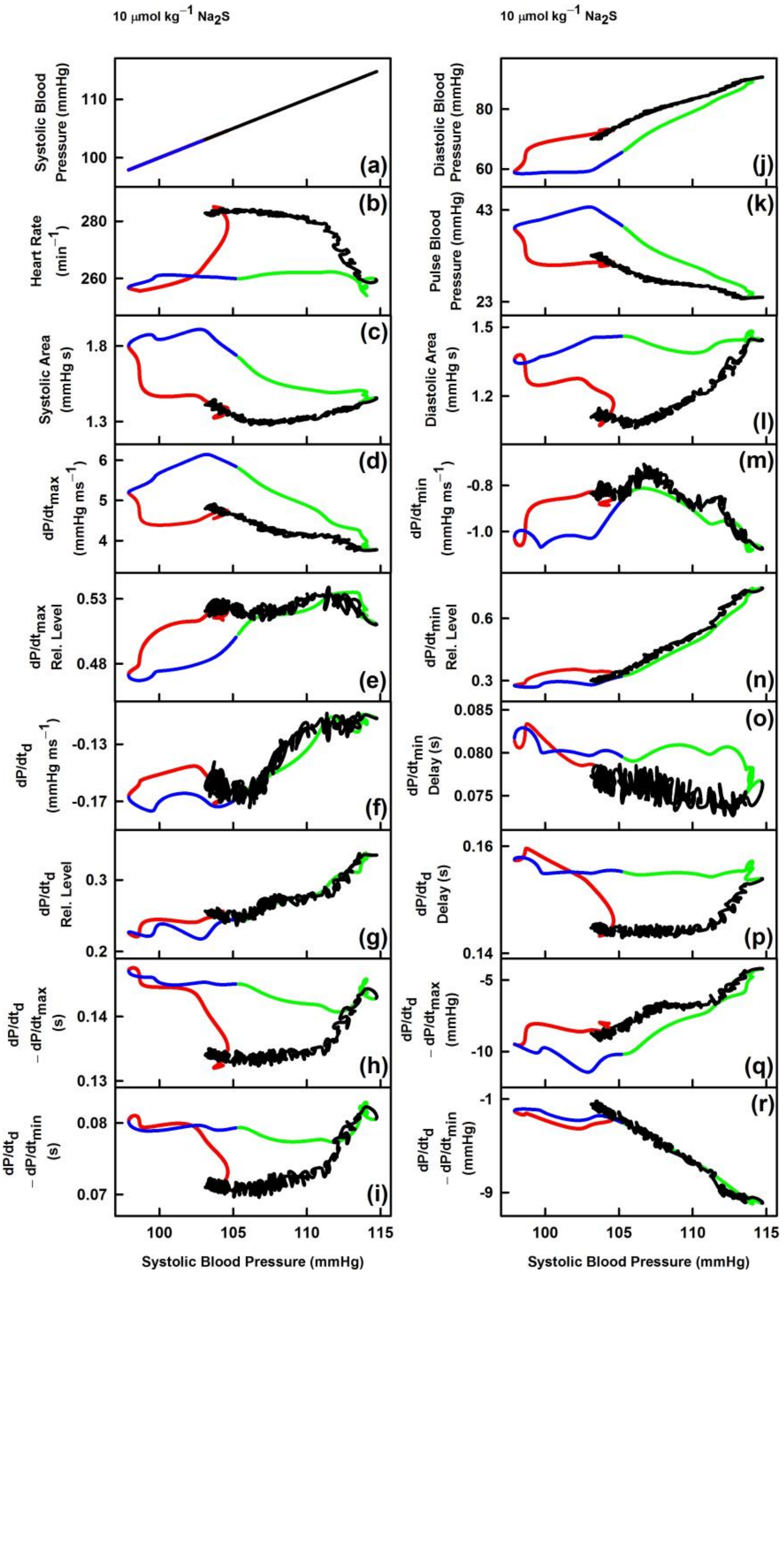

10 μmol kg−1 Na2S
10 μmol kg−1 Na2S
Systolic Blood Pressure (mmHg)
Heart Rate (min−1)
Systolic Area (mmHg s)
dP/dtmax (mmHg ms−1)
dP/dtmax Rel. Level
dP/dtd (mmHg ms−1)
dP/dtd Rel. Level
dP/dtd − dP/dtmax (s)
dP/dtd − dP/dtmin (s)
Diastolic Blood Pressure (mmHg)
Pulse Blood Pressure (mmHg)
Diastolic Area (mmHg s)
dP/dtmin (mmHg ms−1)
dP/dtmin Rel. Level
dP/dtmin Delay (s)
dP/dtd Delay (s)
dP/dtd − dP/dtmax (mmHg)
dP/dtd − dP/dtmin (mmHg)
(a)
(b)
(c)
(d)
(e)
(f)
(g)
(h)
(i)
(j)
(k)
(l)
(m)
(n)
(o)
(p)
(q)
(r)
Systolic Blood Pressure (mmHg)
Systolic Blood Pressure (mmHg)

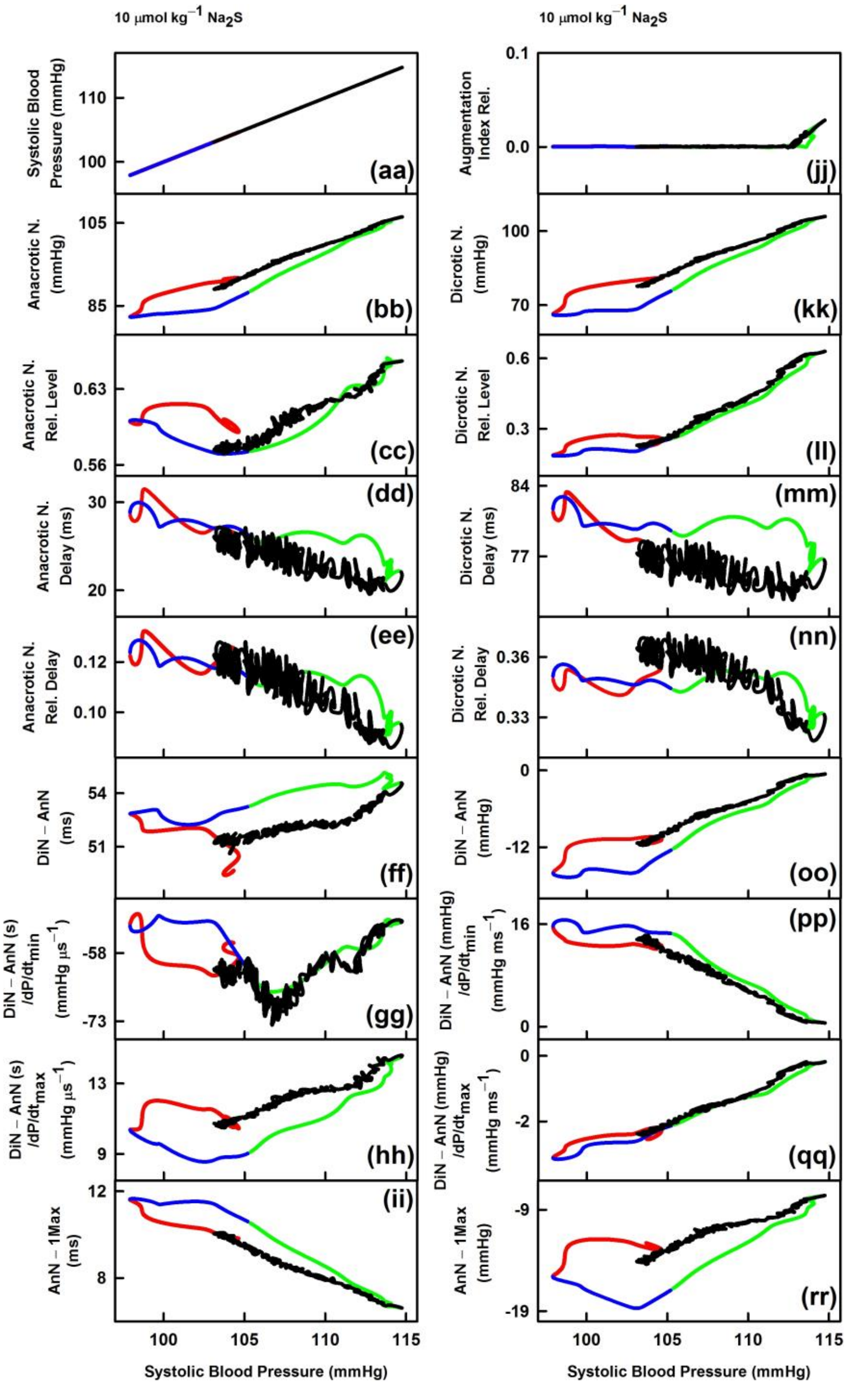

Figure S10. Relationships of HPs to the systolic BP after administration of 10 μmol kg^{-1} Na_2S. The colors and (time-dependent) data correspond to Figure S3. The hysteresis was arbitrary defined as HP − Systolic BP (in mmHg) loop > 5 mmHg of Systolic BP.

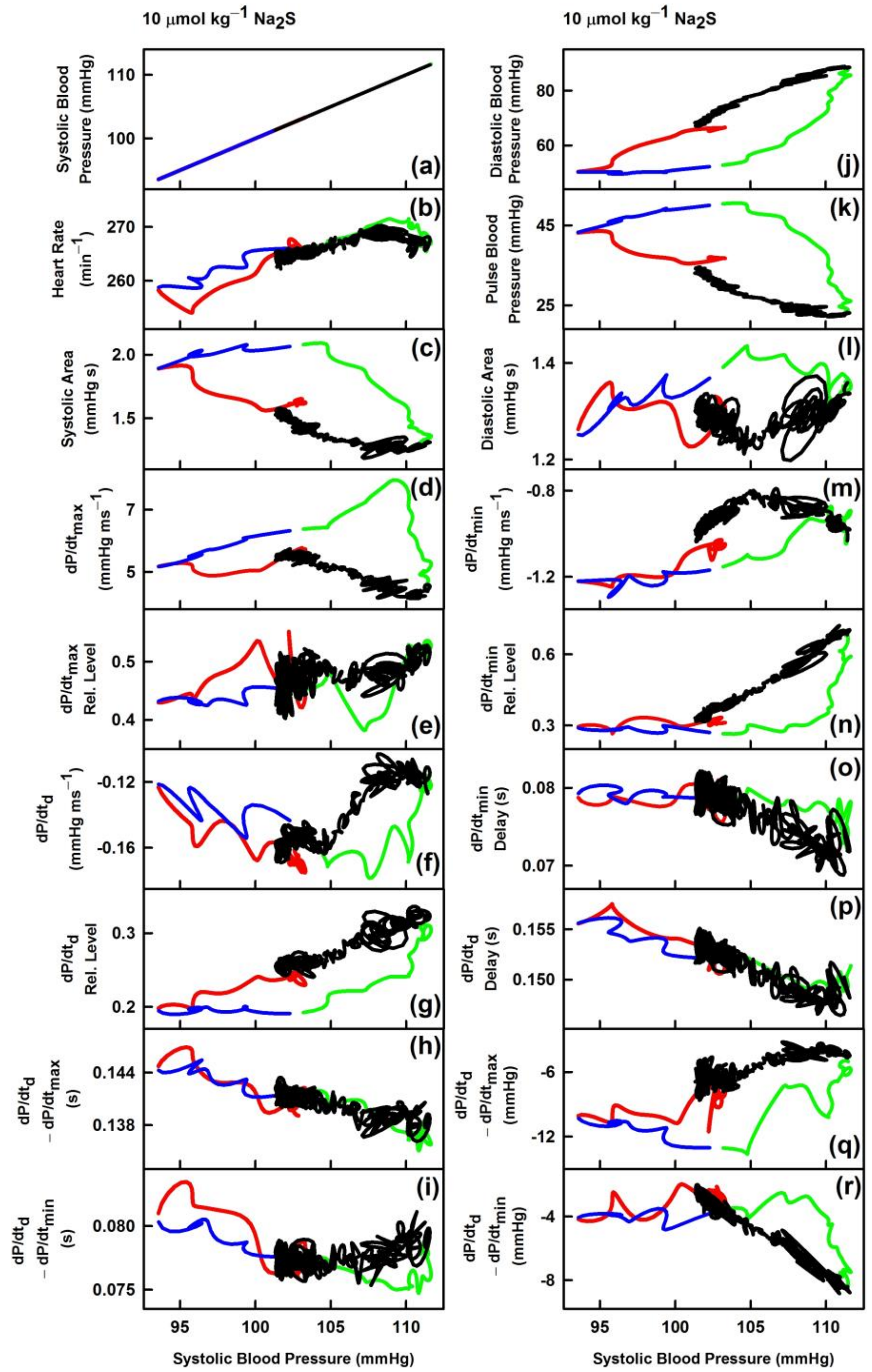

10 μmol kg−1 Na2S
10 μmol kg−1 Na2S
Systolic Blood Pressure (mmHg)
Heart Rate (min−1)
Systolic Area (mmHg s)
dP/dtmax (mmHg ms−1)
dP/dtmax Rel. Level
dP/dtd (mmHg ms−1)
dP/dtd Rel. Level
dP/dtd − dP/dtmax (s)
dP/dtd − dP/dtmin (s)
Diastolic Blood Pressure (mmHg)
Pulse Blood Pressure (mmHg)
Diastolic Area (mmHg s)
dP/dtmin (mmHg ms−1)
dP/dtmin Rel. Level
dP/dtmin Delay (s)
dP/dtd Delay (s)
dP/dtd − dP/dtmax (mmHg)
dP/dtd − dP/dtmin (mmHg)
(a)
(b)
(c)
(d)
(e)
(f)
(g)
(h)
(i)
(j)
(k)
(l)
(m)
(n)
(o)
(p)
(q)
(r)
Systolic Blood Pressure (mmHg)
Systolic Blood Pressure (mmHg)

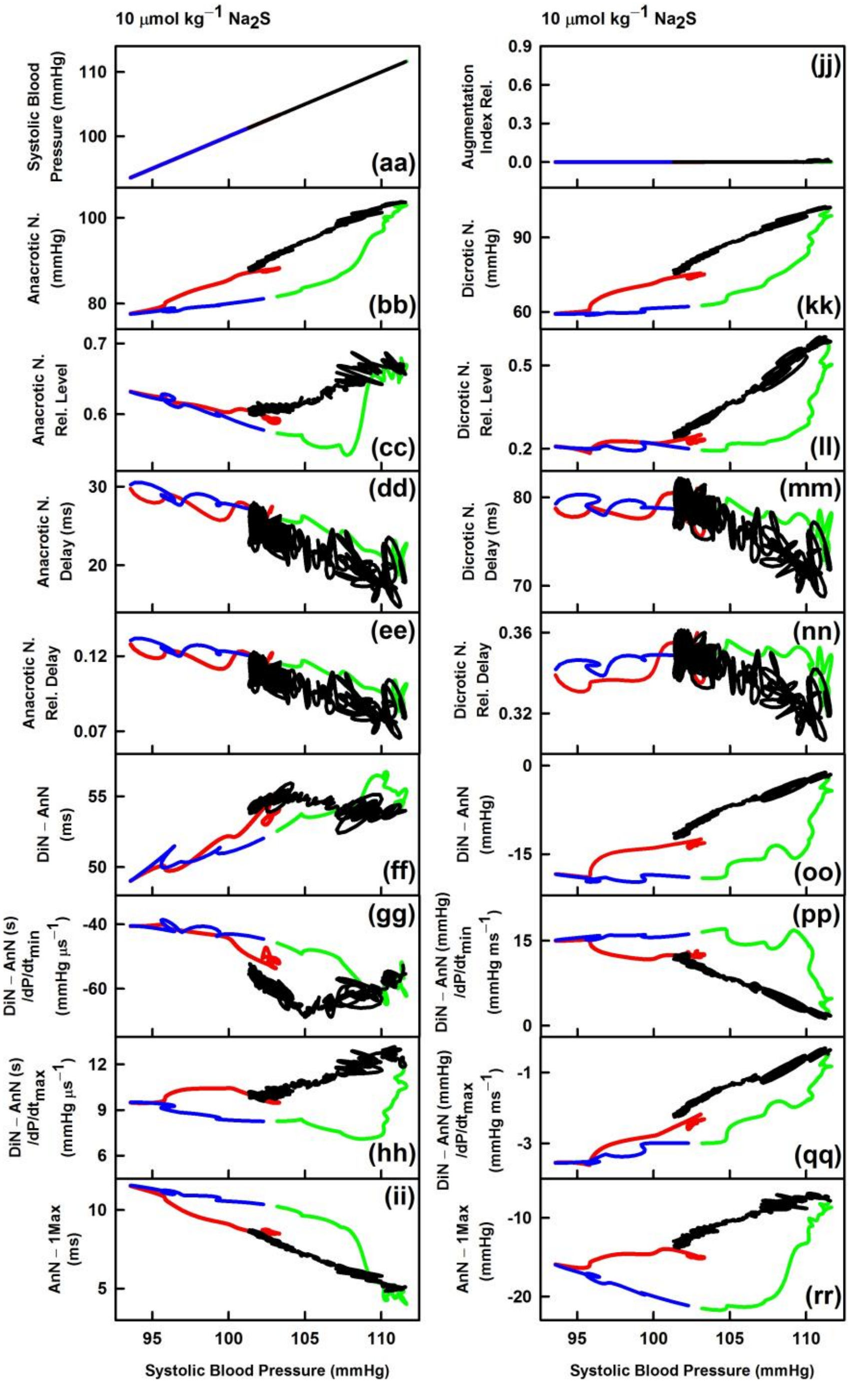

Figure S11. Relationships of HPs to the systolic BP after administration of 10 μmol kg⁻¹ Na_2S. The colors and (time-dependent) data correspond to Figure S4. The hysteresis was arbitrary defined as HP − Systolic BP (in mmHg) loop > 5 mmHg of Systolic BP.

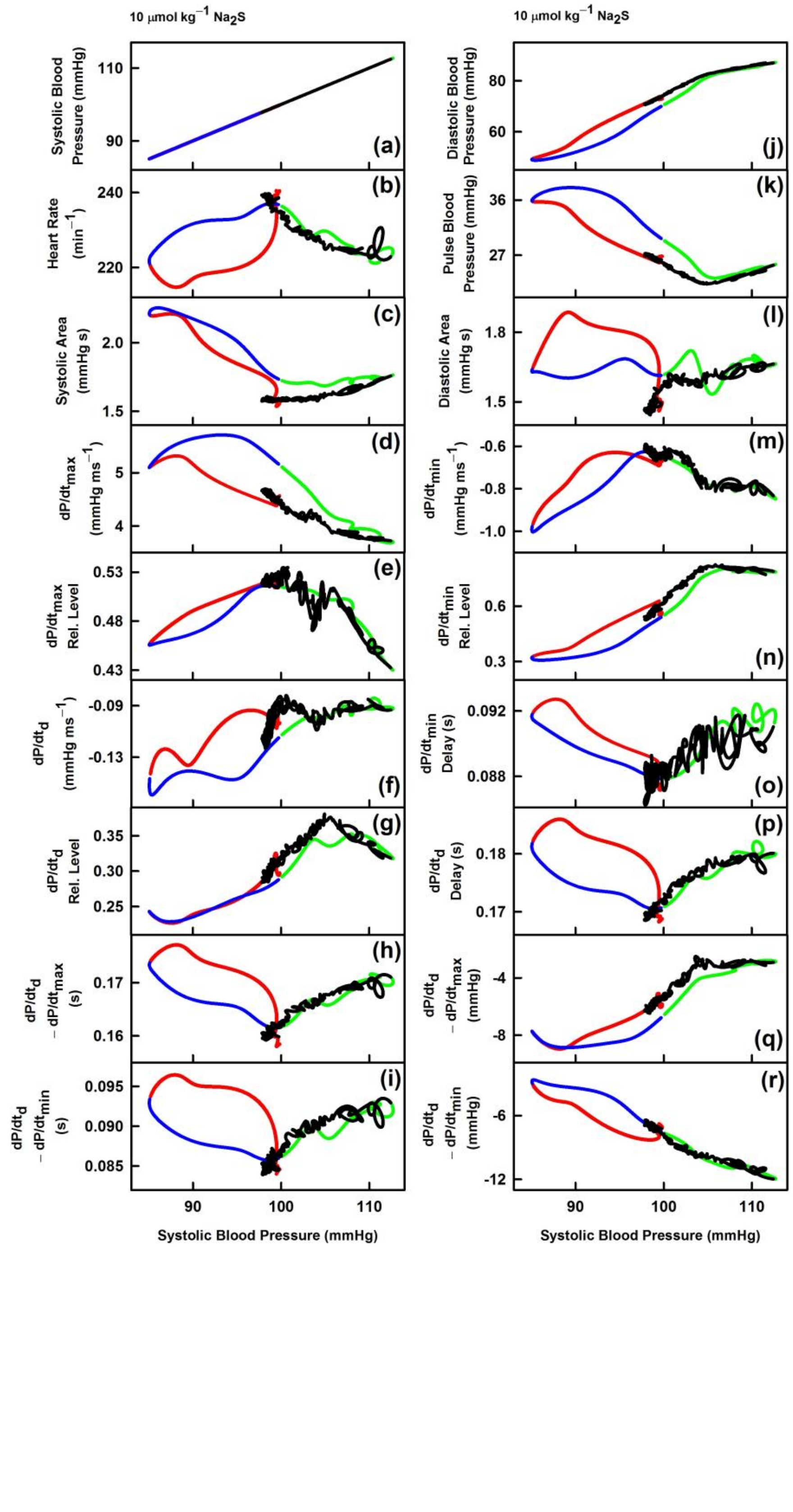

10 μmol kg−1 Na2S
10 μmol kg−1 Na2S
Systolic Blood Pressure (mmHg)
Heart Rate (min−1)
Systolic Area (mmHg s)
dP/dtmax (mmHg ms−1)
dP/dtmax Rel. Level
dP/dtd (mmHg ms−1)
dP/dtd Rel. Level
dP/dtd − dP/dtmax (s)
dP/dtd − dP/dtmin (s)
Diastolic Blood Pressure (mmHg)
Pulse Blood Pressure (mmHg)
Diastolic Area (mmHg s)
dP/dtmin (mmHg ms−1)
dP/dtmin Rel. Level
dP/dtmin Delay (s)
dP/dtd Delay (s)
dP/dtd − dP/dtmax (mmHg)
dP/dtd − dP/dtmin (mmHg)
(a)
(b)
(c)
(d)
(e)
(f)
(g)
(h)
(i)
(j)
(k)
(l)
(m)
(n)
(o)
(p)
(q)
(r)
Systolic Blood Pressure (mmHg)
Systolic Blood Pressure (mmHg)

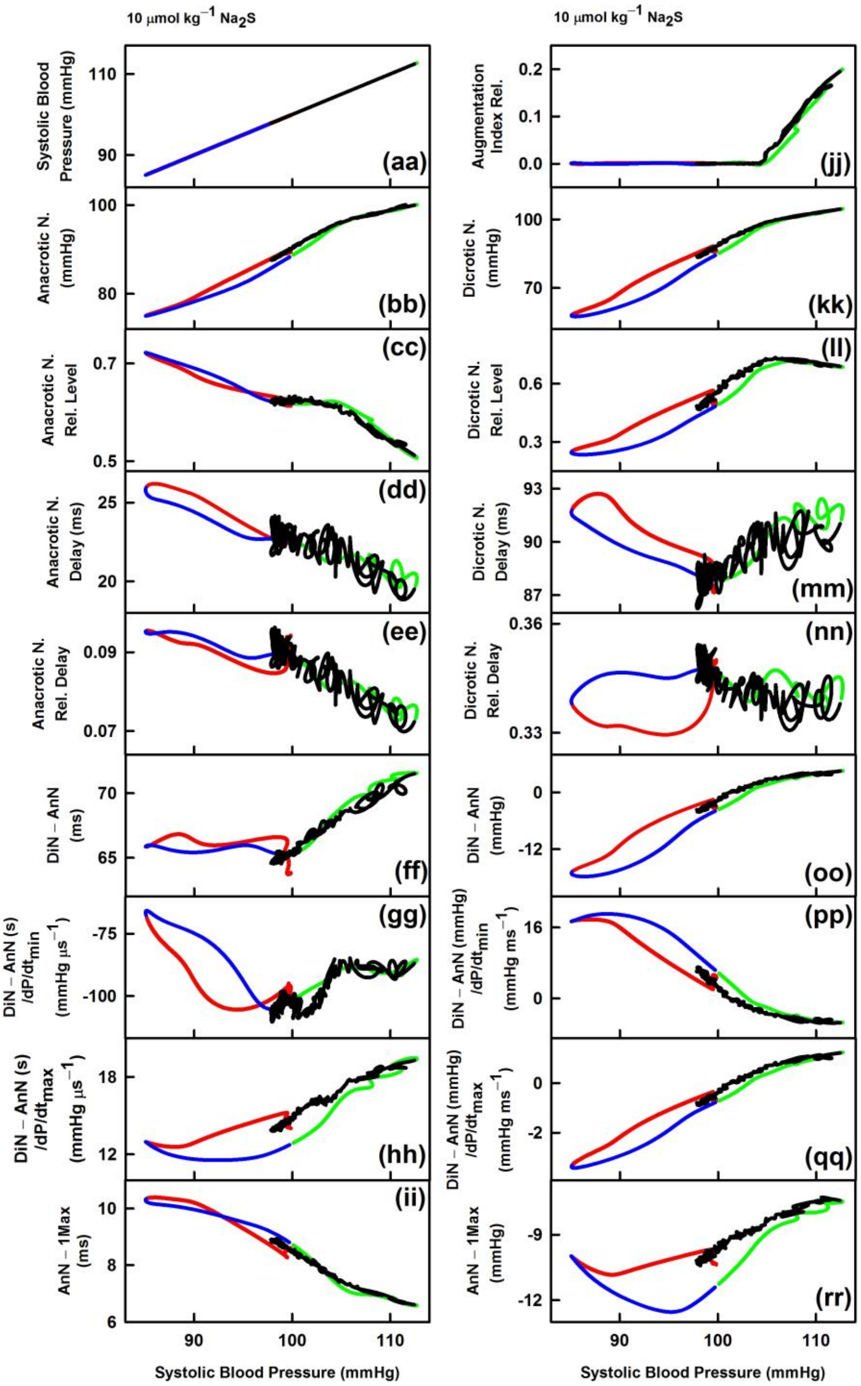

Figure S12. Relationships of HPs to the systolic BP after administration of 10 μmol kg−1 Na_2S. The colors and (time-dependent) data correspond to Figure S5. The hysteresis was arbitrary defined as HP − Systolic BP (in mmHg) loop > 5 mmHg of Systolic BP.

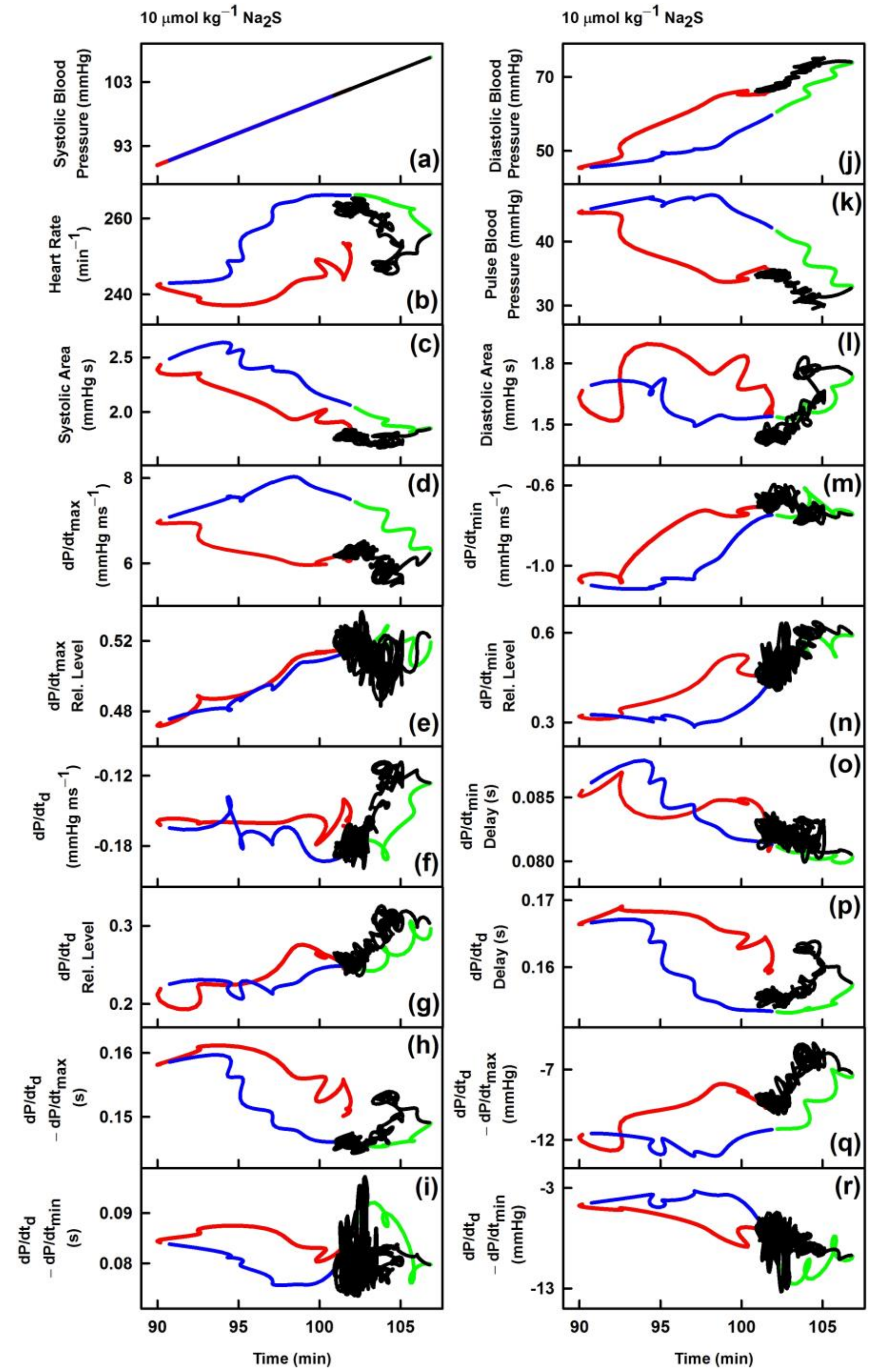
10 μmol kg−1 Na2S
10 μmol kg−1 Na2S
Systolic Blood Pressure (mmHg)
103
93
(a)
Heart Rate (min−1)
260
240
(b)
Systolic Area (mmHg s)
2.5
2.0
1.5
(c)
dP/dtmax (mmHg ms−1)
8
6
(d)
dP/dtmax Rel. Level
0.52
0.48
(e)
dP/dtd (mmHg ms−1)
-0.12
-0.18
(f)
dP/dtd Rel. Level
0.3
0.2
(g)
dP/dtd − dP/dtmax (s)
0.16
0.15
(h)
dP/dtd − dP/dtmin (s)
0.09
0.08
(i)
90
95
100
105
Time (min)
Diastolic Blood Pressure (mmHg)
70
50
(j)
Pulse Blood Pressure (mmHg)
40
30
(k)
Diastolic Area (mmHg s)
1.8
1.5
(l)
dP/dtmin (mmHg ms−1)
-0.6
-1.0
(m)
dP/dtmin Rel. Level
0.6
0.3
(n)
dP/dtmin Delay (s)
0.085
0.080
(o)
dP/dtd Delay (s)
0.17
0.16
(p)
dP/dtd − dP/dtmax (mmHg)
-7
-12
(q)
dP/dtd − dP/dtmin (mmHg)
-3
-13
(r)
90
95
100
105
Time (min)

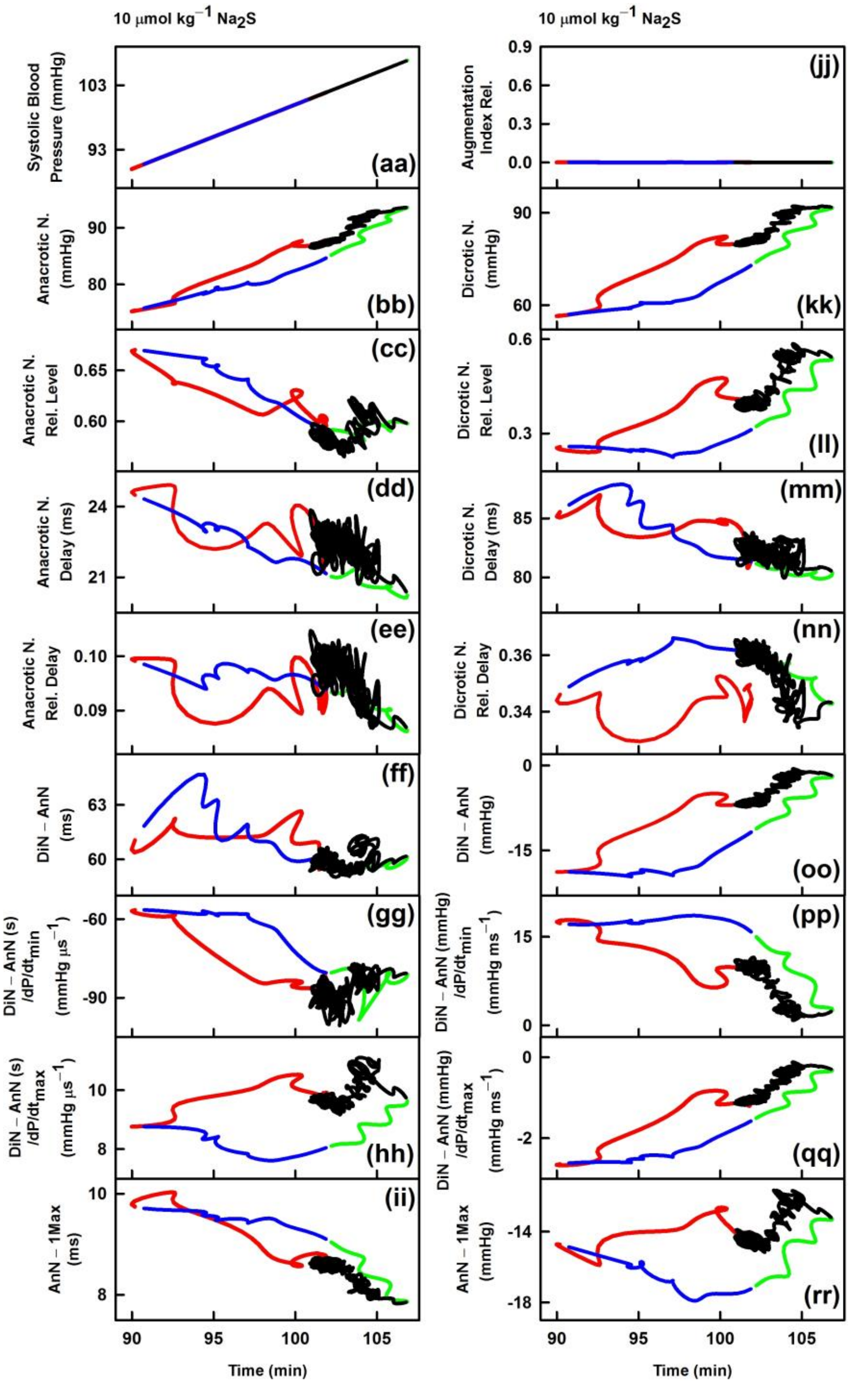

Figure S13. Relationships of HPs to the systolic BP after administration of 10 μmol kg[−1] Na_2S. The colors and (time-dependent) data correspond to Figure S6. The hysteresis was arbitrary defined as HP − Systolic BP (in mmHg) loop > 5 mmHg of Systolic BP.

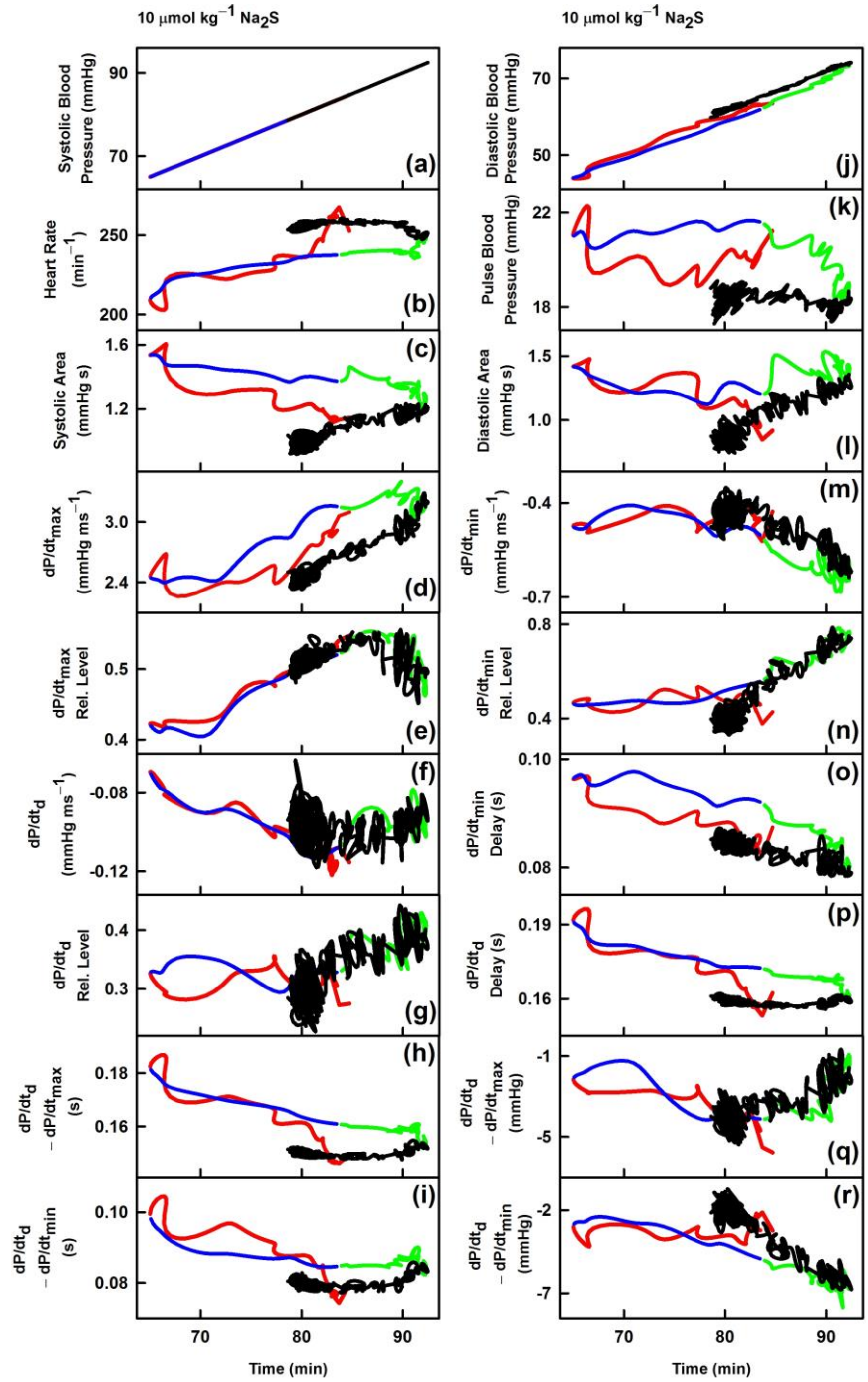

10 μmol kg−1 Na2S
10 μmol kg−1 Na2S
Systolic Blood Pressure (mmHg)
Heart Rate (min−1)
Systolic Area (mmHg s)
dP/dtmax (mmHg ms−1)
dP/dtmax Rel. Level
dP/dtd (mmHg ms−1)
dP/dtd Rel. Level
dP/dtd − dP/dtmax (s)
dP/dtd − dP/dtmin (s)
Diastolic Blood Pressure (mmHg)
Pulse Blood Pressure (mmHg)
Diastolic Area (mmHg s)
dP/dtmin (mmHg ms−1)
dP/dtmin Rel. Level
dP/dtmin Delay (s)
dP/dtd Delay (s)
dP/dtd − dP/dtmax (mmHg)
dP/dtd − dP/dtmin (mmHg)
(a)
(b)
(c)
(d)
(e)
(f)
(g)
(h)
(i)
(j)
(k)
(l)
(m)
(n)
(o)
(p)
(q)
(r)
Time (min)
Time (min)

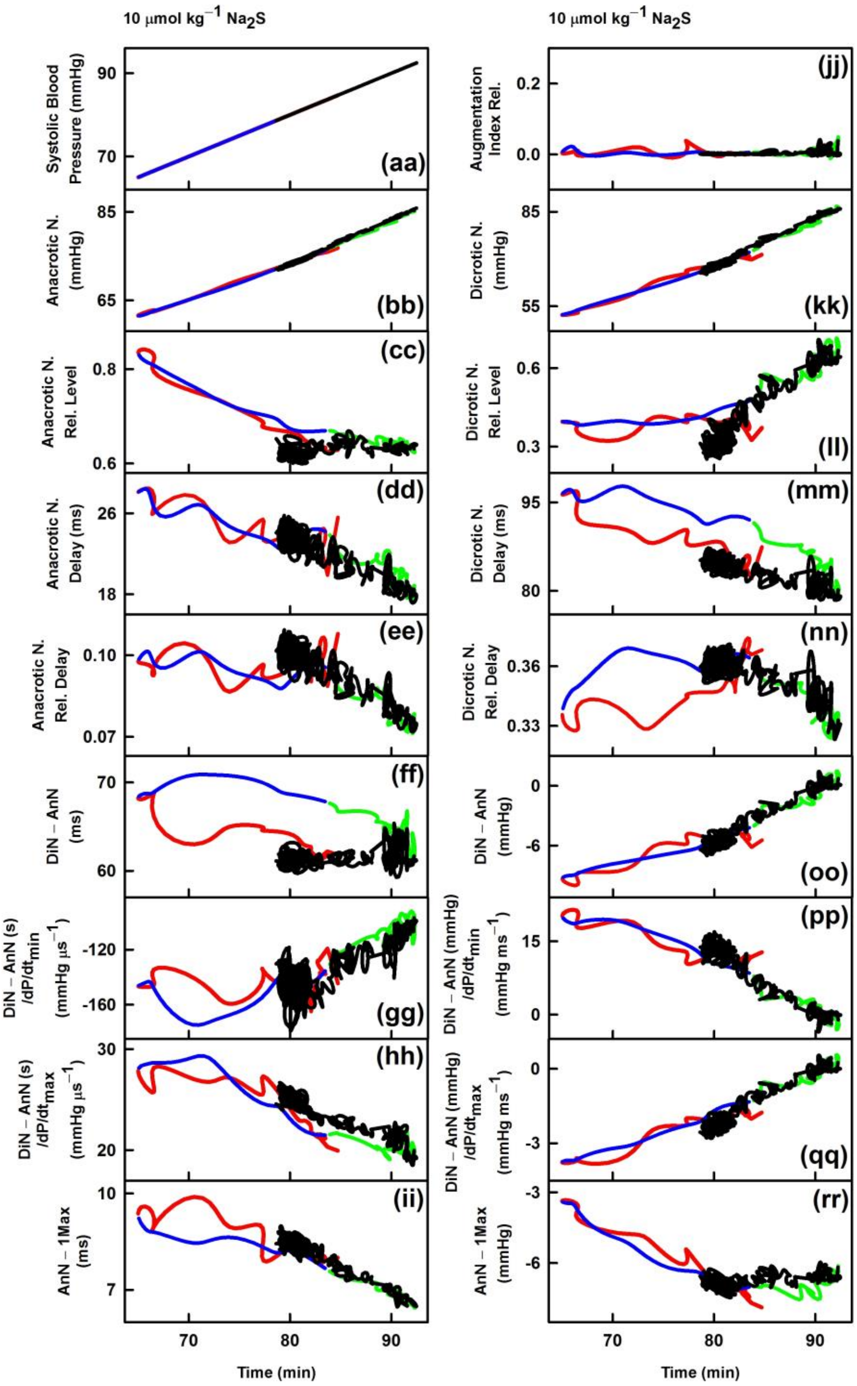

Figure S14. Relationships of HPs to the systolic BP after administration of 10 μmol kg[−1] Na_2S. The colors and (time-dependent) data correspond to Figure S7. The hysteresis was arbitrary defined as HP − Systolic BP (in mmHg) loop > 5 mmHg of Systolic BP.

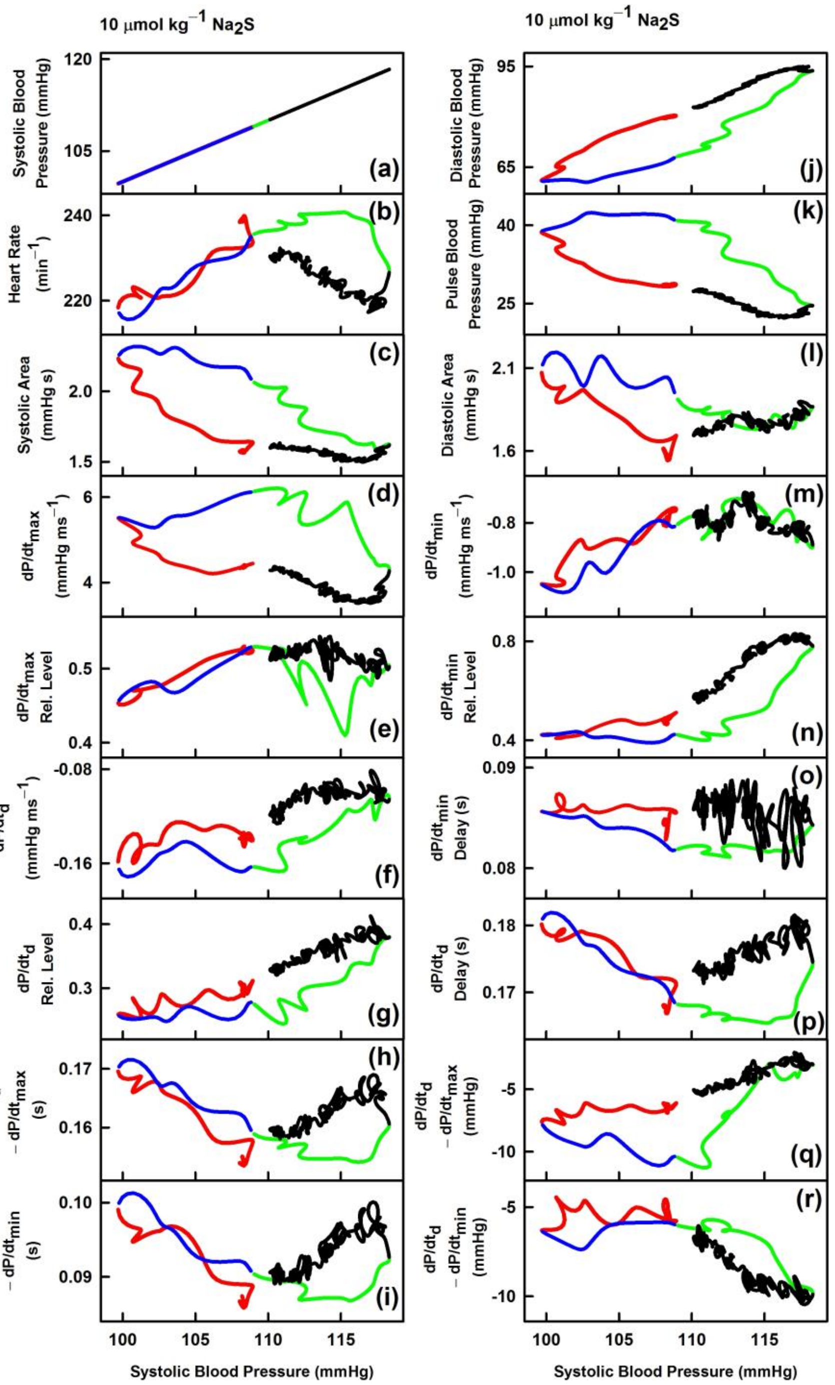
10 μmol kg−1 Na2S
10 μmol kg−1 Na2S
Systolic Blood Pressure (mmHg)
Heart Rate (min−1)
Systolic Area (mmHg s)
dP/dtmax (mmHg ms−1)
dP/dtmax Rel. Level
dP/dtd (mmHg ms−1)
dP/dtd Rel. Level
dP/dtd − dP/dtmax (s)
dP/dtd − dP/dtmin (s)
Diastolic Blood Pressure (mmHg)
Pulse Blood Pressure (mmHg)
Diastolic Area (mmHg s)
dP/dtmin (mmHg ms−1)
dP/dtmin Rel. Level
dP/dtmin Delay (s)
dP/dtd Delay (s)
dP/dtd − dP/dtmax (mmHg)
dP/dtd − dP/dtmin (mmHg)
(a)
(b)
(c)
(d)
(e)
(f)
(g)
(h)
(i)
(j)
(k)
(l)
(m)
(n)
(o)
(p)
(q)
(r)
Systolic Blood Pressure (mmHg)
Systolic Blood Pressure (mmHg)

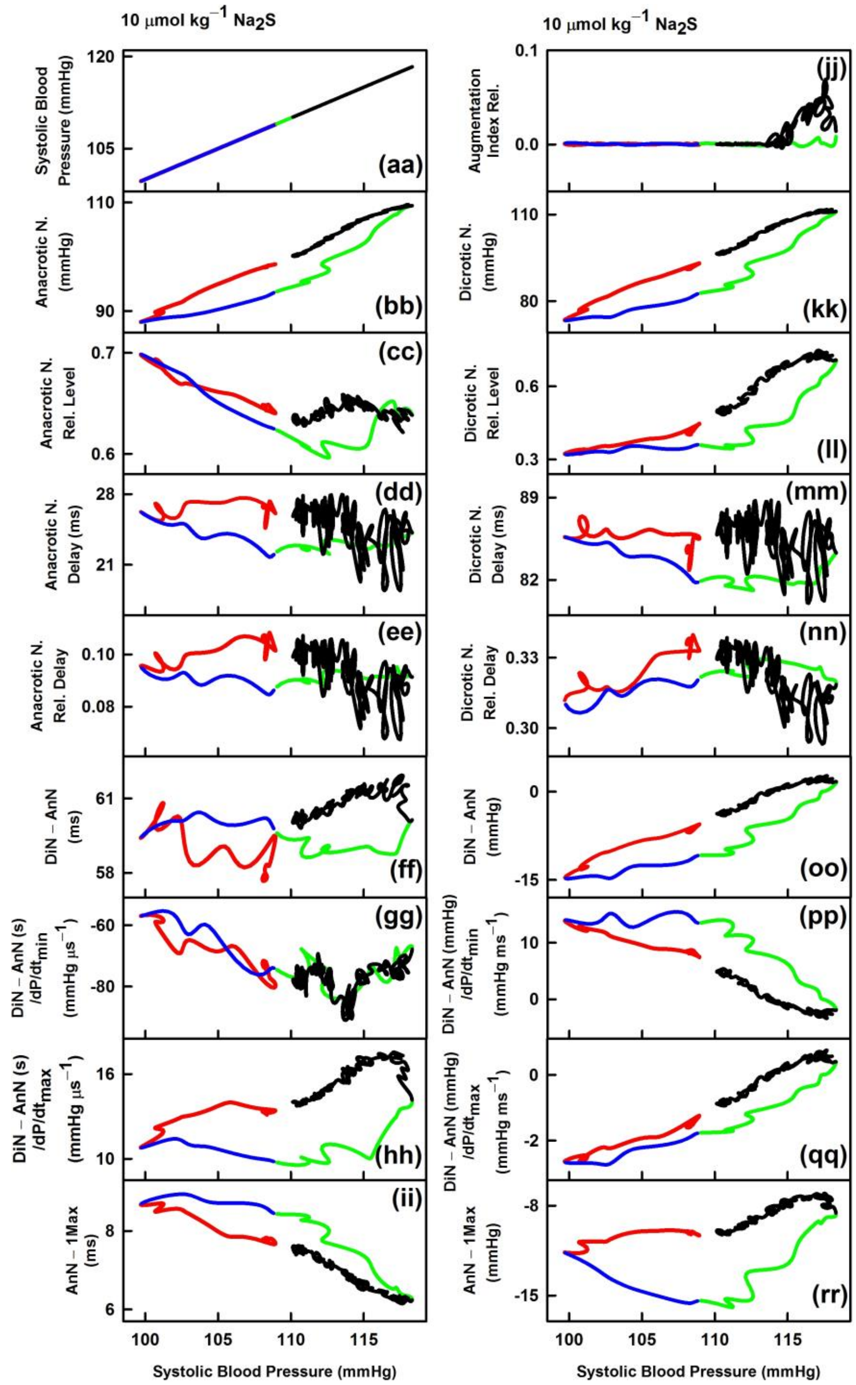

Figure S15. Relationships of HPs to the systolic BP after administration of 10 μmol kg[−1] Na_2S. The colors and (time-dependent) data correspond to Figure S8. The hysteresis was arbitrary defined as HP − Systolic BP (in mmHg) loop > 5 mmHg of Systolic BP.

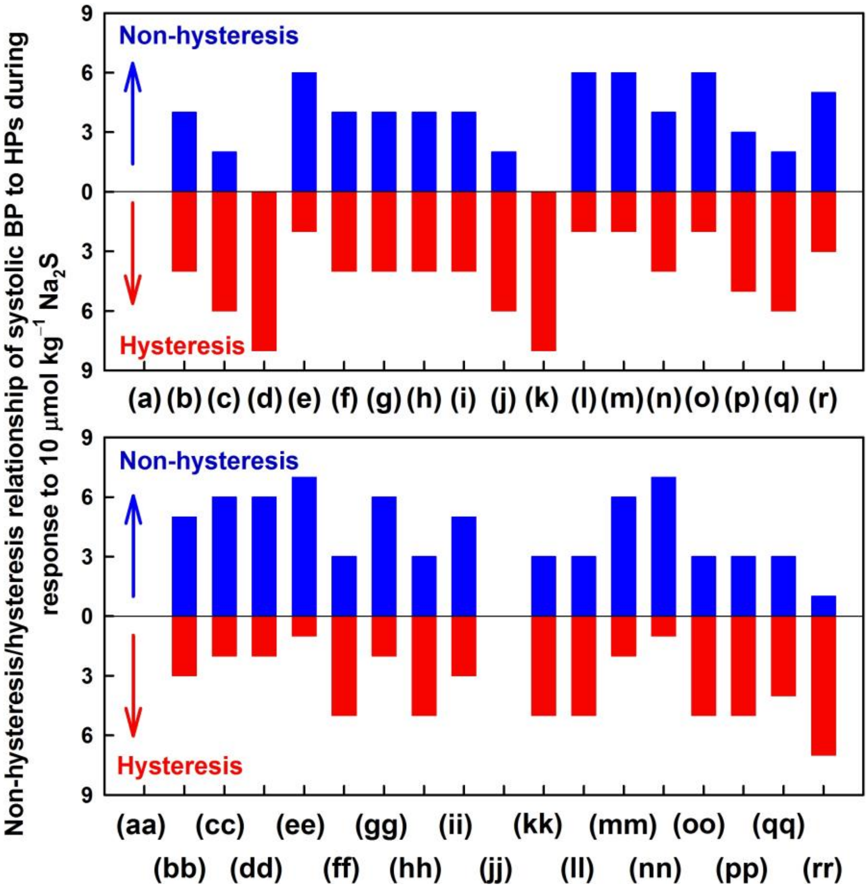

Figure S16. Number of rats representing the non-hysteresis/hysteresis patterns of the relationships of HPs to systolic BP after administrations of 10 μmol kg⁻¹ Na_2S during increase (green line in Figures 2 and S9-S15) and decrease (black line in Figures 2 and S9-S15) of BP. Data were taken from Figures 2 and S9-S15. The total number of rats in which non-hysteresis (blue) or hysteresis (red) patterns were evaluated is n = 8. The hysteresis was arbitrary defined as HP – systolic BP loop > 5 mmHg of systolic BP.

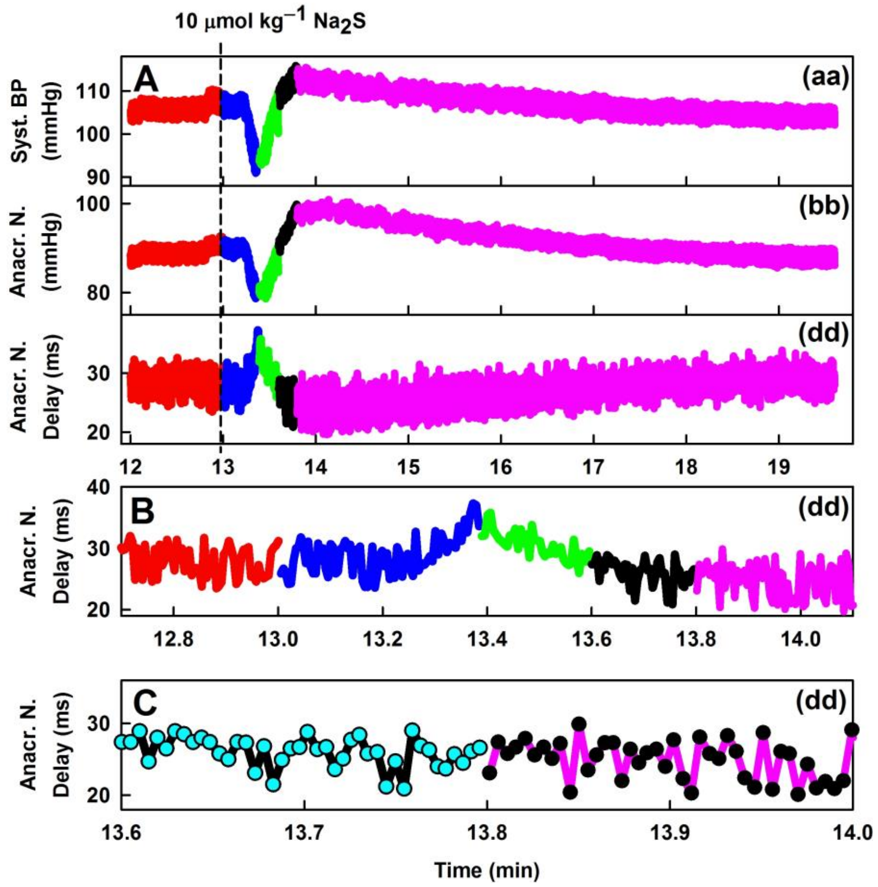

Figure S17. Time-dependent effect of 10 µmol kg⁻¹ Na_2S on three HPs: (**A**): systolic BP (Syst. BP, mmHg) **(aa)**, AnN (Anacr. N., mmHg) **(bb)** and AnN delay (Anacr. N. delay, ms) **(dd)**; (12-20 min). (**B**): The fluctuation of AnN delay **(dd)**; (13-14 min) reflects the time interval fluctuation between **a1** and **a2** points (Figure S1). The higher value of AnN delay in ms indicates that diastolic BP at the point **a1** was lower than at the point **a2**. (**C**): The example of the fluctuation between **a1** and **a2** points in pulses (circles) showed at high resolution after the 10 µmol kg⁻¹ Na_2S administration **(dd)**. Data were taken from Figure S2.

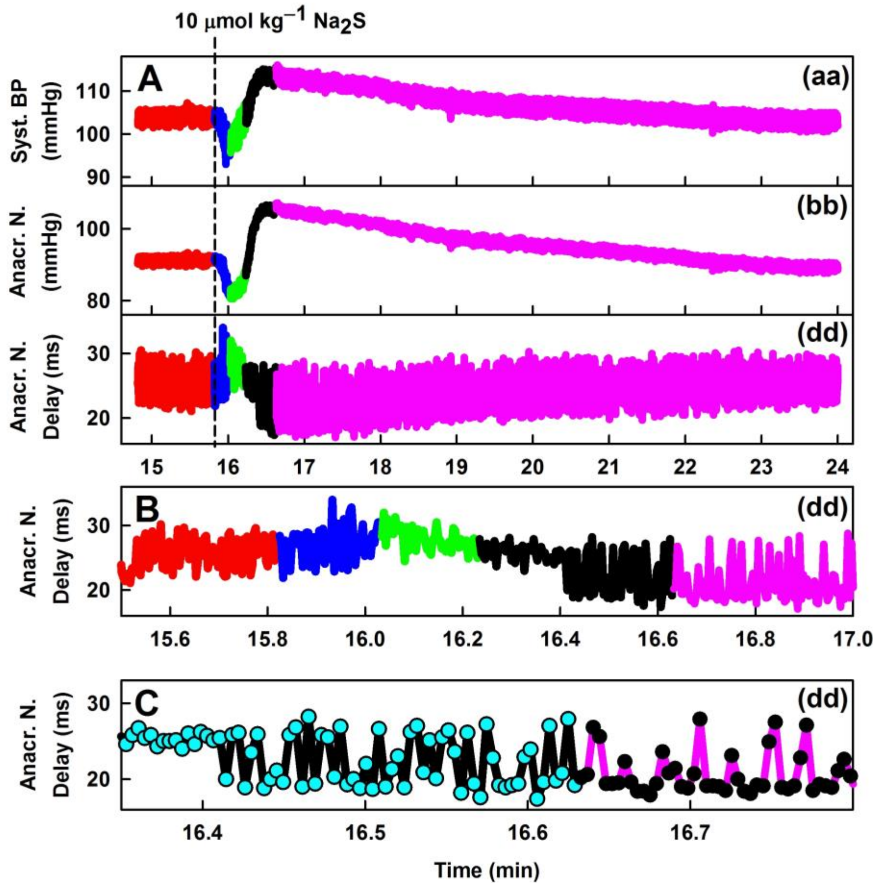

Figure S18. Time-dependent effect of 10 μmol kg−1 Na_2S on three HPs: (**A**): systolic BP (Syst. BP, mmHg) **(aa)**, AnN (Anacr. N., mmHg) **(bb)** and AnN delay (Anacr. N. delay, ms) **(dd);** (15-24 min). (**B**): The fluctuation of AnN delay **(dd);** (16-17 min) reflects the time interval fluctuation between **a1** and **a2** points (Figure S1). The higher value of AnN delay in ms indicates that diastolic BP at the point **a1** was lower than at the point **a2**. (**C**): The example of the fluctuation between **a1** and **a2** points in pulses (circles) showed at high resolution after the 10 μmol kg−1 Na_2S administration **(dd)**. Data were taken from Figure S3.

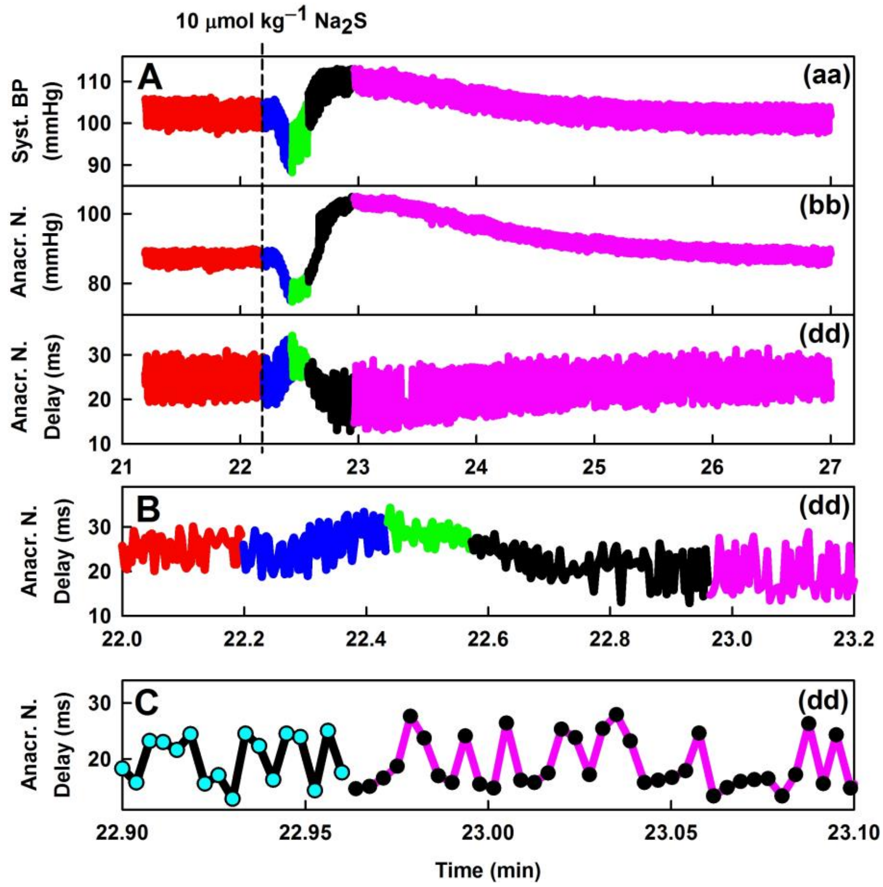

Figure S19. Time-dependent effect of 10 μmol kg−1 Na_2S on three HPs: (**A**): systolic BP (Syst. BP, mmHg) **(aa)**, AnN (Anacr. N., mmHg) **(bb)** and AnN delay (Anacr. N. delay, ms) **(dd);** (21-27 min). (**B**): The fluctuation of AnN delay **(dd);** (22-23 min) reflects the time interval fluctuation between **a1** and **a2** points (Figure S1). The higher value of AnN delay in ms indicates that diastolic BP at the point **a1** was lower than at the point **a2**. (**C**): The example of the fluctuation between **a1** and **a2** points in pulses (circles) showed at high resolution after the 10 μmol kg−1 Na_2S administration **(dd)**. Data were taken from Figure S4.

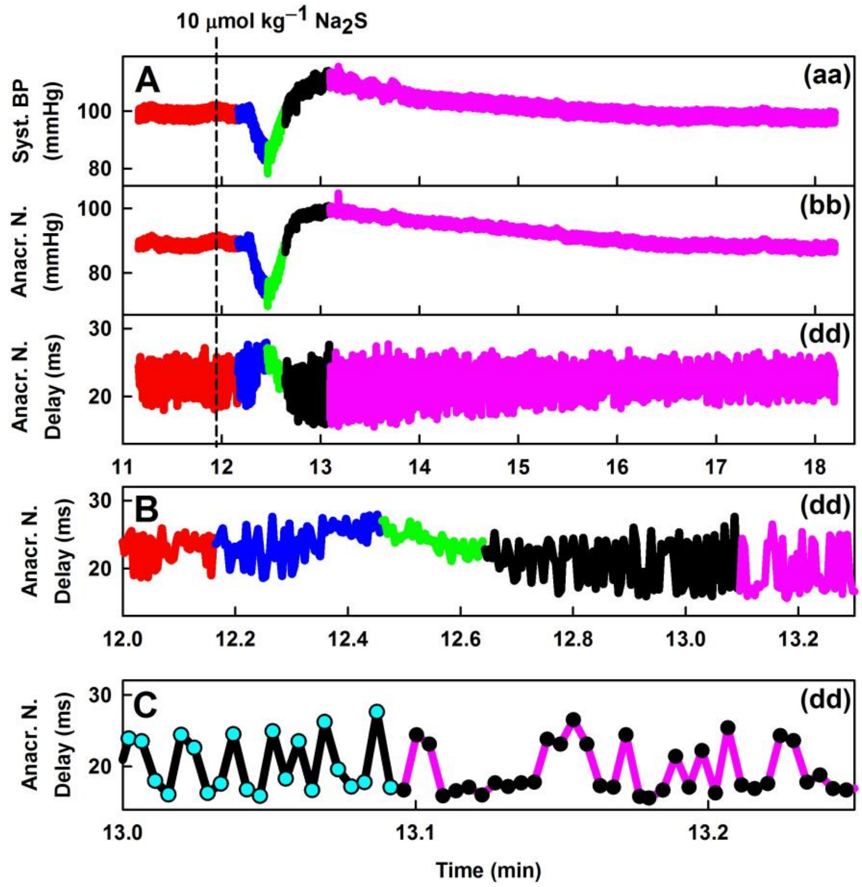

Figure S20. Time-dependent effect of 10 μmol kg^{-1} Na_2S on three HPs: (**A**): systolic BP (Syst. BP, mmHg) **(aa)**, AnN (Anacr. N., mmHg) **(bb)** and AnN delay (Anacr. N. delay, ms) **(dd);** (11-18 min). (**B**): The fluctuation of AnN delay **(dd);** (12-13 min) reflects the time interval fluctuation between **a1** and **a2** points (Figure S1). The higher value of AnN delay in ms indicates that diastolic BP at the point **a1** was lower than at the point **a2**. (**C**): The example of the fluctuation between **a1** and **a2** points in pulses (circles) showed at high resolution after the 10 μmol kg^{-1} Na_2S administration **(dd)**. Data were taken from Figure S5.

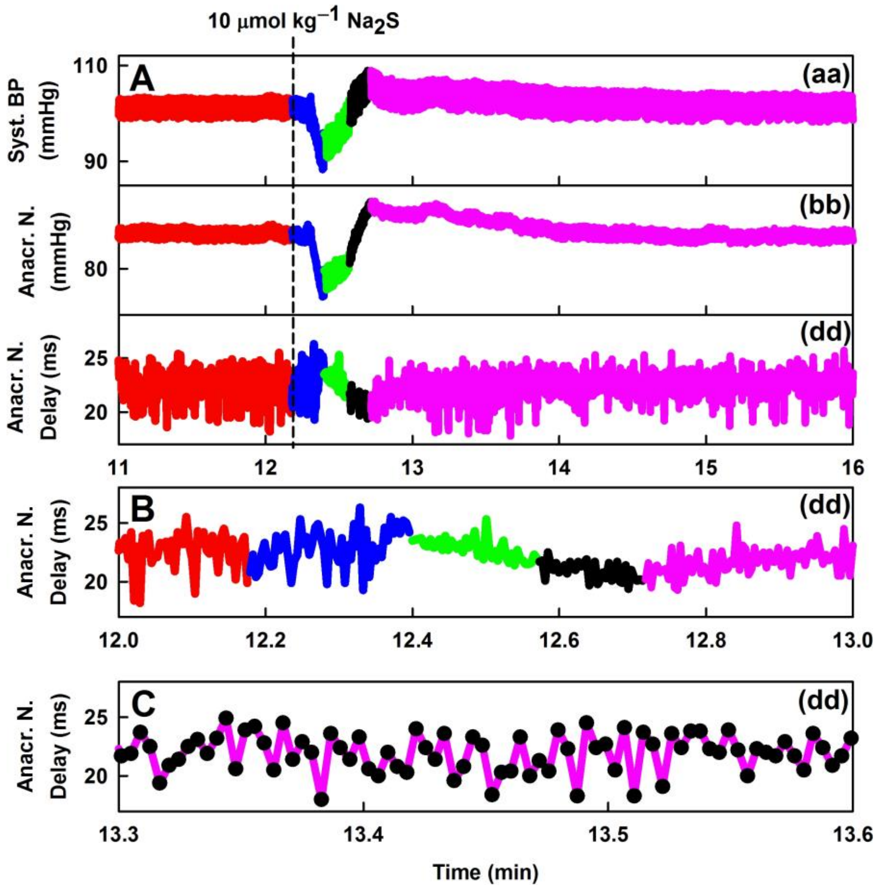

Figure S21. Time-dependent effect of 10 μmol kg⁻¹ Na_2S on three HPs: (**A**): systolic BP (Syst. BP, mmHg) **(aa)**, AnN (Anacr. N., mmHg) **(bb)** and AnN delay (Anacr. N. delay, ms) **(dd)**; (11-16 min). (**B**): The fluctuation of AnN delay **(dd)**; (12-13 min) reflects the time interval fluctuation between **a1** and **a2** points (Figure S1). The higher value of AnN delay in ms indicates that diastolic BP at the point **a1** was lower than at the point **a2**. (**C**): The example of the fluctuation between **a1** and **a2** points in pulses (circles) showed at high resolution after the 10 μmol kg⁻¹ Na_2S administration **(dd)**. Data were taken from Figure S6.

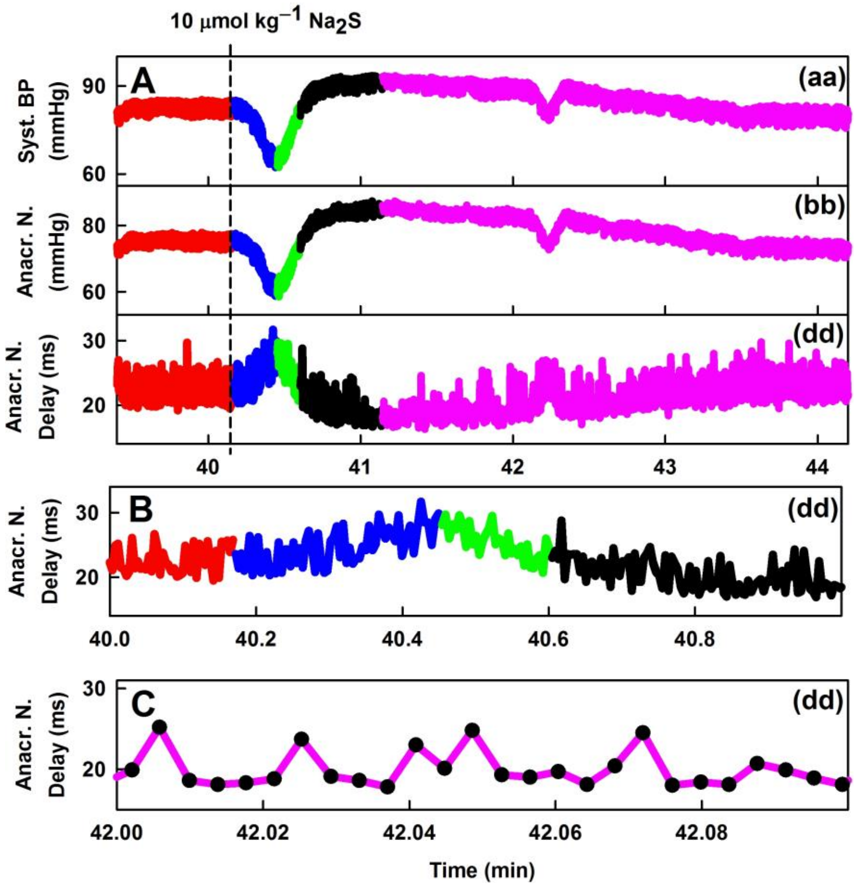

Figure S22. Time-dependent effect of 10 µmol kg^{-1} Na_2S on three HPs: (**A**): systolic BP (Syst. BP, mmHg) **(aa)**, AnN (Anacr. N., mmHg) **(bb)** and AnN delay (Anacr. N. delay, ms) **(dd)**; (40-44 min). (**B**): The fluctuation of AnN delay **(dd)**; (40-41 min) reflects the time interval fluctuation between **a1** and **a2** points (Figure S1). The higher value of anacrotic notch delay in ms indicates that diastolic BP at the point **a1** was lower than at the point **a2**. (**C**): The example of the fluctuation between **a1** and **a2** points in pulses (circles) showed at high resolution after the 10 µmol kg^{-1} Na_2S administration **(dd)**. Data were taken from Figure S7.

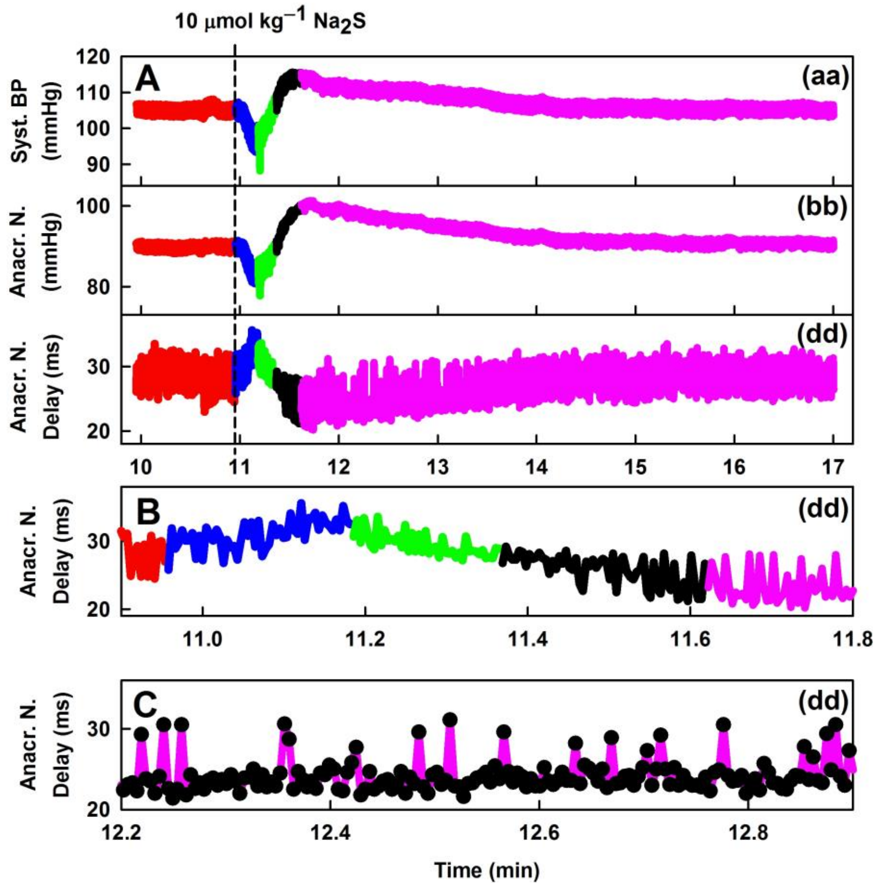

Figure S23. Time-dependent effect of 10 μmol kg[−1] Na_2S on three HPs: (**A**): systolic BP (Syst. BP, mmHg) **(aa)**, AnN (Anacr. N., mmHg) **(bb)** and AnN delay (Anacr. N. delay, ms) **(dd)**; (10-17 min). (**B**): The fluctuation of AnN delay **(dd)**; (11-12 min) reflects the time interval fluctuation between **a1** and **a2** points (Figure S1). The higher value of anacrotic notch delay in ms indicates that diastolic BP at the point **a1** was lower than at the point **a2**. (**C**): The example of the fluctuation between **a1** and **a2** points in pulses (circles) showed at high resolution after the 10 μmol kg[−1] Na_2S administration **(dd)**. Data were taken from Figure 1.

References

1. Kurakova, L.; Misak, A.; Tomasova, L.; Cacanyiova, S.; Berenyiova, A.; Ondriasova, E.; Balis, P.; Grman, M.; Ondrias, K. Mathematical relationships of patterns of 35 rat haemodynamic parameters for conditions of hypertension resulting from decreased nitric oxide bioavailability. *Exp. Physiol.* **2020**, *105*, 312–334, doi:10.1113/ep088148.
2. Misak, A.; Kurakova, L.; Berenyiova, A.; Tomasova, L.; Grman, M.; Cacanyiova, S.; Ondrias, K. Patterns and Direct/Indirect Signaling Pathways in Cardiovascular System in the Condition of Transient Increase of NO. *Biomed. Res. Int.* **2020**, *2020*, 6578213, doi:10.1155/2020/6578213.
